# Effectiveness of health mediation to promote organised cancer screening among underserved and under-screened populations in Marseille, France: findings from a repeated cross-sectional survey

**DOI:** 10.1186/s12889-026-28710-5

**Published:** 2026-08-03

**Authors:** Eva Legendre, Anne Dutrey-Kaiser, Yazid Attalah, Ghislaine Boyer, Steve Nauleau, Jean Gaudart, David Kelly, Céline Caserio-Schönemann, Philippe Malfait, Pascal Chaud, Lauriane Ramalli, Chrystelle Gastaldi, Florian Franke, Stanislas Rebaudet

**Affiliations:** 1 Hôpital Européen, Marseille, France; 2 Prospective et Coopération, Marseille, France; 3 Association Santé Environnement pour Tous (SEPT), Marseille, France; 4 Agence Régionale de Santé Provence-Alpes Côte d’Azur, Marseille, France; 5 Aix Marseille Univ, IRD, INSERM, UMR1252, SESSTIM, APHM, ISSPAM, BioSTIC, Marseille, France; 6 Santé publique France Provence-Alpes-Côte d’Azur et Corse, Marseille, France; 7 SESSTIM, Aix Marseille Univ, IRD, INSERM, ISSPAM, Marseille, France

**Keywords:** Health mediation, Patient navigation, Cancer screening, Breast cancer, Mammography, Colorectal cancer, Faecal immunochemical test, Uterine cervical cancer, Cervical smear, HPV

## Abstract

**Background:**

Since 2022, the “13 en Santé” health mediation program has been implemented in Marseille, France, to reduce social health inequalities, in socioeconomically disadvantage neighbourhoods, with a particular emphasis on promoting participation in population-based organised cancer screening programs. Led by multidisciplinary teams including health mediators, nurses, coordinators, and volunteers, the intervention is structured around three core components: (i) territory exploration to develop knowledge of local communities and resources; (ii) culturally appropriate health education delivered through outreach activities to reach underserved populations; and (iii) individualised navigation support tailored to needs and level of autonomy. This study evaluates the effectiveness of this program in promoting breast, colorectal, and cervical cancer screening.

**Methods:**

A controlled before-after design based on two cross-sectional knowledge-attitude-practice surveys was conducted in 2022 and 2024 in intervention or control neighbourhoods. Individuals aged 18–74 were randomly selected and interviewed via door-to-door questionnaires. Weighting was applied to account for stratified sampling and to align age and sex distributions with census data. For each cancer, weighted logistic regression models assessed associations between health mediation exposure and screening uptake and awareness at both individual and population levels.

**Results:**

Overall, 4,523 individuals were included across the two cross-sectional surveys. The program successfully reached individuals facing cumulative socioeconomic barriers to healthcare access. No significant population-level association was observed between health mediation and cancer screening uptake or awareness. At the individual level, declared exposure to health mediation was associated with significantly higher uptakes of breast and colorectal cancer screenings (breast: 74% vs. 54%, OR = 2.3 [1.1–4.5]; colorectal: 50% vs. 30%, OR = 2.8 [1.3–5.8]). In addition, colorectal cancer screening awareness was significantly higher among exposed participants (93% vs. 83%, OR = 8.1 [2.1–31]).

**Conclusions:**

This study provides the first evidence that reported individual exposure to a multicomponent health mediation intervention was significantly associated with an important increase in breast and colorectal cancer screening uptake in disadvantaged European neighbourhoods. Although the limitations of this observational study preclude causal conclusions regarding the intervention’s effectiveness, these findings suggest that health mediation may be a promising strategy for reducing inequalities in cancer prevention and warrant further investigation. It also highlights screening-specific mechanisms of action that should be considered to further optimise intervention effectiveness.

**Supplementary Information:**

The online version contains supplementary material available at https://doi.org/10.1186/s12889-026-28710-5.

## Background

Reducing social inequalities in health is essential to progress through a more equitable and just society. However, these disparities have increased over the past decade in Europe, particularly in France [1 , 2]. To address its significant territorial and social inequities, France has defined ‘priority neighbourhoods’ (*quartiers prioritaires de la politique de la ville*) focusing resources on areas with high socioeconomic disadvantage [3]. In Marseille, the country’s second-largest city, 28% of the population (243,471 individuals) reside in a priority neighbourhood [4]. These neighbourhoods are concentrated in the city centre (i.e., 1st to 3rd arrondissements) and the northern arrondissements (i.e., 13th to 16th arrondissements), creating a North-South divide. Residents in these priority neighbourhoods have less access to healthcare (e.g., primary care physician, universal health insurance coverage) and preventive services (e.g., cancer screening, vaccination), compared with the rest of the city [5].

In France, cancer is the leading cause of death among men and the second leading cause among women [6]. France offers population-based cancer screening programs for breast cancer for women aged 50–74 years, colorectal cancer for adults aged 50–74, and uterine cervical cancer for women aged 25–64 (Sup. method 1 p.5) [7]. As part of the national screening programs, eligible individuals receive systematically an invitation letter by mail to participate in cancer screening.

Despite the availability of these three cancer screening programs, free of charge for all individual with universal health coverage, uptake has shown substantial territorial and social disparities, as seen in Marseille. First, screening rates in the city are consistently lower than regional and national levels; for example, most recent colorectal cancer screening uptake rates reached 25.1% in Marseille, compared with 30.9% regionally and 34.2% nationally [8 , 9]. Second, differences were observed across screening programs at the city level, with uptake ranging from 25.4% for colorectal cancer (2021–2022) to 47.5% for cervical cancer (2020–2022) and 58.2% for breast cancer (2021–2022) [8]. Third, disparities following clear geographic and socioeconomic gradients were also observed within the city, with lower participation among people with the lowest incomes [8 , 10].

**Fig. 1:**
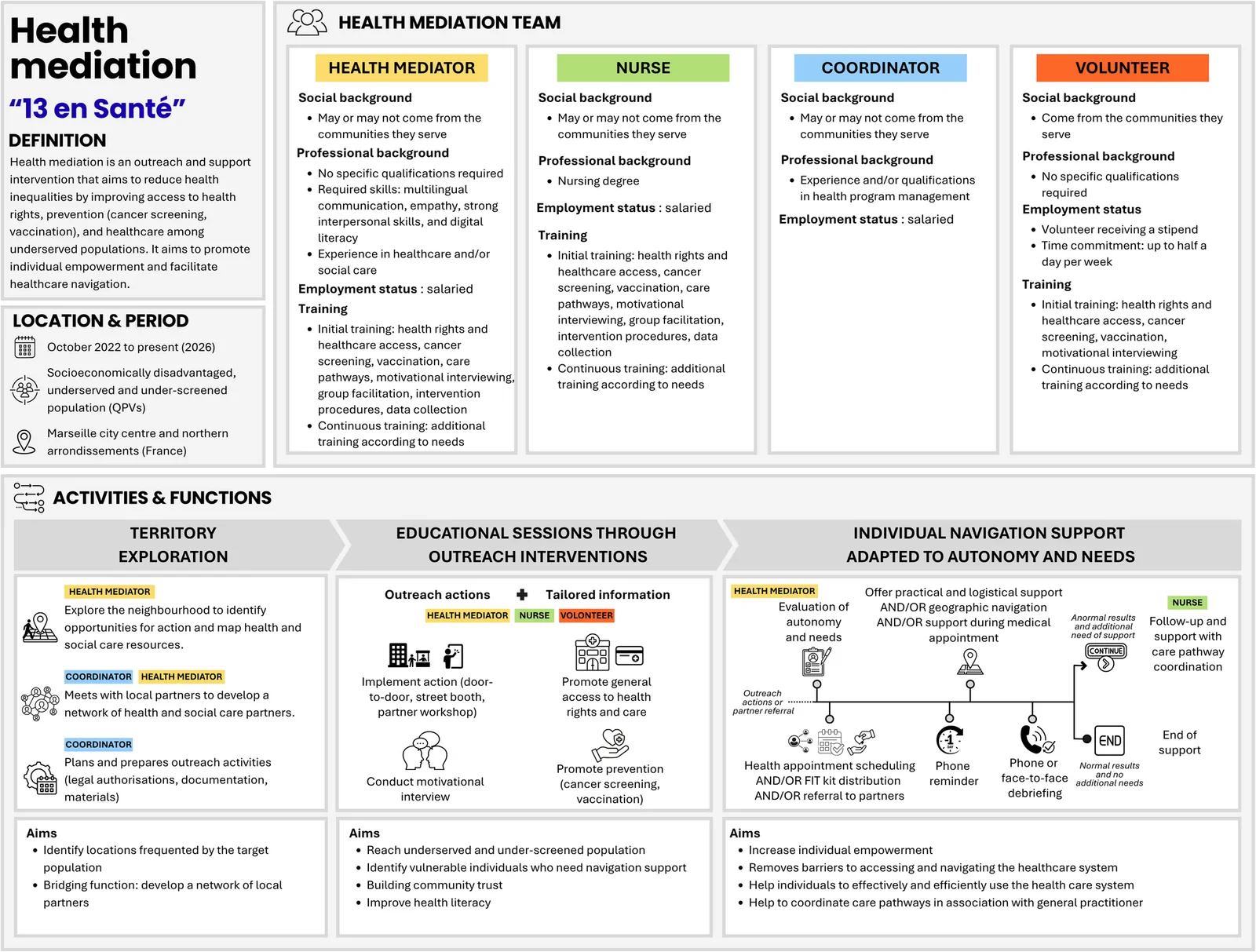
“*13 en Santé*” health mediation program description. FIT, faecal immunochemical test; QPVs, *quartiers prioritaires de la politique de la ville*, i.e. priority neighbourhoods

To reduce health inequalities in the central and northern arrondissements of Marseille, the health mediation program called “*13 en Santé*” was launched in priority neighbourhoods in October 2022 in alignment with regional health authorities and is still ongoing in 2026. Health mediation is a conceptual approach developed in France to reduce social and territorial health inequalities by improving access to health rights, prevention, and healthcare among underserved populations [11 , 12]. Through outreach and support activities, health mediators act as a bridge between these populations and the healthcare system, helping identify needs, facilitate access to services, and navigate care pathways. They rely on a strong knowledge of local communities, stakeholders, and resources. The “*13 en Santé*” health mediation intervention relies on multidisciplinary teams including health mediators, nurses, coordinators, and volunteers (Fig. 1). It is structured around three core components: (i) territory exploration to develop knowledge of local communities and resources; (ii) culturally appropriate health education delivered through outreach activities (door-to-door visits, street stands, and workshops conducted with partner organisations) to reach underserved populations; and (iii) individualised navigation support for people identified as needing assistance, tailored to their needs and level of autonomy. The intervention adopts a broad health approach that addresses social determinants of health by promoting access to care (e.g., health rights, primary and specialist care) and prevention, with a particular emphasis on organised cancer screening programs, while also facilitating referral to social care services.

Although developed in the French context, health mediation shares several functions with community health workers (CHWs) deployed in high-income countries and encompasses patient navigation approaches, both widely studied and used as reference frameworks (see Supplementary method 1 for detailed definitions) [13–15]. An example of the application of these approaches is the European CANCERLESS project, a person-centred and community-based intervention that aims to improve primary and secondary cancer prevention among people experiencing homelessness through a Health Navigator Model relying on navigators with experience working with the target population and strong community knowledge [16]. However, the “*13 en Santé*” intervention extends beyond these approaches. First, although this study focuses on cancer screening, the intervention is not limited to a specific prevention or care pathway but addresses general access to rights, prevention, and healthcare, while relying on a network of health and social care partners to address barriers. Second, it targets a territory rather than a specific population group. Territorial approach implies engagement with diverse populations with different social, cultural, and administrative backgrounds and facing heterogeneous barriers. Third, mediation teams do not necessarily come from the communities they serve, nor do they necessarily possess prior knowledge of the local context. The “13 en Santé” model was therefore developed to: (i) build knowledge of the local territory and develop a local network of partners; (ii) reach underserved populations with diverse backgrounds through a range of outreach strategies; and (iii) provide flexible navigation support, adaptable in both scope and intensity, to address a wide range of health needs and barriers.

Although the effectiveness of health mediation interventions remains poorly documented, the effectiveness of CHW and patient navigation programs in promoting cancer screening uptake has been extensively studied, predominantly in the United States, particularly through randomised controlled trials [17 , 18]. But, their efficacy may be influenced by contextual factors, such as the healthcare system organisation or cancer screening program [17 , 18]. In continental Europe, evidence has remained scarce. Patient navigation interventions have been evaluated in Belgium in 2005–2006 and in France in 2011–2013 to increase breast and colorectal cancer screening uptake, respectively [19 , 20]. Both results showed a modest increase in participation (< 5% percentage points). In the French context, patient navigation also contributed to exacerbated social inequalities in screening rates when implemented in the general population.

This study aims to evaluate the effectiveness of a multicomponent health mediation program in promoting breast, colorectal, and cervical cancer screening among underserved and under-screened populations living in socioeconomically disadvantaged neighbourhoods in Marseille, France, representing, to our knowledge, the first such study in Europe.

## Methods

### Setting

At the request of the regional health authority (*Agence Régionale de Santé Provence-Alpes-Côte d’Azur*,* ARS-Paca*), the “*13 en Santé*” health mediation program has been implemented since October 2022 in Marseille by two operators: CORHESAN, supported by a private nonprofit hospital, Hôpital Européen, and a non-profit organisation, Prospective & Coopération, targeting nine priority neighbourhoods of the city centre; and SEPT, a non-profit organisation, targeting four priority neighbourhoods of the northern arrondissements [21]. Teams have been composed of trained health mediators, nurses, volunteers and coordinators (Fig. 1).

They promote breast, cervical and colorectal cancer screenings through educational sessions delivered using a range of outreach strategies (Sup. method 1 p.5). These outreach activities not only help reach underserved populations but also identify individuals who may benefit from navigation support adapted in terms of scope (health rights, access to healthcare and prevention) and intensity, according to their level of autonomy and specific needs. For cancer screening, navigation support may include assistance in accessing mammography centres, gynaecological consultations, or faecal immunochemical test (FIT) kits, followed by reminder calls before appointments or test completion deadlines. Access to appointments can be facilitated through practical solutions, geographical guidance, and/or accompaniment to healthcare appointments. Phone or face-to-face follow-up is then systematically provided to ensure that individuals have received and understood their screening results. For participants with normal screening results, navigation support ends until the next screening round. In the case of abnormal results, a nurse takes over the navigation process to ensure appropriate diagnostic follow-up and engagement in the recommended care pathway. The duration of navigation support thereafter depends on each individual’s situation.

### Study design

In parallel to this health mediation program, health authorities also commanded an assessment of the intervention effectiveness primarily on cancer screening uptake and secondarily on participants’ awareness. This was conducted using a before-and-after, control-intervention design based on two repeated cross-sectional knowledge-attitude-practice surveys interviewing the general population of 13 intervention and 26 control neighbourhoods. Control neighbourhoods were defined as areas without planned health mediation activities and were selected to be socioeconomically like the intervention neighbourhoods (Sup. method 2, sup. figure 1 p.6). The baseline survey (T0) was conducted before the start of the health mediation program from 18 August to 30 September 2022 in intervention neighbourhoods, and from 28 August to 27 December 2022 in control areas. The follow-up survey (T1) was conducted one year and half after the start of the health mediation program in two phases due to operational reasons: the first phase (T1 P1) from 22 April to 20 June 2024, and the second phase (T1 P2) from 9 to 24 September 2024.

### Participants

Residents aged 18 to 74 years in both intervention and control neighbourhoods were randomly selected through door-to-door visits (Sup. method 3 p.7). While only individuals aged 25 to 74 years were eligible for at least one of the targeted cancer screening programs, the 18–24 age group was also included to evaluate vaccination outcomes. This article focuses on results related to cancer screening promotion.

### Procedures

A knowledge-attitude-practice anonymised questionnaire on cancer screening and vaccination was administered to participants. The questionnaire was specifically developed for this study by adapting and combining items from two national cancer-related surveys conducted by Santé publique France (Sup. method 4 p.8–13) [22 , 23]. Additional items were designed for study-specific objectives. The instrument was intentionally kept simple and accessible to ensure feasibility for door-to-door administration, with socio-economic questions placed at the end to minimise respondent reluctance. Outcomes related to breast, cervical, and colorectal cancer screening awareness and uptake (i.e. screening up to date according to national guidelines) were collected from participants using tablets. Individual information regarding socio-economic characteristics (i.e., self-reported gender, age, birthplace, mother tongue, education level, occupational status, residential status) and general access to healthcare system (i.e., primary care physician, receipt of cancer screening invitation) was also collected. During the T1 follow-up survey, participants were also asked whether they had encountered a CORHESAN or SEPT health mediator in the past and whether cancer screening programs and vaccination had been discussed (Sup. table 1 p.14–15).

### Statistical analysis

Statistical analyses were conducted using R 4.2.2 (R Development Core Team, R Foundation for Statistical Computing, Vienna, Austria) and {ade4}, {survey}, {lme4} and {rpart} packages [24 –26].

#### Study size

The sample size at baseline (T0) and follow-up (T1) was calculated using the estimated baseline cancer screening uptake rates in the targeted arrondissements (46% for breast, 18% for colorectal, and 32% for uterine cervical cancer) [8 , 27]. An expected absolute increase of 10% in uptake was assumed. Calculations accounted for the population structure of both intervention and control neighbourhoods, including overlap among eligible groups [28]. Sample size estimation was performed for a two-sided test with a type I error rate of 5% and a statistical power of 80%. Participant recruitment was stratified by age, gender, and neighbourhood.

#### Socioeconomic profiles construction

To capture cumulative socioeconomic advantages or disadvantages, we constructed participants profiles that summarised key characteristics (birthplace, mother tongue, education level, occupational status, residential status). A socioeconomic profile was defined as a cluster of individuals sharing similar socioeconomic patterns. To create these profiles, a multiple correspondence analysis followed by a hierarchical ascendant clustering was performed, using {ade4} R package [24]. Profile imputation was carried out for individuals with at least one available socioeconomic characteristic using a decision tree built through recursive partitioning, which enabled assigning individuals to the most probable profile based on their available characteristics.

#### Weighting based on census data and stratification sampling

Individual weighting was applied to account for the three-level stratification and the distribution of the general population by age and sex in the included neighbourhoods, based on census data and using {survey} R package [28]. All percentages were estimated using the applied survey weights.

#### Weighted modelling

Weighted logistic regression models were performed separately for breast, colorectal, and cervical cancer screenings to estimate the association between health mediation intervention exposure and screening uptake and awareness at both the population and individual levels. Population-level associations were estimated by comparing screening uptake or awareness between control and intervention neighbourhoods, before and after the implementation of the intervention. Individual-level associations were estimated by comparing participants who reported having encountered a health mediator with those who had not, regardless of their home neighbourhood. Association estimates were adjusted for individual confounding factors in multivariate models, including age, socioeconomic profile, access to a primary care physician, receipt of the screening invitation letter, residential arrondissement, and gender, when appropriate.

Secondary weighted multivariate analyses were conducted to highlight individual characteristics associated with general access to healthcare (i.e., primary care physician, receipt of the screening invitation letter) and with reporting an encounter with a health mediator.

## Results

### Participants description

Overall, 4,530 individuals participated in both cross-sectional surveys. Seven questionnaires were excluded due to missing neighbourhood information. Thus, 4,523 individuals were included in the final analysis of the two cross-sectional surveys and 4,095 (90.54%) were eligible to at least one cancer screening (Table 1). Four socioeconomic profiles were identified and labelled based on their predominant characteristics: “Native speaker renter” (NS renter) regrouping 42.85% of the participants (*n* = 1,938); “Non-native speaker renter” (non-NS renter), with 35.55% (*n* = 1,608); “Native speaker homeowner” (NS homeowner) accounting for 10.90% (*n* = 493); and “Native speaker student” (NS student), representing 10.55% (*n* = 477) (Table 1, sup. figures 2–7 p.16–21).

### Weighted sample

Women aged over 40 years were overrepresented compared with the general population (Sup. figure 8 p.22). To correct for sampling imbalance, a weighting was applied to each participant – by assigning higher weights to younger individuals and to men – which restored an age and sex distribution comparable to the underlying population in each neighbourhood (Sup. figures 9–10 p.23–24). Weighting slightly modified the distribution of socioeconomic profiles, increasing the proportion of NS students in the T1 neighbourhoods (Sup. figure 11 p.25).

**Table 1:** Baseline and follow-up characteristics of participants in control and intervention neighbourhoods (unweighted), n (%)

	Baseline (T0)		Follow-up (T1)		Overall
	Control (*n* = 1,384, 30.60%)	Intervention (*n* = 1,261, 27.88%)	Control (*n* = 981, 21.69%)	Intervention (*n* = 897, 19.83%)	(*n* = 4,523)
Operational information					
Survey phases					
P0 (Aug.-Dec. 2022)	1,384 (100.00)	1,261 (100.00)	0 (0.00)	0 (0.00)	2,645 (58.48)
P1 (April-June 2024)	0 (0.00)	0 (0.00)	889 (90.62)	583 (64.99)	1,472 (32.54)
P2 (Sept. 2024)	0 (0.00)	0 (0.00)	81 (8.26)	281 (31.33)	362 (8.00)
Missing data	0 (0.00)	0 (0.00)	11 (1.12)	33 (3.68)	44 (0.97)
Demographic characteristics					
Gender					
Man	544 (39.31)	500 (39.65)	325 (33.13)	317 (35.34)	1,686 (37.28)
Woman	840 (60.69)	761 (60.35)	655 (66.77)	579 (64.55)	2,835 (62.68)
Missing data^a^	0 (0.00)	0 (0.00)	1 (0.10)	1 (0.11)	2 (0.04)
Age					
18–24	139 (10.04)	138 (10.94)	81 (8.26)	70 (7.80)	428 (9.46)
25–29	146 (10.55)	107 (8.49)	99 (10.09)	87 (9.70)	439 (9.71)
30–34	108 (7.80)	113 (8.96)	69 (7.03)	63 (7.02)	353 (7.80)
35–39	130 (9.39)	129 (10.23)	68 (6.93)	64 (7.13)	391 (8.64)
40–44	92 (6.65)	106 (8.41)	53 (5.40)	50 (5.57)	301 (6.65)
45–49	112 (8.09)	95 (7.53)	61 (6.22)	59 (6.58)	327 (7.23)
50–54	153 (11.05)	158 (12.53)	143 (14.58)	143 (15.94)	597 (13.20)
55–59	141 (10.19)	117 (9.28)	98 (9.99)	83 (9.25)	439 (9.71)
60–64	110 (7.95)	96 (7.61)	117 (11.93)	110 (12.26)	433 (9.57)
65–69	106 (7.66)	88 (6.98)	90 (9.17)	89 (9.92)	373 (8.25)
70–74	147 (10.62)	114 (9.04)	102 (10.40)	79 (8.81)	442 (9.77)
Arrondissement					
1st	86 (6.21)	206 (16.34)	69 (7.03)	170 (18.95)	531 (11.74)
2nd	132 (9.54)	183 (14.51)	91 (9.28)	138 (15.38)	544 (12.03)
3rd	461 (33.31)	240 (19.03)	339 (34.56)	157 (17.50)	1,197 (26.46)
13th	208 (15.03)	185 (14.67)	155 (15.80)	148 (16.50)	696 (15.39)
14th	111 (8.02)	131 (10.39)	64 (6.52)	89 (9.92)	395 (8.73)
15th	243 (17.56)	162 (12.85)	156 (15.90)	105 (11.71)	666 (14.72)
16th	143 (10.33)	154 (12.21)	107 (10.91)	90 (10.03)	494 (10.92)
Socio-economic characteristics					
Birthplace					
France	872 (63.01)	633 (50.20)	574 (58.51)	500 (55.74)	2,579 (57.02)
Abroad	506 (36.56)	621 (49.25)	398 (40.57)	395 (44.04)	1,920 (42.45)
Missing data	6 (0.43)	7 (0.56)	9 (0.92)	2 (0.22)	24 (0.53)
Mother tongue					
French	868 (62.72)	643 (50.99)	641 (65.34)	572 (63.77)	2,724 (60.23)
Other	506 (36.56)	608 (48.22)	331 (33.74)	323 (36.01)	1,768 (39.09)
Missing data	10 (0.72)	10 (0.79)	9 (0.92)	2 (0.22)	31 (0.69)
Education level					
Primary school	171 (12.36)	198 (15.70)	57 (5.81)	105 (11.71)	531 (11.74)
Secondary school	685 (49.49)	564 (44.73)	407 (41.49)	328 (36.57)	1,984 (43.86)
Undergraduate	382 (27.60)	371 (29.42)	384 (39.14)	336 (37.46)	1,473 (32.57)
Postgraduate	102 (7.37)	76 (6.03)	95 (9.68)	68 (7.58)	341 (7.54)
Other	26 (1.88)	29 (2.30)	22 (2.24)	46 (5.13)	123 (2.72)
Missing data	18 (1.30)	23 (1.82)	16 (1.63)	14 (1.56)	71 (1.57)
Occupational status					
Employed	566 (40.90)	468 (37.11)	400 (40.77)	370 (41.25)	1,804 (39.89)
Student	89 (6.43)	82 (6.50)	62 (6.32)	54 (6.02)	287 (6.35)
Homemaker	156 (11.27)	242 (19.19)	154 (15.70)	141 (15.72)	693 (15.32)
Retired	292 (21.10)	236 (18.72)	216 (22.02)	209 (23.30)	953 (21.07)
Unemployed	154 (11.13)	147 (11.66)	70 (7.14)	71 (7.92)	442 (9.77)
Other	87 (6.29)	62 (4.92)	56 (5.71)	45 (5.02)	250 (5.53)
Missing data	40 (2.89)	24 (1.90)	23 (2.34)	7 (0.78)	94 (2.08)
Residential status					
Homeowner	192 (13.87)	122 (9.67)	182 (18.55)	104 (11.59)	600 (13.27)
Renter	989 (71.46)	936 (74.23)	684 (69.72)	653 (72.80)	3,262 (72.12)
Family	142 (10.26)	143 (11.34)	86 (8.77)	114 (12.71)	485 (10.72)
Other	47 (3.40)	33 (2.62)	16 (1.63)	22 (2.45)	118 (2.61)
Missing data	14 (1.01)	27 (2.14)	13 (1.33)	4 (0.45)	58 (1.28)
Socioeconomic profile^b^					
Native speaker homeowner	159 (11.49)	88 (6.98)	156 (15.90)	90 (10.03)	493 (10.90)
Native speaker renter	642 (46.39)	483 (38.30)	426 (43.43)	387 (43.14)	1,938 (42.85)
Native speaker student	150 (10.84)	139 (11.02)	86 (8.77)	102 (11.37)	477 (10.55)
Non-native speaker renter	432 (31.21)	550 (43.62)	309 (31.50)	317 (35.34)	1,608 (35.55)
Missing data	1 (0.07)	1 (0.08)	4 (0.41)	1 (0.11)	7 (0.15)
Health mediation					
Encounter with a health mediator					
Not reported	1,384 (100.00)	1,261 (100.00)	896 (91.34)	748 (83.39)	4289 (94.83)
Reported	0 (0.00)	0 (0.00)	46 (4.69)	117 (13.04)	163 (3.60)
Missing data	0 (0.00)	0 (0.00)	39 (3.98)	32 (3.57)	71 (1.57)
Access to healthcare system					
Primary care physician					
Without	196 (14.16)	175 (13.88)	83 (8.46)	55 (6.13)	509 (11.25)
With	1,183 (85.48)	1,085 (86.04)	894 (91.13)	840 (93.65)	4,002 (88.48)
Missing data	5 (0.36)	1 (0.08)	4 (0.41)	2 (0.22)	12 (0.27)
Breast cancer screening invitation (women aged 50–74 years, *n* = 1,438)					
Not received	45 (11.42)	49 (14.63)	56 (14.74)	44 (13.37)	194 (13.49)
Received	318 (80.71)	248 (74.03)	307 (80.79)	267 (81.16)	1,140 (79.28)
Missing data	31 (7.87)	38 (11.34)	17 (4.47)	18 (5.47)	104 (7.23)
Colorectal cancer screening invitation (men and women aged 50–74 years, *n* = 2,284)					
Not received	117 (17.81)	137 (23.91)	145 (26.36)	147 (29.17)	546 (23.91)
Received	470 (71.54)	363 (63.35)	355 (64.55)	300 (59.52)	1,488 (65.15)
Missing data	70 (10.65)	73 (12.74)	50 (9.09)	57 (11.31)	250 (10.95)
Cervical cancer screening invitation (women aged 25–64 years, *n* = 2,118)					
Not received	187 (30.11)	225 (38.93)	158 (32.92)	117 (26.65)	687 (32.44)
Received	300 (48.31)	239 (41.35)	278 (57.92)	271 (61.73)	1,088 (51.37)
Missing data	134 (21.58)	114 (19.72)	44 (9.17)	51 (11.62)	343 (16.19)
Cancer screening uptake					
Breast cancer (women aged 50–74 years, *n* = 1,438)					
Up to date	191 (48.48)	181 (54.03)	224 (58.95)	142 (43.16)	738 (51.32)
Not up to date	188 (47.72)	140 (41.79)	147 (38.68)	176 (53.50)	651 (45.27)
Missing data	15 (3.81)	14 (4.18)	9 (2.37)	11 (3.34)	49 (3.41)
Colorectal cancer (men and women aged 50–74 years, *n* = 2,284)					
Up to date	209 (31.81)	170 (29.67)	170 (30.91)	129 (25.60)	678 (29.68)
Not up to date	423 (64.38)	375 (65.45)	363 (66.00)	367 (72.82)	1,528 (66.90)
Missing data	25 (3.81)	28 (4.89)	17 (3.09)	8 (1.59)	78 (3.42)
Cervical cancer (women aged 25–64 years, *n* = 2,118)					
Up to date	404 (65.06)	394 (68.17)	340 (70.83)	270 (61.50)	1,408 (66.48)
Not up to date	182 (29.31)	144 (24.91)	125 (26.04)	142 (32.35)	593 (28.00)
Missing data	35 (5.64)	40 (6.92)	15 (3.12)	27 (6.15)	117 (5.52)
Cancer screening awareness					
Breast cancer (women aged 50–74 years, *n* = 1,438)					
Aware	379 (96.19)	312 (93.13)	370 (97.37)	307 (93.31)	1,368 (95.13)
Not aware	13 (3.30)	23 (6.87)	7 (1.84)	21 (6.38)	64 (4.45)
Missing data	2 (0.51)	0 (0.00)	3 (0.79)	1 (0.30)	6 (0.42)
Colorectal cancer (men and women aged 50–74 years, *n* = 2,284)					
Aware	562 (85.54)	456 (79.58)	481 (87.45)	386 (76.59)	1,885 (82.53)
Not aware	93 (14.16)	116 (20.24)	63 (11.45)	116 (23.02)	388 (16.99)
Missing data	2 (0.30)	1 (0.17)	6 (1.09)	2 (0.40)	11 (0.48)
Cervical cancer (women aged 25–64 years, *n* = 2,118)					
Aware	524 (84.38)	472 (81.66)	427 (88.96)	386 (87.93)	1,809 (85.41)
Not aware	94 (15.14)	105 (18.17)	46 (9.58)	48 (10.93)	293 (13.83)
Missing data	3 (0.48)	1 (0.17)	7 (1.46)	5 (1.14)	16 (0.76)

^a^Missing data corresponded to a participant declaring undifferentiated gender, but due to low total headcount and statistical limit, we couldn’t consider this category in our analysis

^b^Variable constructed from the following socioeconomic characteristics: birthplace, mother tongue, educational level, occupational status and residential status

### Health mediator encounter

**Fig. 2:**
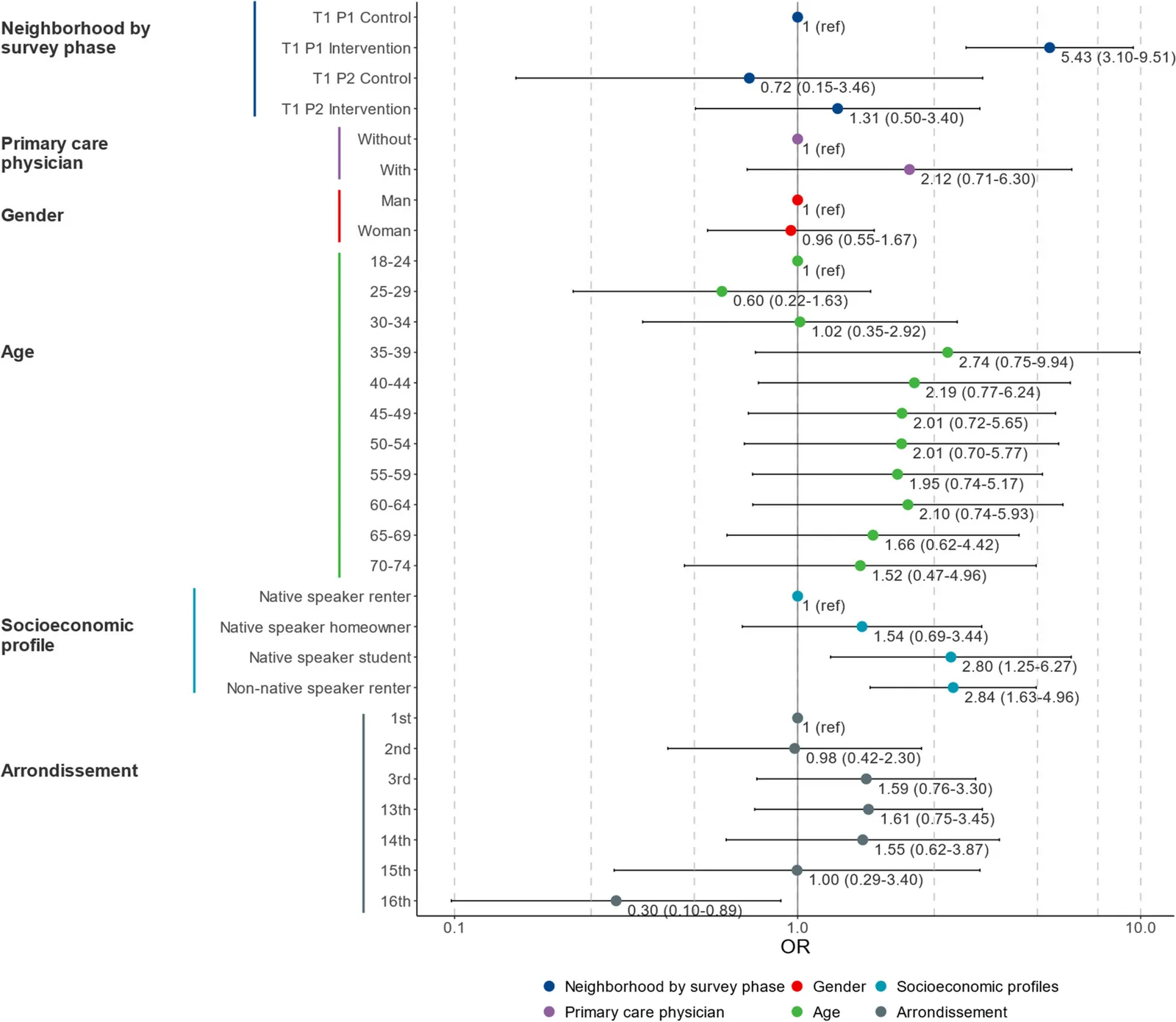
Factors associated with participants reporting an encounter with a health mediator during the T1 survey, in the weighted multivariate analysis

In the T1 survey, 12.78% (*n* = 117) of participants in the intervention neighbourhoods reported having encountered a health mediator, with exposure ranging from 18.21% (*n* = 92) in the central arrondissements to 5.53% (*n* = 25) in the northern neighbourhoods. In the control neighbourhoods, the encounter rate was 3.66% (*n* = 46). Reported health mediator encounters were significantly more frequent in the T1 P1 intervention neighbourhoods (weighted percentage [WP] = 16.62%, *n* = 96) than in the T1 P1 control neighbourhoods (WP = 3.80%, *n* = 42, OR = 5.43 [3.10–9.51]) (Fig. 2, sup. table 2 p.26). In contrast, no significant difference was observed between the T1 P2 intervention neighbourhoods (WP = 3.65%, *n* = 14) and T1 controls (OR = 0.96 [0.43–2.12]). Non-NS renters (WP = 10.81%, *n* = 88, OR = 2.84 [1.63–4.96]) and NS students (WP = 6.26%, *n* = 15, OR = 2.80 [1.25–6.27]) were significantly more likely to report health mediator encounter than NS renters (WP = 3.68%, *n* = 44).

### Population-level association between health mediation and cancer screening uptake

**Fig. 3:**
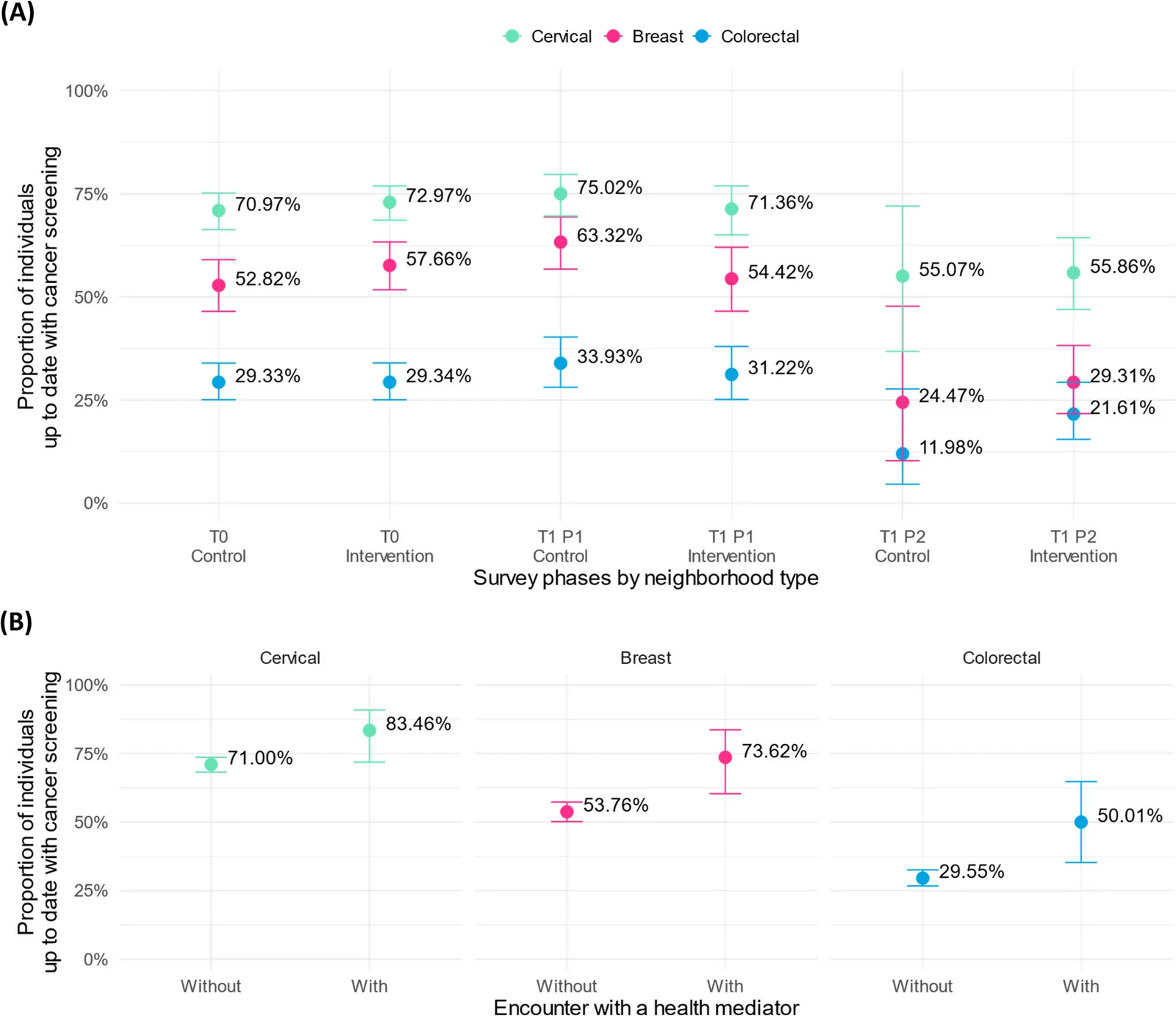
Cancer screening rates by level of exposure to health mediation, defined (**A**) at the population level and, (**B**) at the individual level

**Fig. 4:**
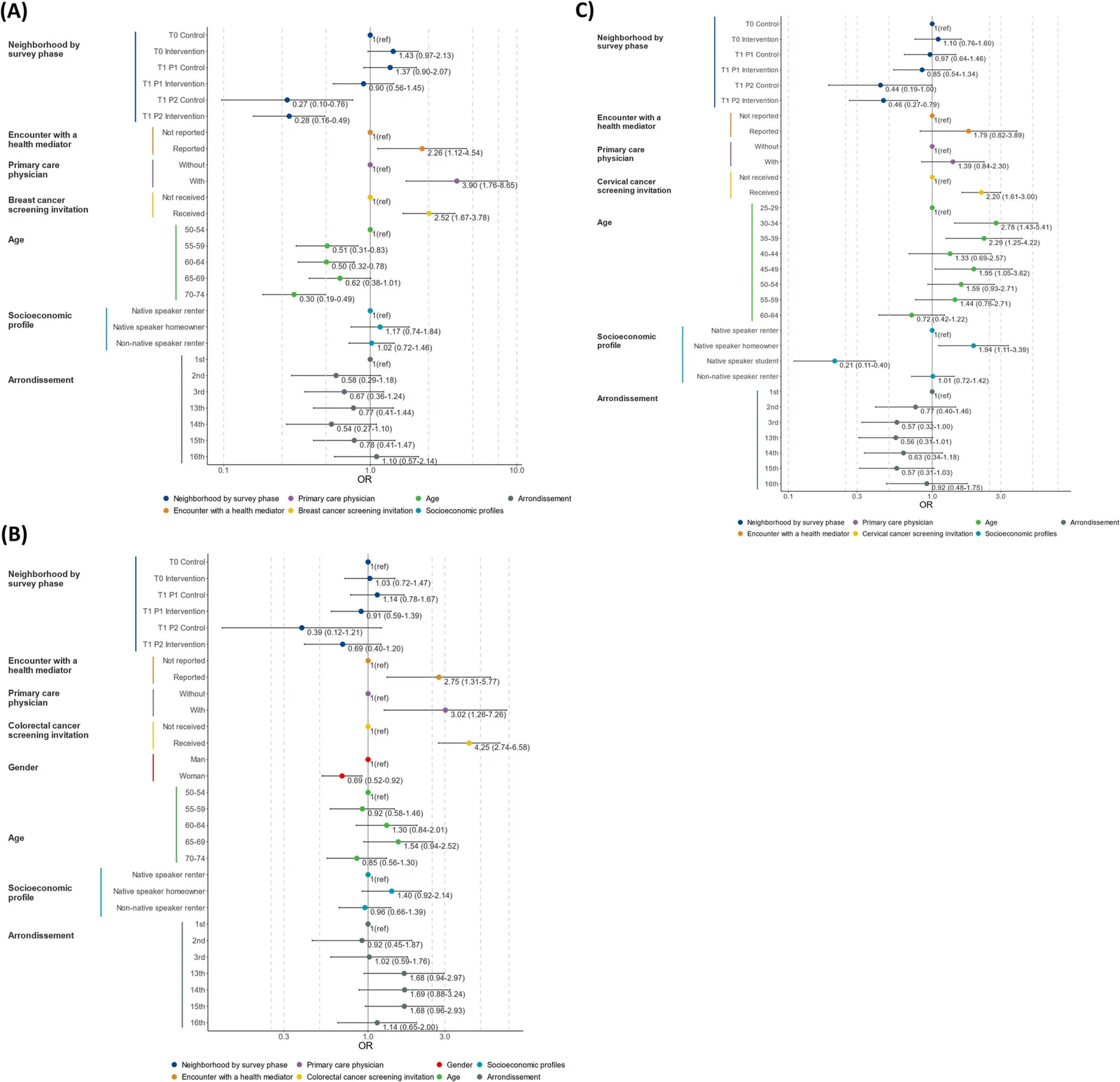
Factors associated with up-to-date cancer screening in multivariate analysis. **A** Breast cancer screening for women aged 50–74 years (*n* = 1,252). **B** Colorectal cancer screening for men and women aged 50–74 years (*n* = 1,925). **C** Cervical cancer screening for women aged 25–64 years (*n* = 1,666)

No significant association with the health mediation program and cancer screening uptake was observed when exposure was defined at the neighbourhood level: for all three cancers, uptake rates remained stable over time − from T0 to T1 − and did not differ between intervention and control neighbourhoods (Figs. 3A and 4, sup. tables 3–5 p.27–29). Overall, reported uptake was highest for cervical cancer screening (WP = 71.34%, *n* = 1,408), followed by breast cancer screening (WP = 54.48%, *n* = 738), and substantially lower for colorectal cancer screening (WP = 29.90%, *n* = 678).

### Individual-level association between health mediation and cancer screening uptake

When exposure was defined at the individual level in the weighted multivariate analysis, participants reporting an encounter with a health mediator reported significantly higher breast (WP = 73.62%, *n* = 42) and colorectal (WP = 50.01%, *n* = 43) cancer screening uptakes compared with those who had not (breast: WP = 53.76%, *n* = 679, OR = 2.26 [1.12–4.54]; colorectal: WP = 29.55%, *n* = 626, OR = 2.75 [1.31–5.77]) (Figs. 3B and 4A-B, sup. tables 3–4 p.27–28). However, although an upward trend was observed, cervical cancer screening uptake was not significantly higher among participants who reported an encounter with a health mediator (encounter: WP = 83.46%, *n* = 72; no encounter: WP = 71%, *n* = 1,319; OR = 1.79 [0.82–3.89]) (Figs. 3B and 4C, sup. tables 5 p.29).

Additionally, age was associated with breast and cervical cancer screening uptake. Women aged over 54 years had significantly lower uptake of breast cancer screening compared with women aged 50–54 years (OR_55−59_=0.51 [0.31–0.83], OR_60−64_=0.50 [0.32–0.78], OR_65−69_=0.62 [0.38–1.01], OR_70−74_=0.30 [0.19–0.49]) (Fig. 4A, sup. table 3 p.27). Regarding cervical cancer screening, women aged 30–39 were more likely to report being up to date than younger women (OR_30−34_=2.78 [1.43–5.41], OR_35−39_=2.29 [1.25–4.22]) (Fig. 4C, sup. table 5 p.29).

### Cancer screening awareness

At population level, health mediation was not associated with an increase in cancer screening awareness (Sup. tables 6–8, sup. figures 12–14 p.30–35).

At individual level, participants reporting an encounter with a health mediator had significantly higher colorectal cancer screening awareness (WP = 93.43%, *n* = 86) compared with those who had not (WP = 82.81%, *n* = 1,753, OR = 8.07 [2.10-30.96]) (Sup. table 6, sup. figure 12 p.30–31). No significant effect of health mediation at individual level was observed for breast and cervical cancer screening awareness (breast: OR = 3.24 [0.43–24.16], cervical: OR = 2.24 [0.64–7.85]) (sup. tables 7–8, sup. figures 13–14 p.32–35). However, the global awareness rate of breast cancer screening was very high, with 96.63% of women reporting that they were aware of the national program. Awareness reached 83.35% for colorectal cancer screening, and 86.72% for cervical cancer screening. Non-NS renters reported lower awareness rates for all three cancer screenings compared with NS renters (breast: OR = 0.39 [0.16–0.96]; colorectal: OR = 0.34 [0.21–0.54]; cervical: OR = 0.26 [0.16–0.44]) (Sup. tables 6–8, sup. figures. 12–14 p.30–35).

### Reception of screening invitation letters

Only the receipt of screening invitation letters was significantly associated with an increased uptake for the three cancers (breast: WPs = 58.83% vs. 38.81%, OR = 2.52 [1.67–3.78]; colorectal: WPs = 38.88% vs. 11.92%, OR = 4.25 [2.74–6.58], cervical: WPs = 79.05% vs. 64.04%, OR = 2.20 [1.61-3.00]) (Fig. 4, sup. tables 3–5 p.27–29). It was also consistently associated with a significant increase in cancer screening awareness across the three programmes (breast: 98.08% vs. 91.49%, OR = 4.08 [2.02–8.27]; colorectal: 96.71% vs. 56.49%, OR = 20.77 [12.57–34.34], cervical: 94.28% vs. 76.54%, OR = 4.30 [2.78–6.65]) (Sup. tables 6–8, sup. figures 12–14 p.30–35).

Overall, the reported invitation letter receipt rate was highest for breast cancer screening, with 80.97% of women (*n* = 1,140), followed by colorectal cancer screening at 65.15% (*n* = 1,488) and cervical cancer screening (WP = 53.30%, *n* = 1,088) (Sup. tables 9–11 p.36,38,40). For all three cancer screenings, non-NS renters consistently reported significantly lower invitation letter receipt compared with NS renters (breast: WPs = 77.31% vs. 89.55%, OR = 0.38 [0.24–0.60]; colorectal: WPs = 57.75% vs. 77.78%, OR = 0.39 [0.28–0.55]; cervical: WPs = 52.41% vs. 69.33%, OR = 0.45 [0.33–0.61]) (Sup. tables 9–11, sup. figures 15–17 p.36–41). On the contrary, individuals who reported access to a primary care physician reported significantly more invitation letter receipt compared with those who did not report access to a physician (breast: WPs = 86.68% vs. 63.50%, OR = 4.46 [2.24–8.89]; colorectal: WPs = 73.73% vs. 48.36%, OR = 3.29 [1.85–5.85]; cervical: WPs = 63.63% vs. 40.21%, OR = 2.57 [1.59–4.14]). Additionally, encountering a health mediator was significantly associated with receipt of the screening invitation only for cervical cancer (WP = 78.80%, *n* = 65), compared with participants who did not report such an encounter (WP = 60.93%, *n* = 1,008, OR = 2.00 [1.03–3.88]).

### Access to primary care physician

Reported access to a primary care physician was also significantly associated with increased uptake in breast (with physician: WP = 55.96%, *n* = 721; without: WP = 29.75%, *n* = 17; OR = 3.90 [1.76–8.65]) and colorectal cancer (with physician: WP = 31.35%, *n* = 667; without: WP = 10.34%, *n* = 10; OR = 3.02 [1.26–7.26]), but not with cervical cancer screening (OR = 1.39 [0.84–2.30]) (Fig. 4, sup. tables 3–5 p.27–29). It was also significantly associated with greater awareness of breast cancer screening (with physician: WP = 97.05%, *n* = 1,307; without: WP = 89.28%, *n* = 58; OR = 2.77 [1.03–7.48]), but not of colorectal (with physician: WP = 84.08%, *n* = 1,792; without: WP = 73.67%, *n* = 91; OR = 0.89 [0.41–1.94]) or cervical (with physician: WP = 87.67%, *n* = 1,677; without: WP = 78.16%, *n* = 130; OR = 1.19 [0.66–2.16]) cancer screening (Sup. tables 6–8, sup. figures 12–14 p.30–35).

Most participants (WP = 85.14%, *n* = 4,000) reported having access to a primary care physician (Sup. table 12 p.42). Access to a physician was significantly lower among non-NS renters (WP = 82.07%, *n* = 1,403) compared with NS renters (WP = 88.81%, *n* = 1,764, OR = 0.58 [0.42–0.79]) (Sup. table 12, sup. figure 18 p.42–43).

## Discussion

This repeated cross-sectional study showed that, although no association between the “*13 en Santé*” health mediation program and cancer screening uptake was observed at the population level, reported encounter with a health mediator was significantly associated with an increase in individual uptake of breast and colorectal cancer screening, with a 20-percentage-point gain. As demonstrated by the multivariate analysis, it may suggest a complementary role of health mediation alongside access to a primary care physician and receipt of the screening invitation letters.

The 20-percentage-point increase in cancer screening uptake associated with health mediation is consistent with previous studies, although slightly higher than reported median effects. Although focused on CHW interventions in high-income countries, a 2023 systematic review of effectiveness studies reported a median increase of 11.5% points for breast cancer screening (interquartile range: 5.5–23.5) and a slightly lower median increase of 10.5% points for colorectal cancer screening (interquartile range: 4.5–17.5) [17]. In contrast, despite an upward trend, no significant effect was observed in our study for cervical cancer screening, whereas this review also reported a median increase of 12.8% points (interquartile range: 6.4–21.0). A 2017 meta-analysis similarly demonstrated beneficial effects of community-based interventions across breast, colorectal, and cervical cancer screenings (OR = 1.90 [1.60–2.26]) [29]. A reported encounter with a health mediator was associated with receipt of a screening invitation only for cervical cancer, which may suggest less precise targeting toward women already engaged with preventive care. Consistently, women aged 30–39 − who are more likely to have regular gynaecological follow-up related to childbearing − appeared more often up to date than other eligible women, indicating that targeting younger and older age groups may enhance intervention effectiveness.

By combining territory exploration, educational sessions delivered through outreach activities, and tailored individual navigation support, the “*13 en Santé*” health mediation program may promote cancer screening through multiple mechanisms of action and by mobilizing diverse levers. First, the “*13 en Santé*” health mediation program successfully targeted individuals facing cumulative socioeconomic barriers to accessing health care (i.e., non–French native speakers, those with the lowest levels of education, and those with the highest rates of unemployment or homemaking) [30]. These individuals reported the lowest access to a primary care physician and the lowest receipt rates of cancer screening invitation letters. The reduced receipt rate may reflect an actual lower likelihood of receiving the letter − for example, due to unstable housing − or difficulties in understanding the content of the invitation. Second, providing tailored educational sessions or information − using motivational interviewing techniques − may enhance intrinsic motivation by addressing three fundamental needs (competence, autonomy, and relatedness) and can be further reinforced through navigation support when needed [29 , 31]. Additionally, navigation support helps address contextual barriers − whether perceived or real, social or related to accessing the healthcare system − that may hinder individuals from translating intention into action [31 –33]. As a matter of fact, health mediation appeared associated with increased awareness only for colorectal cancer screening, whereas breast cancer awareness was already very high in our sample. This suggests differential mechanisms of action: for colorectal cancer, both raising awareness through tailored education and providing navigation support to overcome barriers appear necessary, while for breast cancer, the intervention may act primarily by helping individuals resolve practical barriers rather than by increasing awareness. No associations were observed between health mediation or primary care physician access and cervical cancer screening uptake. The absence of an association between primary care physician access and cervical cancer screening uptake in French urban settings is consistent with literature [34]. This finding highlights the central role of specialists − gynaecologists and midwives − in this screening pathway and underscores that its uptake driving mechanisms differ from those for breast and colorectal cancers. Further research is needed to identify the specific mechanisms that should be integrated into health mediation strategies to improve their effectiveness in promoting cervical cancer screening.

This study has a major strength in enabling assessment of the intervention through exposure defined at both the individual and neighbourhood levels, allowing simultaneous evaluation of individual- and population-level effects.

It also has several limitations. First, although the intervention was concentrated within the designated intervention neighbourhoods, it may not have been sufficient to prevent dilution effects across time and space. In the northern intervention neighbourhoods − where activities were primarily conducted through workshops and street booths − health mediators may have interacted with individuals residing in adjacent areas, including control or non-included neighbourhoods, thereby diluting the measurable effect of the intervention. Second, although 4,523 individuals were interviewed, it represented only about 6% of the total population of the included neighbourhoods. Third, the T1 follow-up survey was conducted in two phases for operational reasons (i.e., completion of missing questionnaires in Phase 2 due to time constraints during Phase 1), which may have influenced observed screening uptakes. Although survey phase was accounted for in the statistical models, this two-phase data collection may have reduced statistical power to detect population-level effects. Combined with the potential dilution effects described above and the limited population coverage, this may have constrained the study’s ability to identify an effect at the population level. In addition, questionnaire administration relied on door-to-door visits, which aligned with the main outreach strategy in the city centre but not in the northern neighbourhoods. This discrepancy may partly explain the lower reported encounter rates in Northern areas, as interviewed individuals may have been less representative of the populations reached by the intervention, thereby potentially limiting the observed effectiveness of the program. Other limitations are inherent to surveys based on self-reported outcomes. First, recall bias may have led to underestimation of health mediator encounters, limiting the assessment of intervention effects. Second, social desirability bias may have resulted in overestimation of screening uptake rates. Finally, a limitation of this study is inherent to observational quasi-experimental design, which does not allow for establishing a causal relationship between exposure to the intervention and increasing screenings uptake.

## Conclusions

This study provides the first evidence that reported individual exposure to a multicomponent health mediation intervention was significantly associated with an important increase in breast and colorectal cancer screening uptake among underserved and under-screened populations living in socioeconomically disadvantaged urban neighbourhoods in Europe. Although the absence of population-level effects and the inherent limitations of observational studies preclude drawing causal conclusions regarding the intervention’s effectiveness, these findings position health mediation as a promising strategy to reduce persistent inequalities in cancer prevention. Several studies are currently underway, including: (i) an implementation study investigating potential dilution effects; (ii) an effectiveness evaluation using comprehensive medico-administrative data from the French National Health Insurance; and (iii) refinement and updating of the intervention’s theoretical framework. They will be necessary to strengthen effectiveness evidence, refine intervention mechanisms, enhance scalability, and ultimately maximise the impact of health mediation on screening uptake and population health.

## Supplementary Information

Supplementary Material 1.

## Data Availability

Data will be made available upon reasonable request by contacting the corresponding author.

## Abbreviations

ARS Paca: Agence Régionale de Santé Provence-Alpes-Côte d’Azur
CHW: Community health worker
FIT: Faecal immunochemical test
HPV: Human Papilloma Virus
NS: Native speaker
OR: Odds ratio
QPVs: Quartiers prioritaires de la politique de la ville, i.e. priority neighbourhoods
SpF: Santé publique France
WP: Weighted percentage
