# Supplementary material for: Effectiveness of health mediation to promote organised cancer screening among underserved and under-screened populations in Marseille, France: findings from a repeated cross-sectional survey
Source: BMC Public Health. 2026 Aug 3;26:3129. doi: 10.1186/s12889-026-28710-5 (PMC13721444; doi:10.1186/s12889-026-28710-5)
Supplement: Supplementary file 1 [file 12889_2026_28710_MOESM1_ESM.pdf]

APPENDIX

Effectiveness of health mediation to promote organized cancer screening among underserved and under-screened populations in Marseille, France: findings from a repeated cross-sectional survey

Eva Legendre[1], Anne Dutrey-Kaiser[2], Yazid Attalah[3], Ghislaine Boyer[2], Steve Nauleau[4], Jean Gaudart[2,5], David Kelly[6], Céline Caserio-Schönemann[6], Philippe Malfait[6], Pascal Chaud[6], Lauriane Ramalli[6], Chrystelle Gastaldi[4], Florian Franke[6], Stanislas Rebaudet[1,7]

[1], Hôpital Européen, Marseille, France

[2], Prospective et Coopération, Marseille, France

[3], Association Santé Environnement pour Tous, Marseille France

[4], Agence Régionale de Santé Provence-Alpes Côte d'Azur, Marseille, France

[5], Aix Marseille Univ, IRD, INSERM, UMR1252, SESSTIM, APHM, ISSPAM, BioSTIC, Marseille, France

[6], Santé publique France Provence-Alpes-Côte d'Azur et Corse, Marseille, France

[7], SESSTIM, Aix Marseille Université, IRD, INSERM, ISSPAM, Marseille, France

Corresponding author: Eva Legendre, eva.legendre25@hotmail.fr

TABLE OF CONTENTS

METHODS **5**

Appendix 1. Study design and setting **5**

Supplementary method 1. The "*13-en-*Santé" health mediation intervention 5

Supplementary method 2. Selection of intervention and control neighbourhoods 6

Supplementary figure 1. Intervention and control neighbourhoods included in the study, located in the city-centre (1st to 3rd arrondissement) and the Northern arrondissements of Marseille (13th to 16th arrondissements), France. *6*

Appendix 2. Participants **7**

Supplementary method 3. Stratification sampling method and participants' recruitment 7

Appendix 3. Procedures **8**

Supplementary method 4. Questionnaire knowledge-attitude-practice on cancer screening and vaccination 8

Supplementary table 1. Variables' description and construction *14*

RESULTS **16**

Appendix 4. Socioeconomic profiles construction and description **16**

4.1. Socioeconomic profiles construction 16

Supplementary figure 2. Distribution of individuals on the first two axes of the multiple correspondence analysis by variable categories. *16*

Supplementary figure 3. Hierarchical agglomerative clustering after multiple correspondence analysis. (A) Dendrogram and inertia. (B) Distribution of individuals on the first two axes of the multiple correspondence analysis by the four socioeconomic profiles identified by the clustering. *17*

4.2. Socioeconomic profiles description before imputation 18

Supplementary figure 4. Socioeconomic characteristics of participants in the four profiles identified by clustering before missing data imputation. *18*

Supplementary figure 5. (A) Age and (B) gender distribution across the four socioeconomic profiles. *19*

4.3. Socioeconomic profile imputation for missing data 19

Supplementary figure 6. Regression tree of profiles based on socioeconomic variables (n=4,334). *20*

4.4. Socioeconomic profiles description after imputation 21

Supplementary figure 7. Socioeconomic characteristics of participants in the four profiles identified by clustering after missing data imputation. *21*

Appendix 5. Imbalance between general population and the sample surveyed and weighting **22**

Supplementary figure 8. Comparison of age pyramids for the general population (wide light-coloured bars) and the survey samples (narrow dark-coloured bars) in (A) T0 control, (B) T0 intervention, (C) T1 control, and (D) T1 intervention neighbourhoods. *22*

Supplementary figure 9. Weight distribution of the surveyed sample according to (A) gender and (B) age. *23*

Supplementary figure 10. Percentage point difference of age and gender distributions between surveyed sample and general population, according to neighbourhood type and survey time, before and after weighting. *24*

Supplementary figure 11. Proportion of socioeconomic profiles in the surveyed sample before and after weighting, according to neighbourhood type and survey time. *25*

Appendix 6. Encounter with a health mediator **26**

Supplementary table 2. Factors associated with individuals reporting an encounter with a health mediator during the T1 survey. *26*

Appendix 7. Cancer screening uptake **27**

7.1. Breast cancer screening uptake 27

Supplementary table 3. Factors associated with up-to-date breast cancer screening. *27*

7.2. Colorectal cancer screening uptake 28

Supplementary table 4. Factors associated with up-to-date colorectal cancer screening. *28*

7.3. Cervical cancer screening uptake 29

Supplementary table 5. Factors associated with up-to-date cervical cancer screening. *29*

Appendix 8. Cancer screening awareness **30**

8.1. Colorectal cancer screening awareness 30

Supplementary table 6. Factors associated with awareness about colorectal cancer screening. *30*

Supplementary figure 12. Factors associated with awareness about colorectal cancer screening in multivariate analysis (n=1,959). *31*

8.2. Breast cancer screening awareness 32

Supplementary table 7. Factors associated with awareness about breast cancer screening. *32*

Supplementary figure 13. Factors associated with awareness about breast cancer screening in multivariate analysis (n=1,282). *33*

8.3. Cervical cancer screening awareness 34

Supplementary table 8. Factors associated with awareness about cervical cancer screening. *34*

Supplementary figure 14. Factors associated with awareness about cervical cancer screening in multivariate analysis (n=1,727). *35*

Appendix 9. Cancer screening invitation **36**

9.1. Breast cancer screening invitation 36

Supplementary table 9. Factors associated with reception of breast cancer screening invitation. *36*

Supplementary figure 15. Factors associated with reception of breast cancer screening invitation, in the multivariate analysis (n=1,284). *37*

9.2. Colorectal cancer screening invitation 38

Supplementary table 10. Factors associated with reception of colorectal cancer screening invitation. *38*

Supplementary figure 16. Factors associated with colorectal cancer screening invitation reception in multivariate analysis (n=1,963). *39*

9.3. Cervical cancer screening invitation 40

Supplementary table 11. Factors associated with reception of cervical cancer screening invitation. *40*

Supplementary figure 17. Factors associated with cervical cancer screening invitation reception in multivariate analysis (n=1,731). *41*

Appendix 10. Primary care physician 42

Supplementary table 12. Factors associated with access to a primary care physician. *42*

Supplementary figure 18. Factors associated with individuals with a primary care physician, in the weighted multivariate analysis (n=4,401). *43*

METHODS

Appendix 1. Study design and setting

Supplementary method 1. The "*13-en*-Santé" health mediation intervention

Although developed in the French context, health mediation shares several functions with community health workers (CHWs) deployed in high-income countries and encompasses patient navigation approaches, both widely studied and used as reference frameworks in the international literature.

Patient navigation has been defined by Mosquera et al.[1] as a patient-centred intervention designed to improve access to care among hard-to-reach populations by helping individuals overcome barriers to healthcare. It is typically delivered within a defined care pathway. It targets the services required to achieve a specific health objective, addresses barriers at multiple levels (health system, individual, educational, social, and emotional), and aims to improve timely access to care and service uptake.

CHWs have been defined by Olaniran et al.[2] as "paraprofessionals or lay individuals with an in-depth understanding of community culture and language, who receive standardized, short-duration training compared with health professionals, and whose primary goal is to provide culturally appropriate health services to the community". In high-income countries, they may promote healthy lifestyles and disease prevention, encourage the use of available health services, and facilitate access to healthcare, including through patient navigation activities.

The "*13-en*-Santé" program has aimed to promote access to healthcare and preventive services, including population-based cancer screenings programs. France offers free screening programs for breast cancer (mammography targeting women aged 50–74 years, every two years), colorectal cancer (faecal immunochemical test [FIT] for adults aged 50–74 years, every two years), and uterine cervical cancer (two Pap smears one-year apart and a third three years later for women aged 25–29 years, then a high risk human papillomavirus (HR-HPV) PCR every five years for women aged 30-64).[3]

Because of territorial specificities, CORHESAN mainly conducted door-to-door outreach visits, whereas SEPT primarily implemented workshops within partner organizations in the northern arrondissements, where door-to-door activities can be unsafe, and where a strong partner network was already established.[4]

[1] Mosquera I, Todd A, Balaj M, *et al.* Components and effectiveness of patient navigation programmes to increase participation to breast, cervical and colorectal cancer screening: A systematic review. *Cancer Medicine* 2023; **12**: 14584–611.

[2] Olaniran A, Smith H, Unkels R, Bar-Zeev S, van den Broek N. Who is a community health worker? – a systematic review of definitions. Glob Health Action 2017; 10: 1272223.

[3] Institut national du cancer. Official website of national cancer screening programs 'Je fais mon dépistage'. Je fais mon dépistage - Institut national du cancer. https://jefaismondepistage.cancer.fr/ (accessed Dec 2, 2025).

[4] Magnani C, de Mongolfier S, Legendre E, Rebaudet S, Mininel F. Health mediation in Marseille to promote cancer screening: settings, experiences and autonomy. *Submitted* 2025.

Supplementary method 2. Selection of intervention and control neighbourhoods

Intervention neighbourhoods were selected by the two operators based on their field experience and local knowledge, using criteria such as neighbourhood safety and practical accessibility (e.g., areas not restricted by territorial tensions) and the strength of the local community network, to ensure feasible and rapid implementation of health mediation activities. Thirteen neighbourhoods were selected: four in the northern area (one in each of the 13th, 14th, 15th and 16th arrondissements), representing 10,983 inhabitants, and nine in the city-centre (three adjacent neighbourhoods within each of the 1st, 2nd and 3rd arrondissements) covering a target population of 14,098 inhabitants.

Control neighbourhoods were selected based on five criteria: non-contiguity with any intervention neighbourhood to avoid action dilution; proximity to the arrondissement of the corresponding intervention neighbourhood; socioeconomic similarity; and operational accessibility for the teams. Neighbourhoods' socioeconomic similarity was assessed using a principal component analysis-based distance computed from census indicators, including population density, age structure, socioeconomic categories, proportions of foreign-born or immigrant residents, education level, unemployment rate, median income, proportion of manual workers, the 2015 EDI deprivation score, and housing overcrowding.[5]

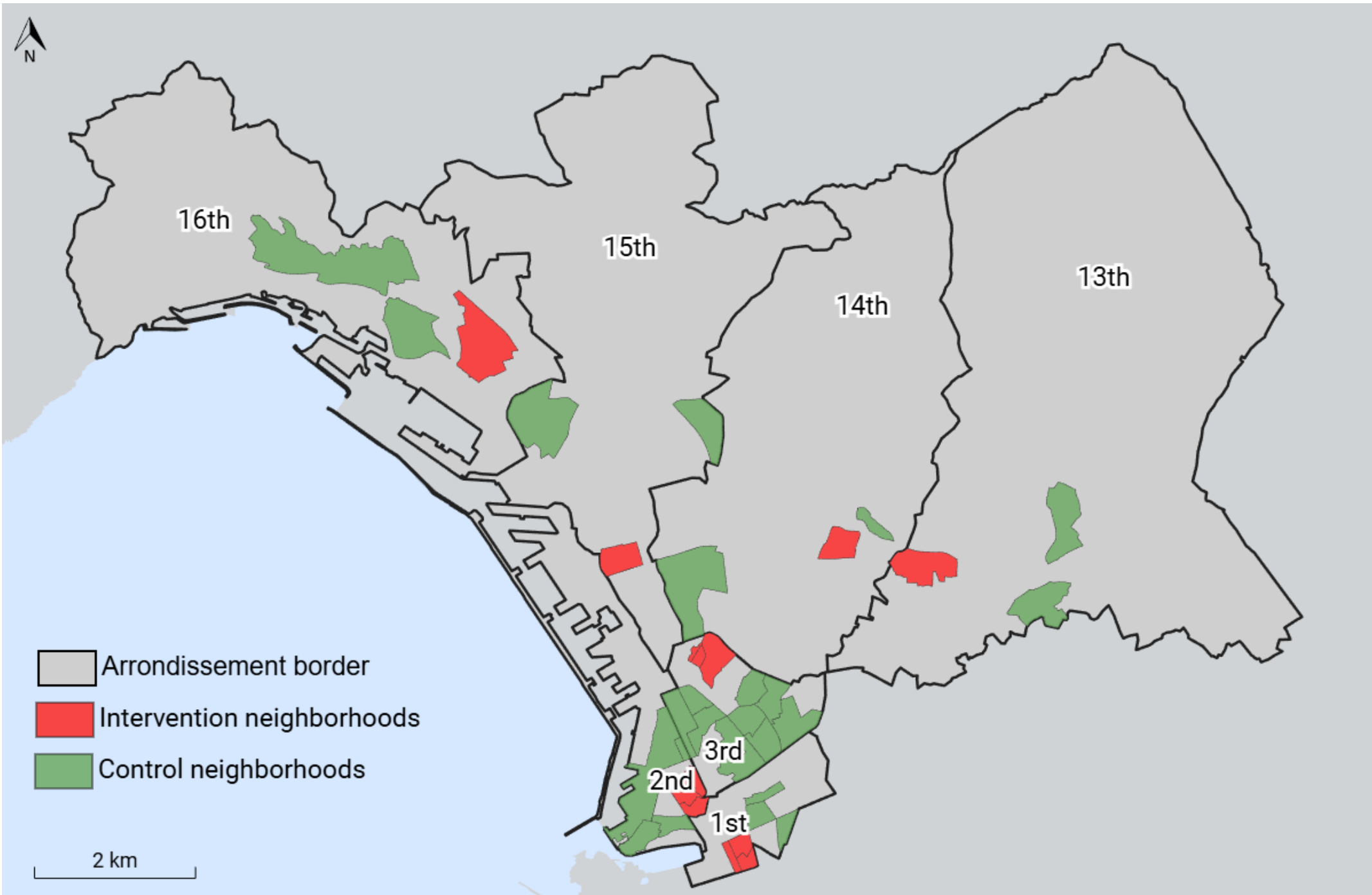

Supplementary figure 1. Intervention and control neighbourhoods included in the study, located in the city-centre (1st to 3rd arrondissement) and the Northern arrondissements of Marseille (13th to 16th arrondissements), France.

[5] Institut national de la statistique et des études économiques (INSEE). Population en 2021. 2024. https://www.insee.fr/fr/statistiques/8268806 (accessed Dec 4, 2025).

APPENDIX 2. PARTICIPANTS

Supplementary method 3. Stratification sampling method and participants' recruitment

Participants were recruited following a predefined walking rules common to each neighbourhood. The geographical starting point was randomly selected for each neighbourhood. The research assistant interviewed one eligible individual per household who met the age and sex criteria, continuing until the target number of participants for each stratum (age, sex, and neighbourhood) was reached.

APPENDIX 3. PROCEDURES

Supplementary method 4. Questionnaire knowledge-attitude-practice on cancer screening and vaccination

The French questionnaire was translated into English for publication, but the English version was not administered or tested in practice. Items related to vaccination outcomes are not included here and will be reported in a separate article. When an item was drawn from one of the two national cancer-related surveys conducted by Santé publique France (*Baromètre 2015* or *Baromètre 2021*), its source is indicated in italicized blue text in brackets[6]. When no source is specified, the item was developed for this study.

Questionnaire knowledge-attitude-practice on cancer screening and vaccination

Introduction

Hello,
My colleague and I are conducting a survey as part of a project aimed at improving the health of residents in Marseille. We are working with the CORHESAN/SEPT team to provide health information and support to the city's residents, particularly regarding screening for diseases such as certain cancers and vaccination.
Would you be willing to spare a few minutes to answer some questions that will help us better guide our actions? After completing this questionnaire, we will be available to answer any questions you may have about your health or that of your family.
This questionnaire is completely anonymous and will only take a few minutes.

Information completed by the research assistant:

Name of the organization: ☐ CORHESAN ☐ SEPT

Type of neighbourhood: ☐ Control ☐ Intervention

Name of the neighbourhood: _ _ _ _ _

General information about the respondent

To begin, I have a few general questions to ask you.

1. What is your sex? *(source: Baromètre 2021)*

- ☐ Female
- ☐ Male
- ☐ Non-binary / Other

2. How old are you? _ _ _ _ _ (years) *(source: Baromètre 2021)*

[6] Institut national du cancer, Santé publique France. Baromètre Cancer 2021. Attitudes et comportements des Français face au cancer. 2023 ; Institut national du cancer, Santé Publique France. Baromètre cancer 2015. Questionnaire.

Breast cancer screening

Questions for women aged 50 to 74

Now we are going to talk about breast cancer screening.

Knowledge

3. Have you ever heard about breast cancer screening?

☐ Yes
☐ No
☐ Prefer not to answer

If no to Q3, explain what a mammography is:
A mammography is a breast radiography exam that uses low-dose X-rays. It is performed using a device called a "mammography unit." A mammography is mainly used to screen for possible breast cancer.

Mammographie

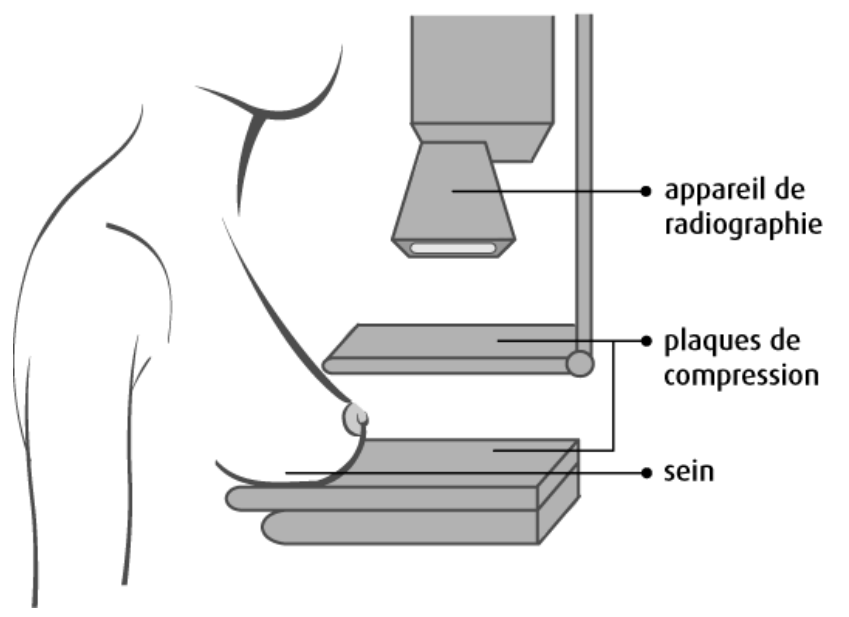

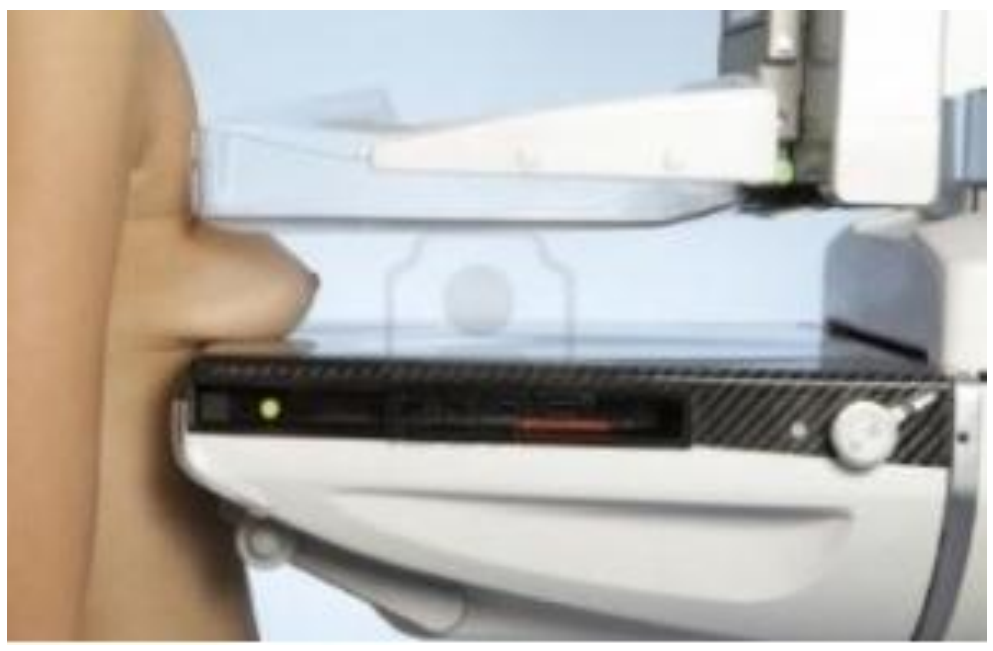

Attitudes and practice

Questions for women aged 50 to 74

4. Have you received a letter inviting you to have a mammography since turning 50? Or has your doctor talked to you about it?

☐ Yes
☐ No
☐ Don't know
☐ Prefer not to answer

5. Have you ever had a mammography?

☐ Yes
☐ No
☐ Don't know
☐ Prefer not to answer

If yes to Q5, participant has already had a mammogram:

6. How many times have you had a mammography? *(source: Baromètre 2015)*

☐ Several times
☐ Only once

7. When did you have your most recent mammography? *(source: Baromètre 2021)*

- ☐ Less than 1 year ago
- ☐ Between 1 and 2 years ago (at least 1 year but less than 2 years)
- ☐ Between 2 and 3 years ago (at least 2 years but less than 3 years)
- ☐ Between 3 and 5 years ago (at least 3 years but less than 5 years)
- ☐ 5 years or more ago
- ☐ Don't know
- ☐ Prefer not to answer

Cervical cancer screening

Questions for women aged 25 to 64

Now we are going to talk about cervical cancer screening.

Knowledge

8. Have you ever heard about cervical cancer screening?

- ☐ Yes
- ☐ No
- ☐ Prefer not to answer

If no to Q8:

In France, cervical cancer screening is carried out through a cervical smear (Pap test). This is a medical test in which cells are collected from the cervix to look for precancerous cells (for women aged 25 to 29), or to detect the presence of human papillomavirus (HPV) (for women aged 30 to 64).

Attitudes and practice

Questions for women aged 25 to 64

9. Have you received a letter inviting you to have a cervical smear since turning 25? Or has your doctor talked to you about it?

- ☐ Yes
- ☐ No
- ☐ Don't know
- ☐ Prefer not to answer

10. Have you ever had a cervical smear (Pap test)? *(source: Baromètre 2021)*

- ☐ Yes
- ☐ No
- ☐ Don't know
- ☐ Prefer not to answer

If yes to Q10, participant has already had a cervical smear (Pap test):

11. How many times have you had a smear test? *(source: Baromètre 2015)*

- ☐ Several times
- ☐ Only once

12. When did you have your most recent smear test? *(source: Baromètre 2021)*

- ☐ Less than 1 year ago
- ☐ Between 1 and 2 years ago (at least 1 year but less than 2 years)
- ☐ Between 2 and 3 years ago (at least 2 years but less than 3 years)
- ☐ Between 3 and 5 years ago (at least 3 years but less than 5 years)
- ☐ 5 years or more ago
- ☐ Don't know
- ☐ Prefer not to answer

Colorectal cancer screening

Questions for men and women aged 50 to 74

Now we are going to talk about colorectal cancer screening.

Knowledge

13. Have you ever heard about colorectal cancer screening?

- ☐ Yes
- ☐ No
- ☐ Prefer not to answer

If no to Q13:

In France, colorectal cancer screening is easy thanks to an immunological test. This test (formerly known as Hemoccult) detects hidden blood in the stool. It is quick and effective. The test is done at home and then sent free of charge to a laboratory for analysis. It is intended for men and women aged 50 to 74. For people identified as being at higher risk by a doctor, screening is carried out using a colonoscopy.

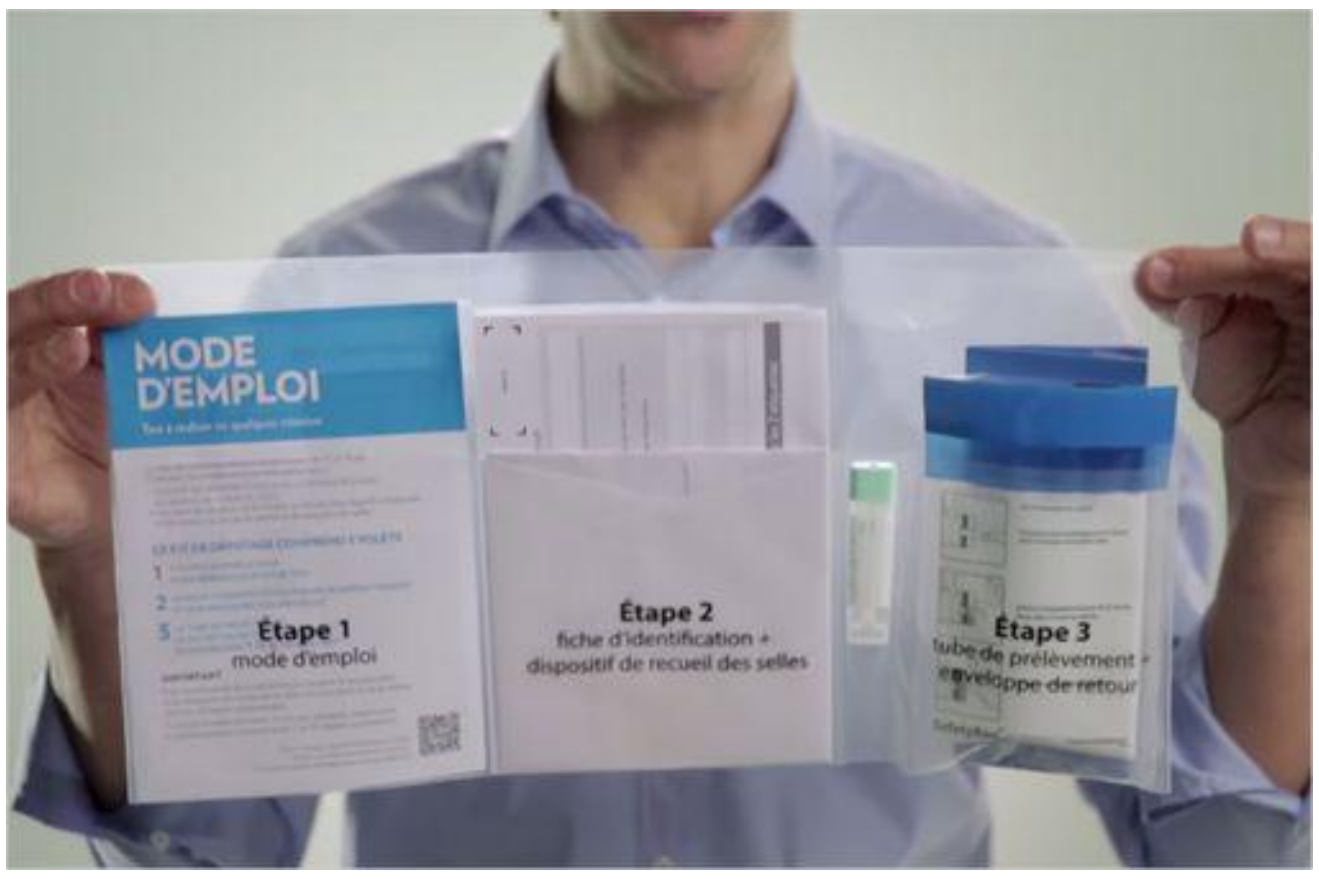

Attitudes and practice

Questions for men and women aged 50 to 74

14. Have you received a letter inviting you to do an at-home test since turning 50? Or has your doctor talked to you about it?

- ☐ Yes
- ☐ No
- ☐ Don't know
- ☐ Prefer not to answer

15. Have you ever had colorectal cancer screening using a stool blood test (immunological test) done at home or a colonoscopy? *(source: Baromètre 2015)*

- ☐ Yes
- ☐ No
- ☐ Don't know
- ☐ Prefer not to answer

If yes to Q15, participant has already had an immunological test/colonoscopy:

16. How many times have you had this test? *(source: Baromètre 2015)*

- ☐ Several times
- ☐ Only once

17. When did you have this test for the last time? *(source: Baromètre 2021)*

- ☐ Less than 1 year ago
- ☐ Between 1 and 2 years ago (at least 1 year but less than 2 years)
- ☐ Between 2 and 3 years ago (at least 2 years but less than 3 years)
- ☐ Between 3 and 5 years ago (at least 3 years but less than 5 years)
- ☐ 5 years or more ago
- ☐ Don't know
- ☐ Prefer not to answer

General information about the respondent

Questions for all respondents

We are almost finished. I just need to ask you a few details about your personal situation.

18. Do you have a primary care physician or a doctor you consult regularly? *(source: Baromètre 2021)*

- ☐ Yes
- ☐ No
- ☐ Prefer not to answer

19. Where were you born?

- ☐ In mainland France
- ☐ In an overseas French department or territory (DOM-TOM)
- ☐ Abroad
- ☐ Prefer not to answer

20. Is French your mother tongue?

- ☐ Yes
- ☐ No
- ☐ Prefer not to answer

21. Up to what level of education did you complete your studies?

- ☐ Primary school
- ☐ Middle school / Secondary school (junior and senior high school)
- ☐ High school diploma to Bac +3
- ☐ Bac +4 and above
- ☐ Other
- ☐ Prefer not to answer

22. What is your current employment situation? *(source: Baromètre 2021)*

- ☐ Employed
- ☐ Apprentice or paid internship
- ☐ Student, high school student, in training, or unpaid internship
- ☐ Unemployed (registered or not with the employment agency)
- ☐ Retired or pre-retired
- ☐ Homemaker
- ☐ Other situation (long-term leave, disability, etc.)
- ☐ Don't know
- ☐ Prefer not to answer

23. Currently, regarding your housing situation:

- ☐ Homeowner
- ☐ Tenant
- ☐ Living with friends
- ☐ Living with parents or other family members
- ☐ Living in a hotel, hostel, shelter, or accommodation centre
- ☐ Prefer not to answer

Participation to a health mediation intervention

Questions for all respondents only at T1 survey

24. Since September 2022, have you met any health mediators from the SEPT or CORHESAN associations with whom you discussed cancer screening and vaccination?

- ☐ Yes
- ☐ No
- ☐ Don't know
- ☐ Prefer not to answer

Thank you very much for your participation in this survey.

Supplementary table 1. Variables' description and construction

Variable	Modalities
**Primary outcomes: cancer screening uptake according to national guidelines**	
Breast cancer	▪ Up to date: screening performed within the last two years.<br>▪ Not up to date: never screened or last screening more than two years ago.
Colorectal cancer	▪ Up to date: screening performed within the last two years.<br>▪ Not up to date: never screened or last screening more than two years ago.
Cervical cancer	▪ Up to date: screening performed within the past 1 year for women aged 25–26, within the past 3 years for women aged 27–30, and within the past 5 years for women aged 30–64.<br>▪ Not up to date: never screened or last screening performed outside the recommended interval for the corresponding age group.
**Secondary outcomes: cancer screening awareness**	
Breast cancer	▪ Aware: has ever heard of breast cancer screening.<br>▪ Not aware: never heard of breast cancer screening.
Colorectal cancer	▪ Aware: has ever heard of colorectal cancer screening.<br>▪ Not aware: never heard of colorectal cancer screening.
Cervical cancer	▪ Aware: has ever heard of breast cancer screening.<br>▪ Not aware: never heard of cervical cancer screening.
**Health mediation**	
Encounter with a health mediator	▪ Reported: has met health mediators from the SEPT or CORHESAN since September 2022 to discuss cancer screening and vaccination.<br>▪ Not reported: has not met health mediators from the SEPT or CORHESAN since September 2022 to discuss cancer screening and vaccination.
**Access to healthcare system**	
Primary care physician	▪ With: has a regular primary care physician<br>▪ Without: does not have a regular primary care physician
Breast cancer screening invitation	▪ Received: has been invited by her physician or by a letter from the National Health Insurance<br>▪ Not received: has not been invited by her physician or by a letter from the National Health Insurance
Colorectal cancer screening invitation	▪ Received: has been invited by her physician or by a letter from the National Health Insurance<br>▪ Not received: has not been invited by her physician or by a letter from the National Health Insurance
Cervical cancer screening invitation	▪ Received: has been invited by her physician or by a letter from the National Health Insurance<br>▪ Not received: has not been invited by her physician or by a letter from the National Health Insurance
**Socioeconomic characteristics**	
Birthplace	▪ France: born in France ("Overseas Departments and Territories" and "Metropolitan France" combined due to low number of observations).<br>▪ Abroad: born abroad.
Mother tongue	▪ French: French mother tongue<br>▪ Other: non-French mother tongue
Education level	▪ Primary school: first stage of compulsory education, including preschool and elementary school for children aged 3 to 11 or equivalent<br>▪ Secondary school: second stage of compulsory education for children aged 12 to 18, including high school or equivalent.

	▪ Undergraduate: first level of higher education, pursuing a bachelor's degree or equivalent.<br>▪ Postgraduate: advanced studies, such as a master's, doctoral (PhD), or professional postgraduate qualification.<br>▪ Other: all other form of education.
Occupational status	▪ Employed: currently working for pay or profit, either full-time, part-time, or self-employed.<br>▪ Student: enrolled in an educational program, combined with paid apprentice/intern due to the low number of observations (similarity validated by multiple correspondence analysis).<br>▪ Homemaker: manages and cares for their household full-time, without formal employment.<br>▪ Retired<br>▪ Unemployed: not currently working for pay or profit.<br>▪ Other: all other form of occupational status.
Residential status	▪ Homeowner: individual who owns the property they live in.<br>▪ Renter: individual who rent the property they live in.<br>▪ Family: individual living in the family household.<br>▪ Other: combined 'Friend' and 'squat/shelter' with all other form of residential status due to low number of observations
Socioeconomic profile	Profiles constructed using a multiple correspondence analysis based on socioeconomic characteristics, followed by hierarchical agglomerative clustering. Four profiles were identified and labelled based on their predominant characteristics:<br>▪ Native speaker homeowner (NS homeowner)<br>▪ Native speaker renter (NS renter)<br>▪ Native speaker student (NS student)<br>▪ Non-native speaker renter (Non-NS renter)<br>See pages 9 to 14 for more details.
**Demographic characteristics**	
Gender	Self-reported gender: female or male. Missing data corresponded to a participant declaring undifferentiated gender, but due to low total headcount and statistical limit, we couldn't consider this category in our analysis
Age	Self-reported age was collected as a quantitative variable and then discretized into 5-year intervals.
Arrondissement	District of the residential neighbourhood.

RESULTS

APPENDIX 4. SOCIOECONOMIC PROFILES CONSTRUCTION AND DESCRIPTION

4.1. Socioeconomic profiles construction

The distribution of individuals on the first two axes of the multiple correspondence analysis, which together accounted for 25.97% of the total variance (15.36% for the first axis and 10.61% for the second), showed clear discrimination according to mother tongue (French vs other), place of birth (France vs abroad) — two highly correlated variables — as well as residential and occupational status. Living in the family home was strongly associated with being a student.

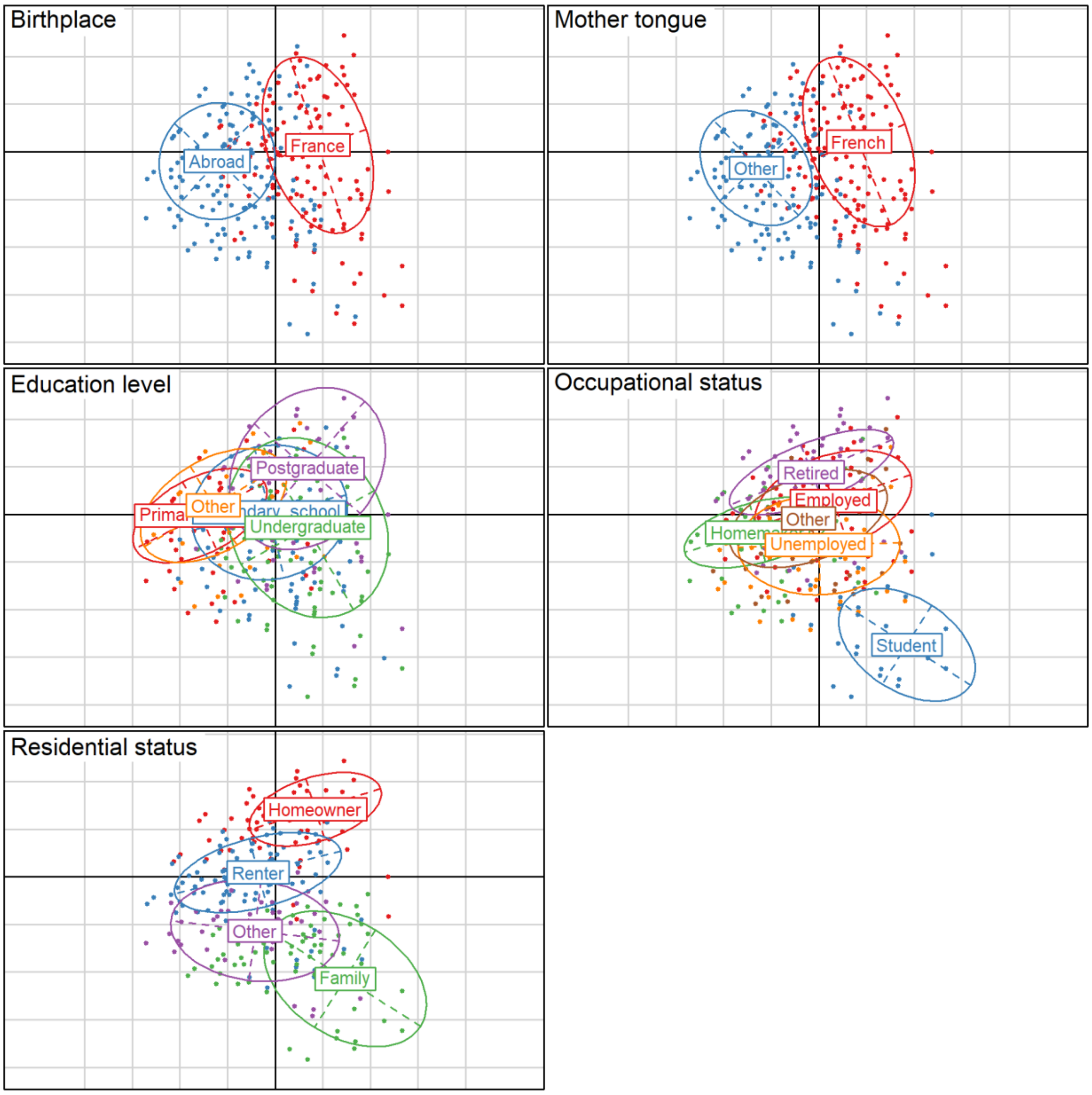

Supplementary figure 2. Distribution of individuals on the first two axes of the multiple correspondence analysis by variable categories.

A multiple correspondence analysis was performed on socioeconomic characteristics, followed by hierarchical agglomerative clustering to identify major socioeconomic profiles. Each cluster represents a group of individuals who are as similar as possible in terms of their socioeconomic characteristics while being distinct from other groups. These clusters provide a statistical approximation of social patterns within the population and are useful for identifying groups accumulating multiple social disadvantages, although some heterogeneity within clusters inevitably remains.

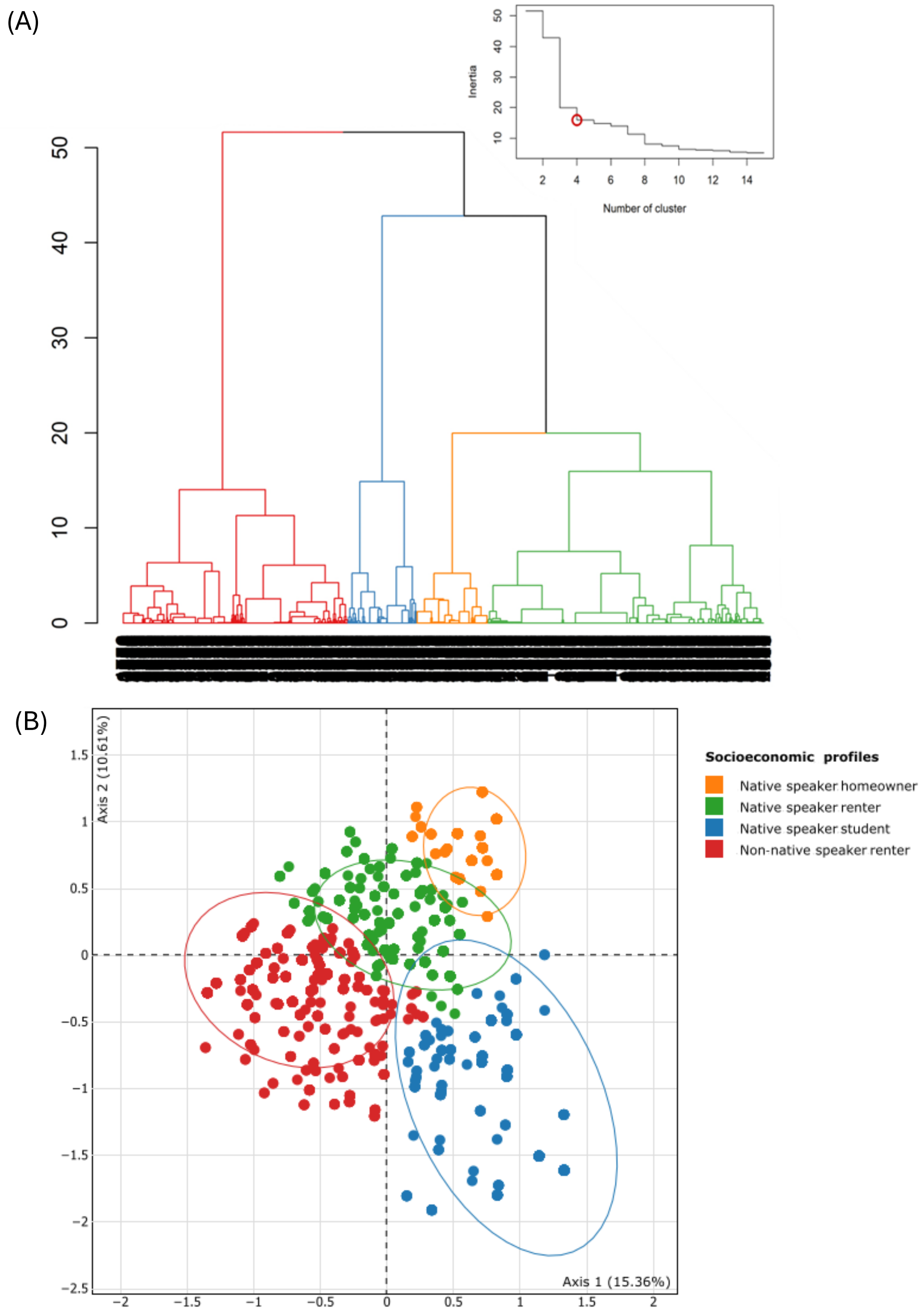

Supplementary figure 3. Hierarchical agglomerative clustering after multiple correspondence analysis. (A) Dendrogram and inertia. (B) Distribution of individuals on the first two axes of the multiple correspondence analysis by the four socioeconomic profiles identified by the clustering.

4.2. Socioeconomic profiles description before imputation

Four socioeconomic profiles were identified and named according to the most discriminating characteristics within each group: native speaker (NS) homeowner (n=483; 11.14%), native speaker (NS) renter (n=1,862; 42.96%), native speaker (NS) student (n=467; 10.78%), and non-native speaker (non-NS) renter (n=1,522; 35.12%). A total of 189 individuals out of 4,523 participants (4.18%) had missing socioeconomic profiles.

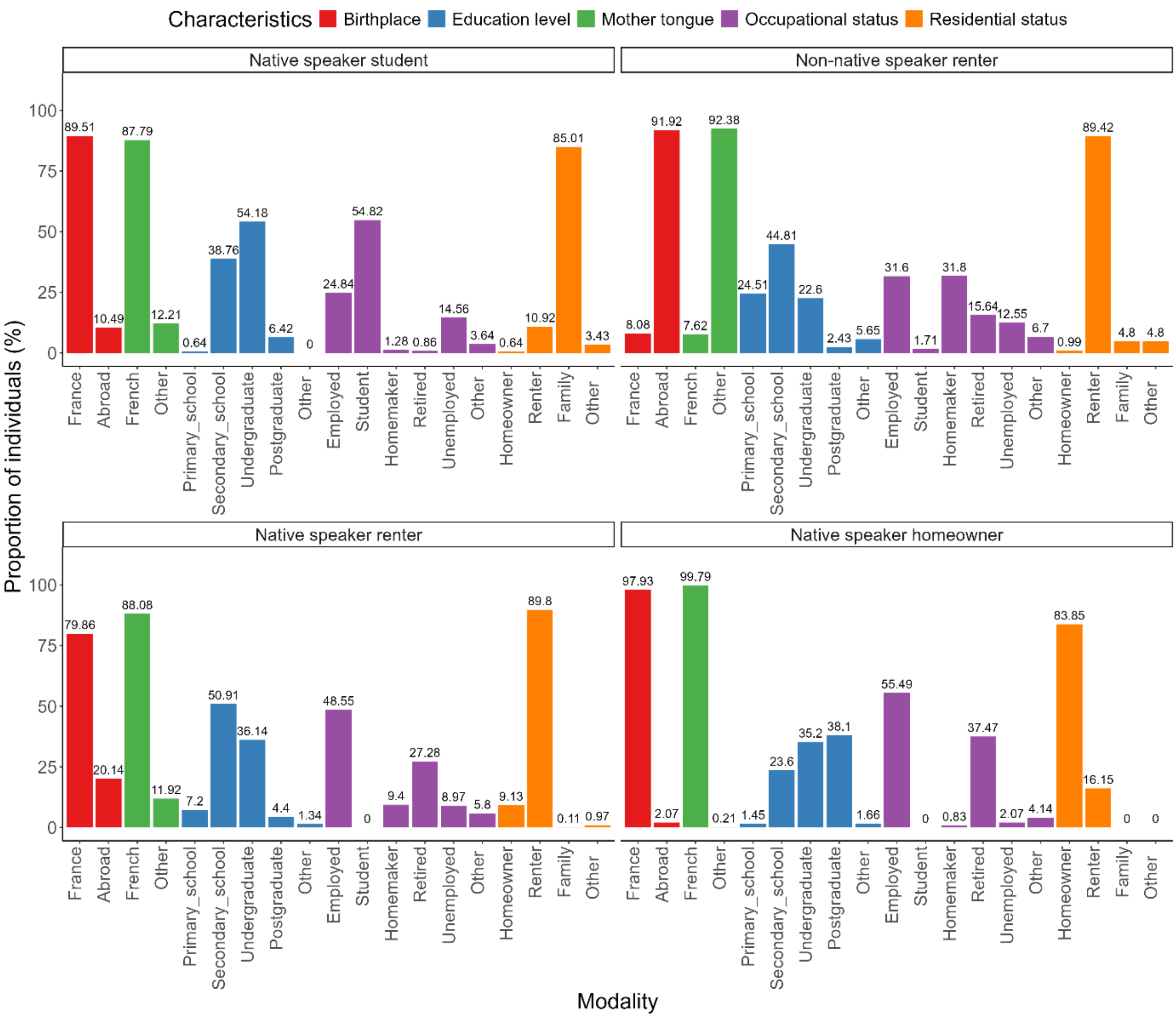

Supplementary figure 4. Socioeconomic characteristics of participants in the four profiles identified by clustering before missing data imputation.

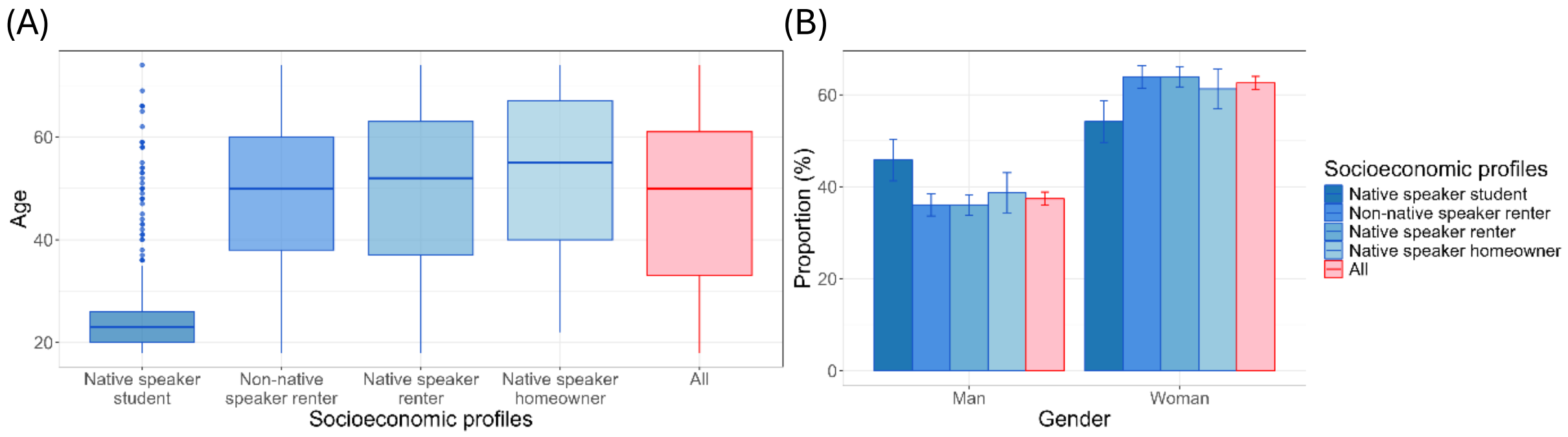

Supplementary figure 5. (A) Age and (B) gender distribution across the four socioeconomic profiles.

Among the four profiles, the “non-NS renter” profile was distinct from the others, as it included 92% of participants born abroad and without French as their mother tongue. The other three groups mostly comprised French native speakers born in France. This profile was also characterized by a high proportion of renters (90%) and the highest proportion of individuals with only primary education (25% vs <8% in the other groups). It also included the highest proportion of homemakers, comprising 32% of individuals.

The “NS student” profile was characterized by 55% students and 85% of participants living with their family. Educational attainment ranged from secondary to undergraduate level, with the highest proportion of undergraduates. Individuals in this profile had a substantially lower median age compared with the other groups (23 years vs 50–55 years), suggesting that many were likely still in education, which may explain the lower proportion of postgraduates. The proportion of men in this group was also higher than in the overall population (46% vs 37%).

The “NS homeowner” profile was characterized by the highest proportion of homeowners, comprising 84% of the group compared with <10% in the other profiles, and the highest proportion of postgraduates (38% vs <7% in the other groups). This profile also had the highest median age (55 years), with over 20% of participants aged 70–74 years (21.74% vs <11% in the other groups), resulting in the highest proportion of retirees. It also included one of the highest proportions of employees (55%).

Finally, the “NS renter” profile, representing 43% of the surveyed population, included French native speakers, 90% of whom were renters. This group also had one of the highest proportions of employees (49%).

4.3. Socioeconomic profile imputation for missing data

Overall, at least one socioeconomic variable (birthplace, education level, occupational status, mother tongue, or residential status) was missing for 189 (4.18%) of 4,523 participants. Occupational status accounted for the highest proportion of missing data (n=94; 2.08%; table 1). Socioeconomic cluster imputation was applied to these 189 participants. Imputation was performed using predicted missing clusters derived from a regression tree constructed with individuals who had complete socioeconomic information, from which their socioeconomic cluster could be assigned. Supplementary figure 6 displayed the regression tree. According to the regression tree, when no socioeconomic information was available, the cluster “NS renter” was imputed, as it was the most frequent in the study population (n=1862; 42.96%). However, because

this cluster represented only 43% of the population, the probability of misclassification was approximately 67%. Therefore, when no data were available, no cluster was assigned.

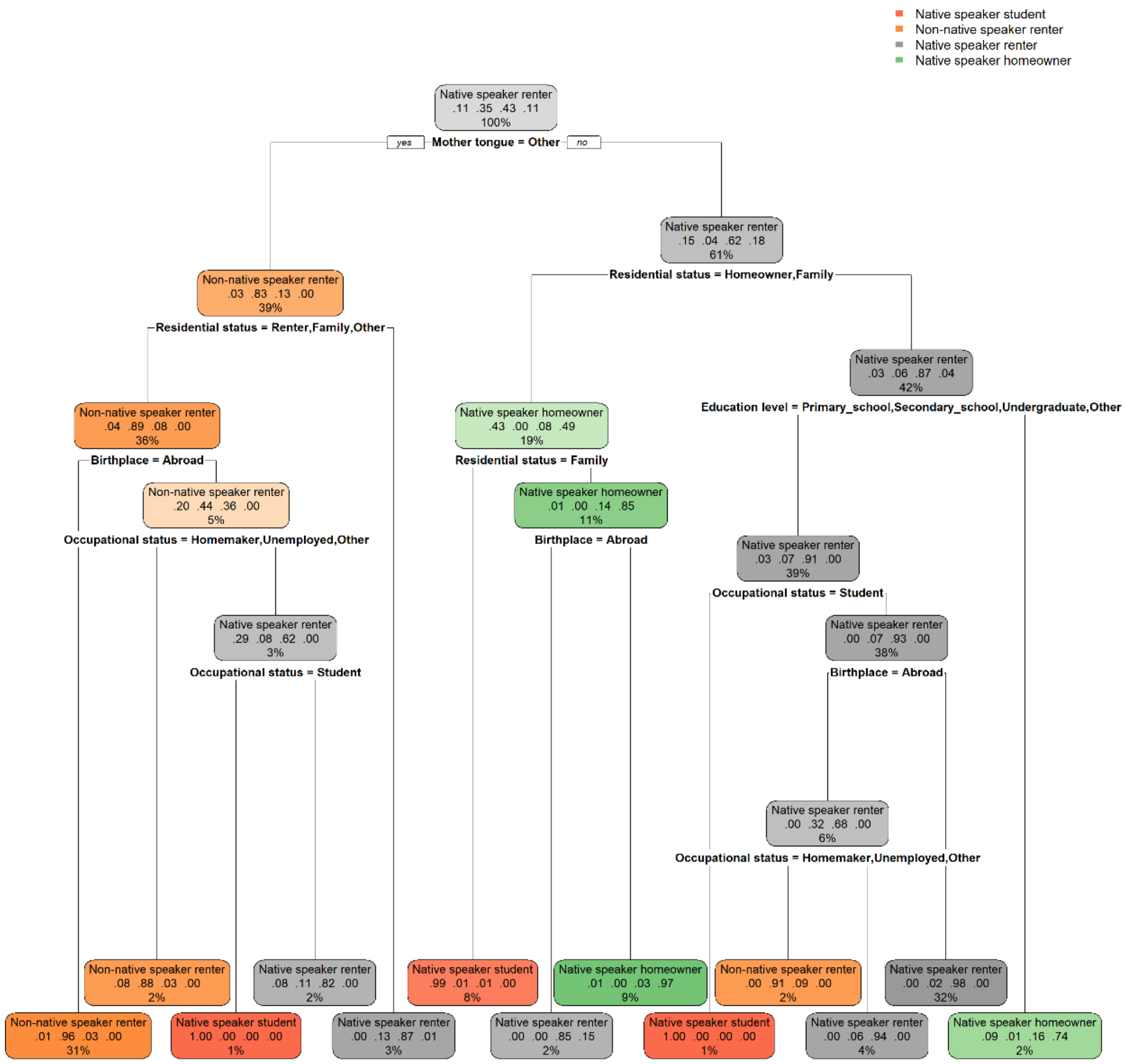

Supplementary figure 6. Regression tree of profiles based on socioeconomic variables (n=4,334).
Each node of the regression tree is represented by a rectangle. Within each rectangle, the proportion of the total population in the node and its distribution across the four socioeconomic clusters (native speaker (NS) student, non-native speaker (non-NS) renter, native speaker (NS) renter, and native speaker (NS) homeowner, in this order) are displayed. The socioeconomic cluster labelling the node corresponds to the class assigned. For example, the first node of the tree is labelled "native speaker renter." When no socioeconomic information was available for a participant, the class "native speaker renter" was imputed, as it was the most frequent class in the study population (n=1,862, 42.96%). The variable used to partition the sample is shown below each node. For example, the first partition was based on the variable "mother tongue," with the category "Other" appearing after the "=". On the left side of the tree, participants with a mother tongue other than French are grouped, whereas on the right side, participants with French as their mother tongue are grouped.
In the second right node, participants with French as their mother tongue accounted for 61% (2,648 of 4,334 participants) of the population. In the absence of additional socioeconomic information, the "native speaker renter" cluster was assigned, as it was the most frequent class in this subgroup (1,640 of 2,648 participants, 61.93%). This group was then divided according to residential status. Participants who reported being home owner or living in family home were grouped in the left node and all assigned to the "native speaker student" cluster, whereas those who did not report being students were grouped in the right node.

4.4. Socioeconomic profiles description after imputation

The four socioeconomic profiles remained unchanged after missing data imputation, and their distributions were largely stable: NS homeowner (n=493, 10.90% vs n=483, 11.14% before imputation), NS renter (n=1,938, 42.85% vs n=1,862, 42.96% before imputation), NS student (n=477, 10.55% vs n=467, 10.78% before imputation), and non-NS renter (n=1,608, 35.55% vs n=1,522, 35.12% before imputation). Seven individuals (0.15%) still had missing socioeconomic profiles because all socioeconomic variables were missing.

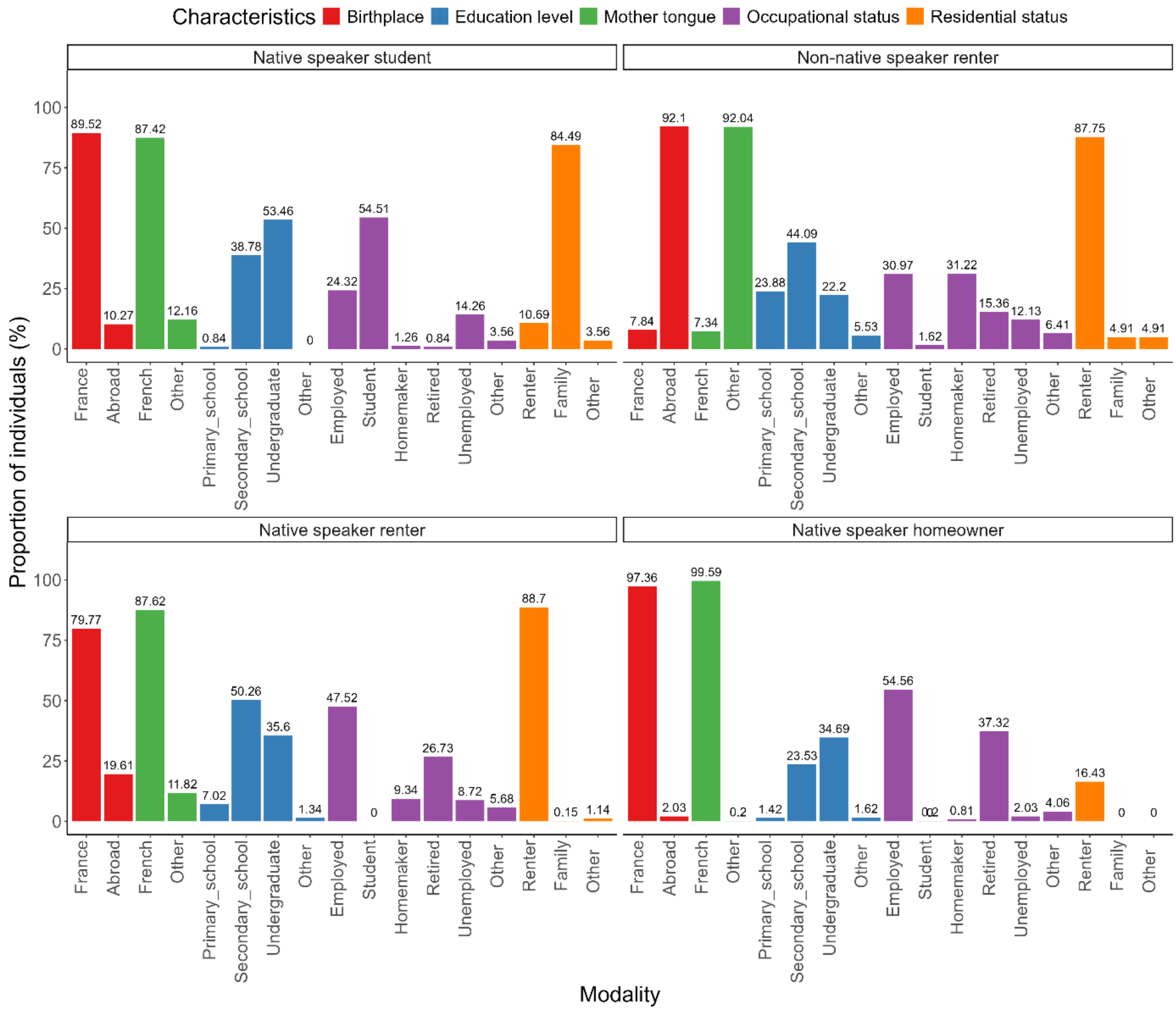

Supplementary figure 7. Socioeconomic characteristics of participants in the four profiles identified by clustering after missing data imputation.

APPENDIX 5. IMBALANCE BETWEEN GENERAL POPULATION AND THE SAMPLE SURVEYED AND WEIGHTING

Because of the sampling design, women and individuals aged 65-74 years were overrepresented in the surveyed sample (Supplementary figure 8). To adjust the sample to the structure of the general population by age and sex, higher weights were assigned to men and to younger age groups (Supplementary figure 9). This weighting procedure reduced discrepancies with the general population and produced a sample structure representative of the general population in terms of age and sex (Supplementary figure 10).

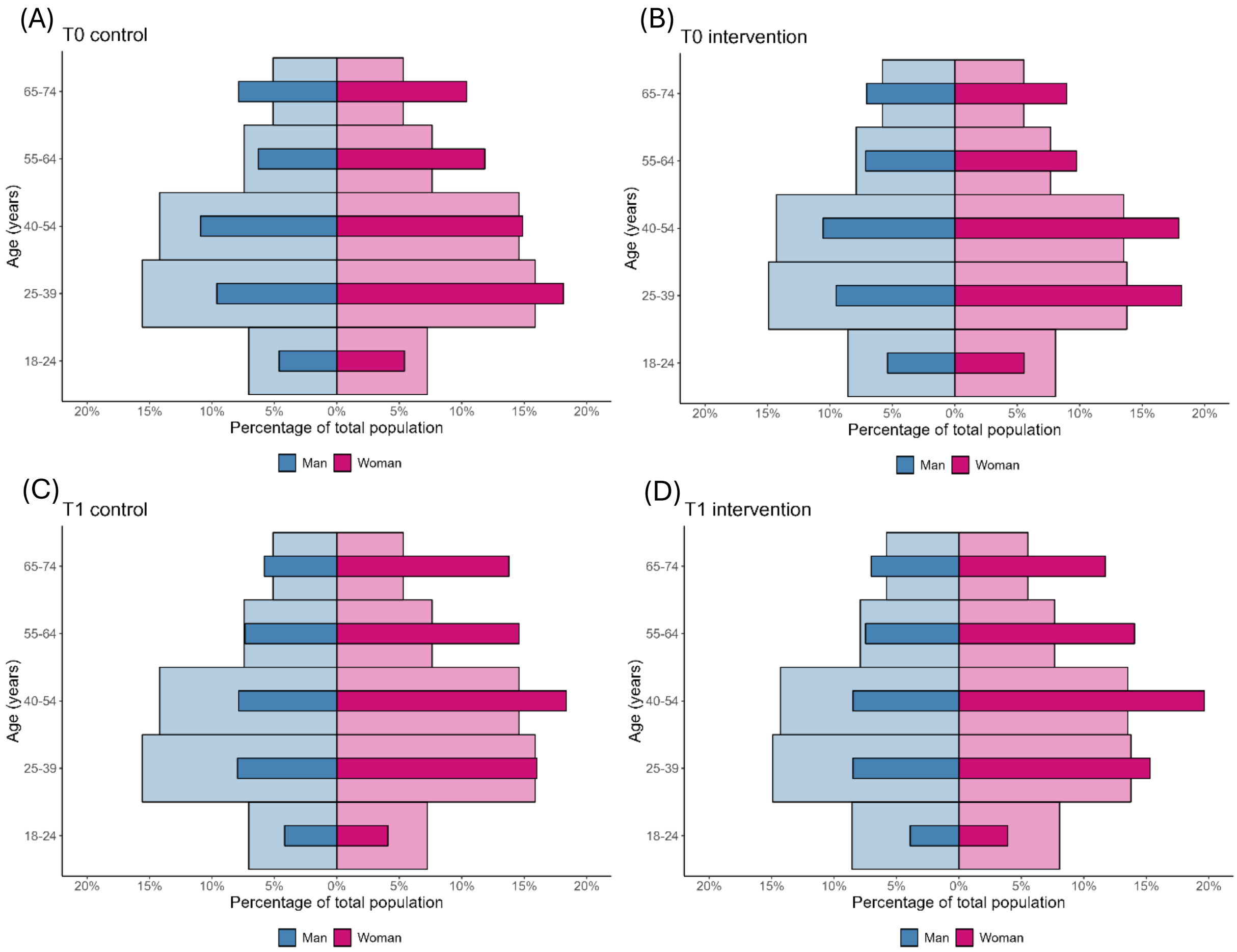

Supplementary figure 8. Comparison of age pyramids for the general population (wide light-coloured bars) and the survey samples (narrow dark-coloured bars) in (A) T0 control, (B) T0 intervention, (C) T1 control, and (D) T1 intervention neighbourhoods.

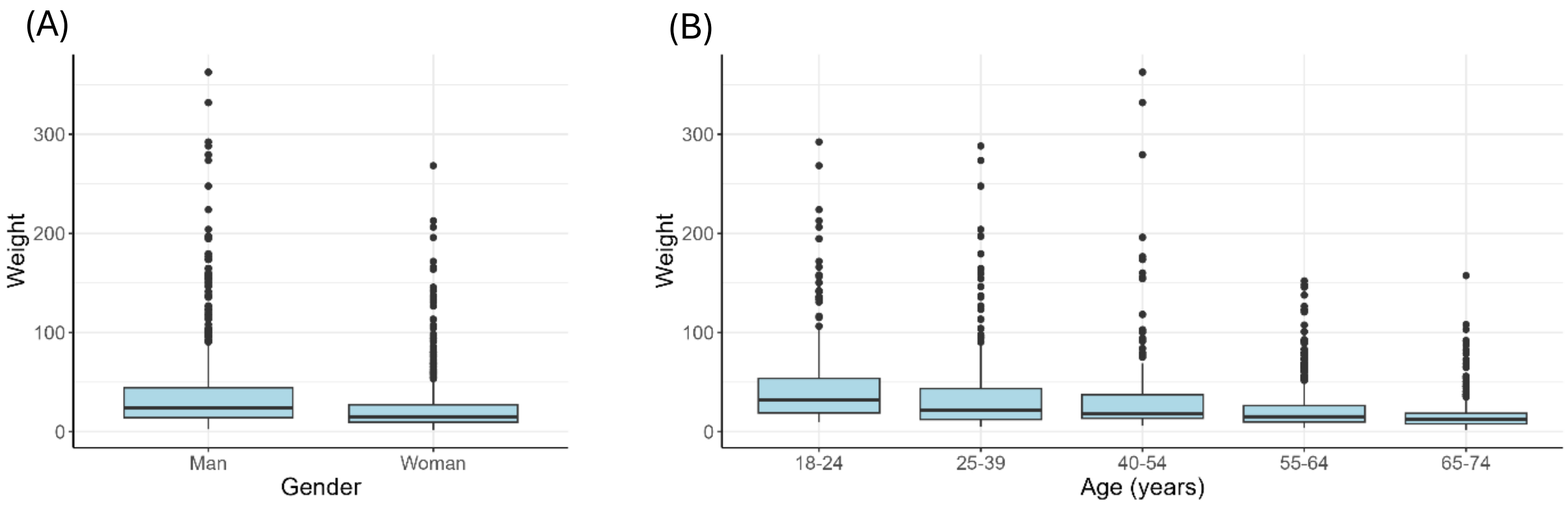

Supplementary figure 9. Weight distribution of the surveyed sample according to (A) gender and (B) age.

Although socioeconomic profiles were not directly included in the weighting process, this adjustment also affected their distribution within the sample. Specifically, it increased the relative weight of younger participants, particularly students, as observed in Supplementary figure 5 (Supplementary figure 11). A significant difference remained between the proportions of the "non-NS renter" profile at T0 and T1 in intervention neighbourhoods (T0, 41.51% [38.31–44.78] vs T1, 31.19% [27.71–34.90]). The socioeconomic profile should therefore be considered as a potential confounding factor in the effectiveness models.

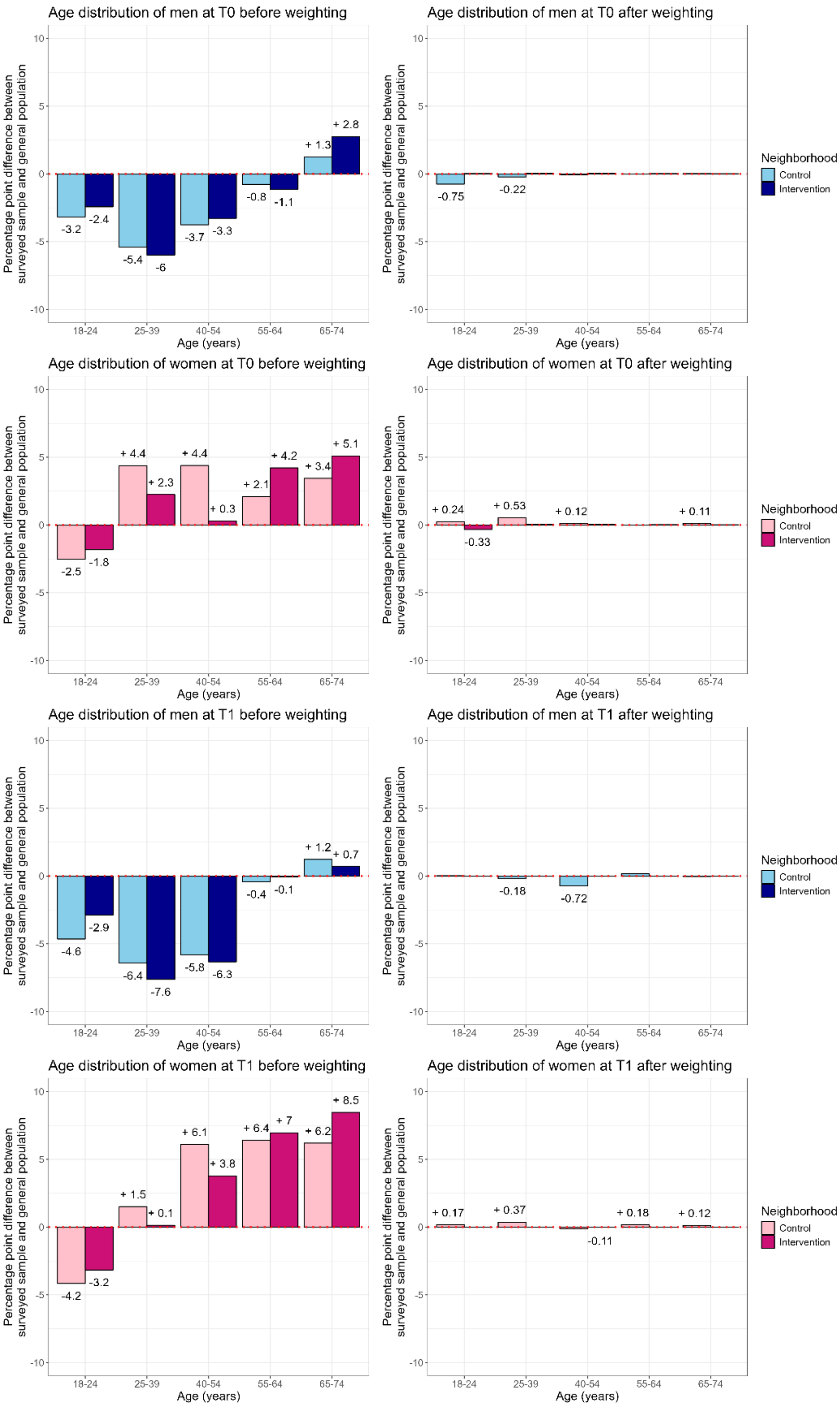

Supplementary figure 10. Percentage point difference of age and gender distributions between surveyed sample and general population, according to neighbourhood type and survey time, before and after weighting.

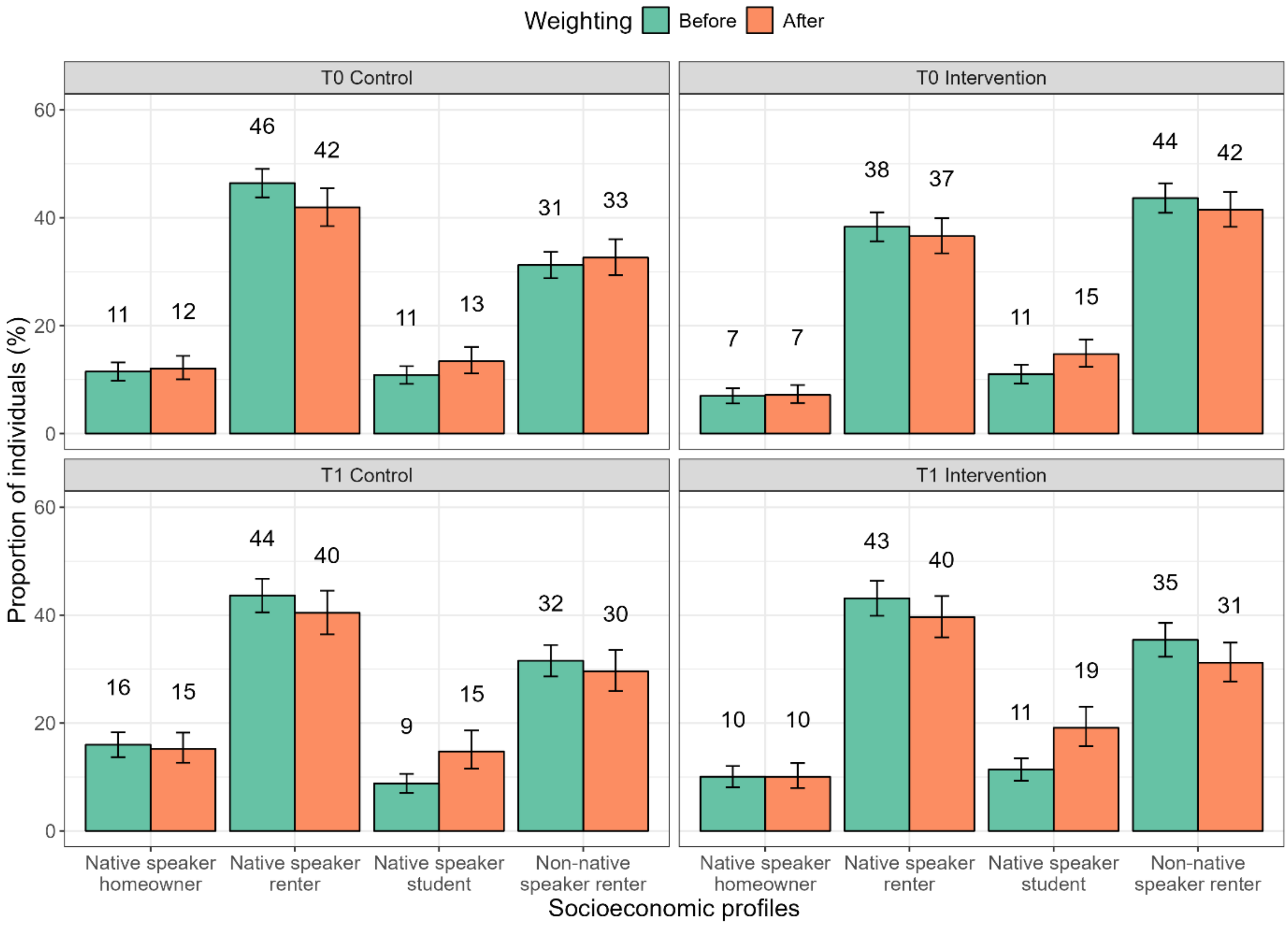

Supplementary figure 11. Proportion of socioeconomic profiles in the surveyed sample before and after weighting, according to neighbourhood type and survey time.

APPENDIX 6. ENCOUNTER WITH A HEALTH MEDIATOR

Supplementary table 2. Factors associated with individuals reporting an encounter with a health mediator during the T1 survey.

	n (%)	n with encounter (weighted %)	Univariate analysis Odds ratio (95% CI)	p value	Multivariate analysis Odds ratio (95% CI)	p value
**Encounter with a health mediator** (n [without missing outcome] = 1,805; n [multivariable analysis] = 1,764)						
**Neighbourhoods by survey phase**						
T1 P1 Control	851 (47.15)	42 (3.80)	1 (ref)	..	1 (ref)	..
T1 P1 Intervention	571 (31.63)	96 (16.62)	5.05 (2.89-8.82)	<0.0001	5.43 (3.10-9.51)	<0.0001
T1 P2 Control	79 (4.38)	2 (2.26)	0.59 (0.12-2.93)	0.5149	0.72 (0.15-3.46)	0.68
T1 P2 Intervention	265 (14.68)	14 (3.65)	0.96 (0.43-2.12)	0.9160	1.31 (0.50-3.40)	0.58
Missing data	39 (2.16)	9 (17.93)	..	..	..	..
**Primary care physician**						
Without	131 (7.26)	7 (3.12)	1 (ref)	..	1 (ref)	..
With	1,672 (92.63)	155 (6.59)	2.19 (0.83-5.80)	0.11	2.12 (0.71-6.30)	0.18
Missing data	2 (0.11)	1 (53.29)	..	..	..	..
**Gender**						
Man	618 (34.24)	51 (6.22)	1 (ref)	..	1 (ref)	..
Woman	1,187 (65.76)	112 (6.31)	1.02 (0.62-1.66)	0.95	0.96 (0.55-1.67)	0.87
**Age**						
18-24	149 (8.25)	11 (5.81)	1 (ref)	..	1 (ref)	..
25-29	180 (9.97)	9 (3.10)	0.52 (0.18-1.49)	0.22	0.60 (0.22-1.63)	0.32
30-34	129 (7.15)	8 (3.48)	0.58 (0.21-1.65)	0.31	1.02 (0.35-2.92)	0.98
35-39	131 (7.26)	13 (9.04)	1.61 (0.48-5.45)	0.44	2.74 (0.75-9.94)	0.13
40-44	102 (5.65)	14 (6.64)	1.15 (0.44-3.02)	0.77	2.19 (0.77-6.24)	0.14
45-49	115 (6.37)	12 (8.17)	1.44 (0.56-3.73)	0.45	2.01 (0.72-5.65)	0.18
50-54	267 (14.79)	28 (8.07)	1.42 (0.58-3.47)	0.44	2.01 (0.70-5.77)	0.20
55-59	174 (9.64)	18 (6.22)	1.08 (0.45-2.59)	0.87	1.95 (0.74-5.17)	0.18
60-64	217 (12.02)	22 (7.73)	1.36 (0.55-3.33)	0.50	2.10 (0.74-5.93)	0.16
65-69	170 (9.42)	16 (5.79)	1.00 (0.40-2.46)	0.99	1.66 (0.62-4.42)	0.31
70-74	171 (9.47)	12 (5.58)	0.96 (0.36-2.53)	0.93	1.52 (0.47-4.96)	0.48
**Socioeconomic profile**						
NS renter	784 (43.43)	44 (3.68)	1 (ref)	..	1 (ref)	..
NS homeowner	238 (13.19)	15 (4.18)	1.14 (0.54-2.41)	0.73	1.54 (0.69-3.44)	0.29
NS student	186 (10.30)	15 (6.26)	1.75 (0.85-3.59)	0.13	2.80 (1.25-6.27)	0.01
Non-NS renter	596 (33.02)	88 (10.81)	3.18 (1.84-5.49)	<0.0001	2.84 (1.63-4.96)	0.0002
Missing data	1 (0.06)	1 (100.00)	..	..	..	..
**Arrondissement**						
1st	226 (12.52)	24 (5.07)	1 (ref)	..	1 (ref)	..
2nd	215 (11.91)	18 (4.95)	0.98 (0.44-2.15)	0.95	0.98 (0.42-2.30)	0.96
3rd	470 (26.04)	60 (6.81)	1.37 (0.69-2.72)	0.37	1.59 (0.76-3.30)	0.22
13th	301 (16.68)	34 (11.11)	2.34 (1.20-4.57)	0.01	1.61 (0.75-3.45)	0.22
14th	148 (8.20)	15 (10.88)	2.29 (1.01-5.16)	0.05	1.55 (0.62-3.87)	0.35
15th	251 (13.91)	6 (3.21)	0.62 (0.22-1.75)	0.37	1.00 (0.29-3.40)	1
16th	194 (10.75)	6 (2.40)	0.46 (0.17-1.26)	0.13	0.30 (0.10-0.89)	0.03

APPENDIX 7. CANCER SCREENING UPTAKE

7.1. Breast cancer screening uptake

Supplementary table 3. Factors associated with up-to-date breast cancer screening.

	n (%)	n up to date (weighted %)	Univariate analysis Odds ratio (95% CI)	p value	Multivariate analysis Odds ratio (95% CI)	p value
**Breast cancer screening uptake** (women aged 50–74 years, n [without missing outcome] = 1,389, n [multivariate analysis] = 1,252)						
**Neighbourhoods by survey phase**						
T0 Control	379 (27.29)	191 (52.82)	1 (ref)	..	1 (ref)	..
T0 Intervention	321 (23.11)	181 (57.66)	1.22 (0.86-1.72)	0.28	1.43 (0.97-2.13)	0.07
T1 P1 Control	340 (24.48)	217 (63.32)	1.54 (1.06-2.23)	0.02	1.37 (0.90-2.07)	0.14
T1 P1 Intervention	182 (13.10)	96 (54.42)	1.07 (0.71-1.59)	0.75	0.90 (0.56-1.45)	0.67
T1 P2 Control	29 (2.09)	5 (24.47)	0.29 (0.10-0.81)	0.02	0.27 (0.10-0.76)	0.01
T1 P2 Intervention	126 (9.07)	41 (29.31)	0.37 (0.23-0.59)	<0.0001	0.28 (0.16-0.49)	<0.0001
Missing data	12 (0.86)	7 (70.46)	..	..	..	..
**Encounter with a health mediator**						
Not reported	1,292 (93.02)	679 (53.76)	1 (ref)	..	1 (ref)	..
Reported	61 (4.39)	42 (73.62)	2.40 (1.31-4.41)	0.0048	2.26 (1.12-4.54)	0.02
Missing data	36 (2.59)	17 (58.01)	..	..	..	..
**Primary care physician**						
Without	65 (4.68)	17 (29.75)	1 (ref)	..	1 (ref)	..
With	1,322 (95.18)	721 (55.96)	3.00 (1.41-6.40)	0.0045	3.90 (1.76-8.65)	0.0008
Missing data	2 (0.14)	0 (0.00)	..	..	..	..
**Screening invitation**						
Not received	187 (13.46)	74 (38.81)	1 (ref)	..	1 (ref)	..
Received	1,115 (80.27)	646 (58.83)	2.25 (1.50-3.39)	0.0001	2.52 (1.67-3.78)	<0.0001
Missing data	87 (6.26)	18 (20.18)	..	..	..	..
**Age**						
50-54	377 (27.14)	226 (63.20)	1 (ref)	..	1 (ref)	..
55-59	277 (19.94)	158 (53.65)	0.67 (0.44-1.03)	0.07	0.51 (0.31-0.83)	0.0064
60-64	264 (19.01)	129 (49.65)	0.57 (0.38-0.86)	0.0068	0.50 (0.32-0.78)	0.0022
65-69	221 (15.91)	122 (55.59)	0.73 (0.48-1.11)	0.14	0.62 (0.38-1.01)	0.05
70-74	250 (18.00)	103 (39.36)	0.38 (0.25-0.57)	<0.0001	0.30 (0.19-0.49)	<0.0001
**Socioeconomic profile**						
NS renter	659 (47.44)	361 (53.30)	1 (ref)	..	1 (ref)	..
NS homeowner	205 (14.76)	116 (57.02)	1.16 (0.78-1.74)	0.46	1.17 (0.74-1.84)	0.50
Non-NS renter	514 (37.01)	258 (54.72)	1.06 (0.78-1.44)	0.72	1.02 (0.72-1.46)	0.90
Missing data[1]	11 (0.79)	3 (57.23)	..	..	..	..
**Arrondissement**						
1st	147 (10.58)	79 (63.47)	1 (ref)	..	1 (ref)	..
2nd	182 (13.10)	83 (43.23)	0.44 (0.24-0.80)	0.0068	0.58 (0.29-1.18)	0.13
3rd	369 (26.57)	201 (55.33)	0.71 (0.42-1.21)	0.21	0.67 (0.36-1.24)	0.20
13th	214 (15.41)	117 (55.65)	0.72 (0.42-1.25)	0.24	0.77 (0.41-1.44)	0.41
14th	117 (8.42)	58 (49.08)	0.55 (0.30-1.03)	0.06	0.54 (0.27-1.10)	0.09
15th	211 (15.19)	101 (49.18)	0.56 (0.32-0.96)	0.04	0.78 (0.41-1.47)	0.44
16th	149 (10.73)	99 (67.31)	1.19 (0.66-2.13)	0.57	1.10 (0.57-2.14)	0.77

[1], Native speaker student considered as missing data because of to low frequence

7.2. Colorectal cancer screening uptake

Supplementary table 4. Factors associated with up-to-date colorectal cancer screening.

	n (%)	n up to date (weighted %)	Univariate analysis Odds ratio (95% CI)	p value	Multivariate analysis Odds ratio (95% CI)	p value
**Colorectal cancer screening uptake** (man and women aged 50–74 years, n [without missing outcome] = 2,206, n [multivariate analysis] = 1,925)						
**Neighbourhoods by survey phase**						
T0 Control	632 (28.65)	209 (29.33)	1 (ref)	..	1 (ref)	..
T0 Intervention	545 (24.71)	170 (29.34)	1.00 (0.74-1.36)	1	1.03 (0.72-1.47)	0.89
T1 P1 Control	486 (22.03)	163 (33.93)	1.24 (0.88-1.75)	0.23	1.14 (0.78-1.67)	0.50
T1 P1 Intervention	304 (13.78)	84 (31.22)	1.09 (0.76-1.58)	0.63	0.91 (0.59-1.39)	0.65
T1 P2 Control	45 (2.04)	5 (11.98)	0.33 (0.12-0.92)	0.03	0.39 (0.12-1.21)	0.10
T1 P2 Intervention	172 (7.80)	39 (21.61)	0.66 (0.42-1.05)	0.08	0.69 (0.40-1.20)	0.19
Missing data	22 (1.00)	8 (42.49)	..	..	..	..
**Encounter with a health mediator**						
Not reported	2,062 (93.47)	626 (29.55)	1 (ref)	..	1 (ref)	..
Reported	93 (4.22)	43 (50.01)	2.38 (1.29-4.41)	0.0056	2.75 (1.31-5.77)	0.0074
Missing data	51 (2.31)	9 (16.96)	..	..	..	..
**Primary care physician**						
Without	131 (5.94)	10 (10.34)	1 (ref)	..	1 (ref)	..
With	2,073 (93.97)	667 (31.35)	3.96 (1.72-9.10)	0.0012	3.02 (1.26-7.26)	0.01
Missing data	2 (0.09)	1 (50.12)	..	..	..	..
**Screening invitation**						
Not received	529 (23.98)	63 (11.92)	1 (ref)	..	1 (ref)	..
Received	1,465 (66.41)	595 (38.88)	4.70 (3.18-6.93)	<0.0001	4.25 (2.74-6.58)	<0.0001
Missing data	212 (9.61)	20 (13.44)	..	..	..	..
**Gender**						
Man	823 (37.31)	270 (33.69)	1 (ref)	..	1 (ref)	..
Woman	1,383 (62.69)	408 (26.22)	0.70 (0.54-0.91)	0.0075	0.69 (0.52-0.92)	0.01
**Age**						
50-54	571 (25.88)	156 (25.85)	1 (ref)	..	1 (ref)	..
55-59	424 (19.22)	130 (29.17)	1.18 (0.79-1.77)	0.42	0.92 (0.58-1.46)	0.73
60-64	421 (19.08)	141 (35.20)	1.56 (1.05-2.32)	0.03	1.30 (0.84-2.01)	0.23
65-69	365 (16.55)	125 (38.56)	1.80 (1.17-2.76)	0.0070	1.54 (0.94-2.52)	0.09
70-74	425 (19.27)	126 (26.89)	1.06 (0.71-1.56)	0.79	0.85 (0.56-1.30)	0.46
**Socioeconomic profile**						
NS renter	1043 (47.28)	345 (30.79)	1 (ref)	..	1 (ref)	..
NS homeowner	311 (14.10)	105 (36.83)	1.31 (0.91-1.88)	0.14	1.40 (0.92-2.14)	0.12
Non-NS renter	828 (37.53)	223 (25.85)	0.78 (0.57-1.08)	0.13	0.96 (0.66-1.39)	0.81
Missing data[1]	24 (1.09)	5 (20.71)	..	..	..	..
**Arrondissement**						
1st	263 (11.92)	69 (29.01)	1 (ref)	..	1 (ref)	..
2nd	269 (12.19)	63 (25.81)	0.85 (0.45-1.61)	0.62	0.92 (0.45-1.87)	0.81
3rd	577 (26.16)	149 (26.82)	0.90 (0.56-1.44)	0.65	1.02 (0.59-1.76)	0.95
13th	344 (15.59)	139 (38.06)	1.50 (0.93-2.42)	0.09	1.68 (0.94-2.97)	0.08
14th	183 (8.30)	63 (36.82)	1.43 (0.82-2.48)	0.21	1.69 (0.88-3.24)	0.12
15th	332 (15.05)	120 (35.82)	1.37 (0.84-2.22)	0.21	1.68 (0.96-2.93)	0.09
16th	238 (10.79)	75 (34.30)	1.28 (0.77-2.12)	0.34	1.14 (0.65-2.00)	0.65

[1], Native speaker student considered as missing data because of to low frequence

7.3. Cervical cancer screening uptake

Supplementary table 5. Factors associated with up-to-date cervical cancer screening.

	n (%)	n up to date (weighted %)	Univariate analysis Odds ratio (95% CI)	p value	Multivariate analysis Odds ratio (95% CI)	p value
**Cervical cancer screening uptake** (women aged 25–64 years, n [without missing outcome] = 2,001, n [multivariate analysis] = 1,666)						
**Neighbourhoods by survey phase**						
T0 Control	586 (29.29)	404 (70.97)	1 (ref)	..	1 (ref)	..
T0 Intervention	538 (26.89)	394 (72.97)	1.10 (0.82-1.49)	0.52	1.10 (0.76-1.60)	0.62
T1 P1 Control	423 (21.14)	315 (75.02)	1.23 (0.87-1.73)	0.24	0.97 (0.64-1.46)	0.87
T1 P1 Intervention	253 (12.64)	180 (71.36)	1.02 (0.71-1.46)	0.92	0.85 (0.54-1.34)	0.49
T1 P2 Control	35 (1.75)	18 (55.07)	0.50 (0.24-1.06)	0.07	0.44 (0.19-1.00)	0.05
T1 P2 Intervention	145 (7.25)	82 (55.86)	0.52 (0.34-0.78)	0.0018	0.46 (0.27-0.79)	0.0050
Missing data	21 (1.05)	15 (83.21)	..	..	..	..
**Encounter with a health mediator**						
Not reported	1,884 (94.15)	1,319 (71.00)	1 (ref)	..	1 (ref)	..
Reported	87 (4.35)	72 (83.46)	2.06 (1.04-4.08)	0.04	1.79 (0.82-3.89)	0.14
Missing data	30 (1.50)	17 (67.75)	..	..	..	..
**Primary care physician**						
Without	169 (8.45)	96 (63.46)	1 (ref)	..	1 (ref)	..
With	1,830 (91.45)	1,311 (72.23)	1.50 (1.00-2.25)	0.05	1.39 (0.84-2.30)	0.19
Missing data	2 (0.10)	1 (50.12)	..	..	..	..
**Screening invitation**						
Not received	654 (32.68)	414 (64.04)	1 (ref)	..	1 (ref)	..
Received	1,055 (52.72)	836 (79.05)	2.12 (1.59-2.83)	<0.0001	2.20 (1.61-3.00)	<0.0001
Missing data	292 (14.59)	158 (56.46)	..	..	..	..
**Age**						
25-29	264 (13.19)	140 (55.00)	1 (ref)	..	1 (ref)	..
30-34	227 (11.34)	181 (81.39)	3.58 (2.08-6.16)	<0.0001	2.78 (1.43-5.41)	0.0026
35-39	254 (12.69)	201 (80.79)	3.44 (2.03-5.84)	<0.0001	2.29 (1.25-4.22)	0.0075
40-44	180 (9.00)	137 (71.59)	2.06 (1.20-3.53)	0.0084	1.33 (0.69-2.57)	0.39
45-49	210 (10.49)	162 (79.37)	3.15 (1.86-5.32)	<0.0001	1.95 (1.05-3.62)	0.04
50-54	356 (17.79)	259 (73.67)	2.29 (1.48-3.55)	0.0002	1.59 (0.93-2.71)	0.09
55-59	256 (12.79)	190 (72.06)	2.11 (1.25-3.55)	0.0050	1.44 (0.76-2.71)	0.26
60-64	254 (12.69)	138 (56.87)	1.08 (0.69-1.68)	0.74	0.72 (0.42-1.22)	0.22
**Socioeconomic profile**						
NS renter	902 (45.08)	660 (72.86)	1 (ref)	..	1 (ref)	..
NS homeowner	196 (9.80)	160 (84.61)	2.05 (1.26-3.34)	0.0040	1.94 (1.11-3.39)	0.02
NS student	100 (5.00)	34 (30.83)	0.17 (0.09-0.29)	<0.0001	0.21 (0.11-0.40)	<0.0001
Non-NS renter	802 (40.08)	553 (70.66)	0.90 (0.68-1.19)	0.45	1.01 (0.72-1.42)	0.95
Missing data	1 (0.05)	1 (100.00)	..	..	..	..
**Arrondissement**						
1st	219 (10.94)	159 (77.59)	1 (ref)	..	1 (ref)	..
2nd	231 (11.54)	173 (75.66)	0.90 (0.52-1.54)	0.70	0.77 (0.40-1.46)	0.42
3rd	555 (27.74)	394 (69.32)	0.65 (0.42-1.03)	0.06	0.57 (0.32-1.00)	0.05
13th	301 (15.04)	191 (64.42)	0.52 (0.33-0.83)	0.0066	0.56 (0.31-1.01)	0.05
14th	178 (8.90)	129 (73.58)	0.80 (0.47-1.38)	0.43	0.63 (0.34-1.18)	0.15
15th	291 (14.54)	180 (64.43)	0.52 (0.33-0.83)	0.0066	0.57 (0.31-1.03)	0.06
16th	226 (11.29)	182 (79.47)	1.12 (0.66-1.90)	0.68	0.92 (0.48-1.75)	0.79

APPENDIX 8. CANCER SCREENING AWARENESS

8.1. Colorectal cancer screening awareness

Supplementary table 6. Factors associated with awareness about colorectal cancer screening.

	n (%)	n aware (weighted %)	Univariate analysis Odds ratio (95% CI)	Univariate analysis p value	Multivariate analysis Odds ratio (95% CI)	Multivariate analysis p value
**Colorectal cancer screening awareness** (man and women aged 50-74 years, n [without missing outcome] = 2,273, n [multivariate analysis] =1,959)						
**Neighbourhoods by survey phase**						
T0 Control	655 (28.82)	562 (83.76)	1 (ref)	..	1 (ref)	..
T0 Intervention	572 (25.16)	456 (80.58)	0.80 (0.57-1.14)	0.23	1.01 (0.58-1.76)	0.98
T1 P1 Control	496 (21.82)	445 (87.28)	1.33 (0.79-2.23)	0.28	1.16 (0.62-2.14)	0.65
T1 P1 Intervention	308 (13.55)	227 (77.57)	0.67 (0.45-1.01)	0.05	0.57 (0.30-1.07)	0.08
T1 P2 Control	46 (2.02)	34 (74.21)	0.56 (0.25-1.27)	0.16	0.56 (0.23-1.34)	0.19
T1 P2 Intervention	174 (7.66)	141 (80.38)	0.79 (0.48-1.31)	0.36	0.88 (0.42-1.84)	0.73
Missing data	22 (0.97)	20 (92.15)	..	..	..	..
**Encounter with a health mediator**						
Not reported	2,125 (93.49)	1,753 (82.81)	1 (ref)	..	1 (ref)	..
Reported	96 (4.22)	86 (93.43)	2.95 (1.41-6.18)	0.0042	8.07 (2.10-30.96)	0.0023
Missing data	52 (2.29)	46 (88.28)	..	..	..	..
**Primary care physician**						
Without	137 (6.03)	91 (73.67)	1 (ref)	..	1 (ref)	..
With	2,133 (93.84)	1,792 (84.08)	1.89 (1.15-3.09)	0.01	0.89 (0.41-1.94)	0.77
Missing data	3 (0.13)	2 (88.65)	..	..	..	..
**Screening invitation**						
Not received	544 (23.93)	314 (56.49)	1 (ref)	..	1 (ref)	..
Received	1,486 (65.38)	1,438 (96.71)	22.67 (14.25-36.07)	<0.0001	20.77 (12.57-34.34)	<0.0001
Missing data	243 (10.69)	133 (62.19)	..	..	..	..
**Gender**						
Man	843 (37.09)	693 (82.77)	1 (ref)	..	1 (ref)	..
Woman	1,430 (62.91)	1,192 (83.93)	1.09 (0.78-1.52)	0.62	1.62 (1.02-2.57)	0.04
**Age**						
50-54	592 (26.04)	447 (77.46)	1 (ref)	..	1 (ref)	..
55-59	437 (19.23)	363 (85.81)	1.76 (1.12-2.77)	0.01	0.88 (0.48-1.61)	0.67
60-64	432 (19.01)	365 (85.42)	1.71 (1.08-2.68)	0.02	1.47 (0.81-2.68)	0.21
65-69	371 (16.32)	328 (90.20)	2.68 (1.60-4.48)	0.0002	1.78 (0.88-3.61)	0.11
70-74	441 (19.40)	382 (85.91)	1.78 (1.12-2.82)	0.01	0.91 (0.49-1.70)	0.76
**Socioeconomic profile**						
NS renter	1,074 (47.25)	949 (89.14)	1 (ref)	..	1 (ref)	..
NS homeowner	314 (13.81)	297 (93.93)	1.88 (0.96-3.70)	0.07	1.51 (0.66-3.45)	0.33
Non-NS renter	860 (37.84)	617 (70.81)	0.30 (0.21-0.42)	<0.0001	0.34 (0.21-0.54)	<0.0001
Missing data[1]	25 (1.10)	22 (91.18)	..	..	..	..
**Arrondissement**						
1st	267 (11.75)	221 (86.21)	1 (ref)	..	1 (ref)	..
2nd	274 (12.05)	237 (85.45)	0.94 (0.49-1.82)	0.85	0.86 (0.35-2.16)	0.76
3rd	597 (26.26)	471 (80.50)	0.66 (0.39-1.13)	0.13	0.70 (0.33-1.50)	0.36
13th	356 (15.66)	296 (83.52)	0.81 (0.47-1.40)	0.45	1.15 (0.50-2.65)	0.74
14th	185 (8.14)	151 (82.07)	0.73 (0.36-1.48)	0.38	0.90 (0.33-2.43)	0.83
15th	349 (15.35)	285 (81.53)	0.71 (0.41-1.21)	0.21	0.93 (0.41-2.12)	0.87
16th	245 (10.78)	224 (92.21)	1.89 (0.99-3.64)	0.06	1.20 (0.48-2.96)	0.70

[1], Native speaker student considered as missing data because of to low frequence

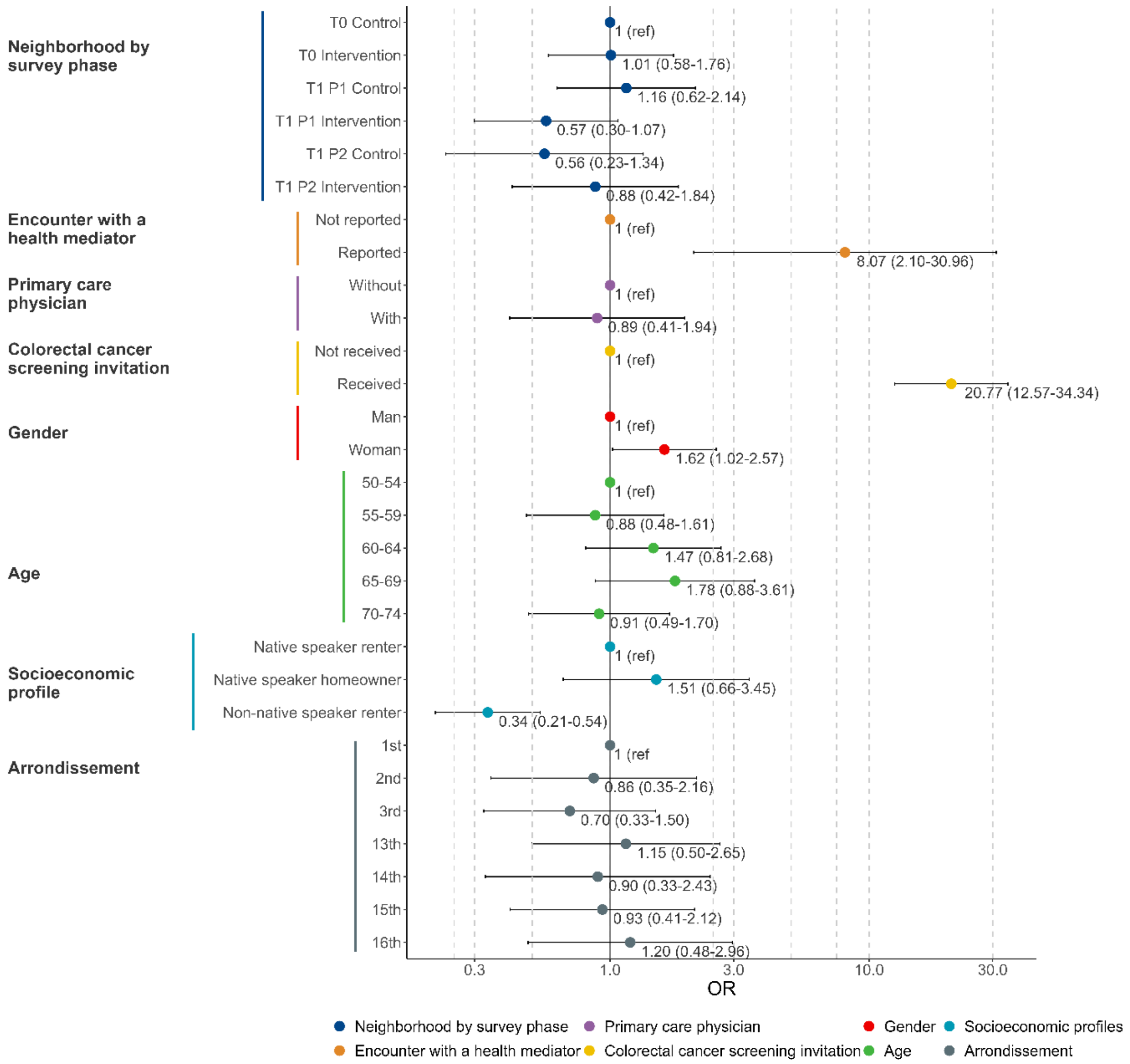

Supplementary figure 12. Factors associated with awareness about colorectal cancer screening in multivariate analysis (n=1,959).

8.2. Breast cancer screening awareness

Supplementary table 7. Factors associated with awareness about breast cancer screening.

	n (%)	n aware (weighted %)	Univariate analysis Odds ratio (95% CI)	p value	Multivariate analysis Odds ratio (95% CI)	p value
**Breast cancer screening awareness** (women aged 50-74 years, n [without missing outcome] = 1,432, n [multivariate analysis] =1,282)						
**Neighbourhoods by survey phase**						
T0 Control	392 (27.37)	379 (96.60)	1 (ref)	..	1 (ref)	..
T0 Intervention	335 (23.39)	312 (93.38)	0.50 (0.21-1.16)	0.10	0.85 (0.28-2.54)	0.77
T1 P1 Control	344 (24.02)	338 (98.75)	2.78 (0.92-8.37)	0.07	2.59 (0.81-8.27)	0.11
T1 P1 Intervention	188 (13.13)	177 (94.81)	0.64 (0.24-1.69)	0.37	0.52 (0.17-1.60)	0.25
T1 P2 Control	31 (2.16)	30 (97.55)	1.40 (0.17-11.78)	0.76	1.18 (0.12-11.98)	0.89
T1 P2 Intervention	130 (9.08)	120 (91.68)	0.39 (0.15-1.04)	0.06	0.58 (0.18-1.88)	0.36
Missing data	12 (0.84)	12 (100.00)	..	..	..	..
**Encounter with a health mediator**						
Not reported	1,333 (93.09)	1,272 (96.55)	1 (ref)	..	1 (ref)	..
Reported	62 (4.33)	61 (98.95)	3.38 (0.45-25.28)	0.23	3.24 (0.43-24.16)	0.25
Missing data	37 (2.58)	35 (96.95)	..	..	..	..
**Primary care physician**						
Without	68 (4.75)	58 (89.28)	1 (ref)	..	1 (ref)	..
With	1,361 (95.04)	1,307 (97.05)	3.95 (1.64-9.54)	0.0023	2.77 (1.03-7.48)	0.04
Missing data	3 (0.21)	3 (100.00)	..	..	..	..
**Screening invitation**						
Not received	193 (13.48)	175 (91.49)	1 (ref)	..	1 (ref)	..
Received	1,139 (79.54)	1,110 (98.08)	4.74 (2.17-10.37)	0.0001	4.08 (2.02-8.27)	0.0001
Missing data	100 (6.98)	83 (87.22)	..	..	..	..
**Age**						
50-54	383 (26.75)	363 (96.79)	1 (ref)	..	1 (ref)	..
55-59	282 (19.69)	270 (96.71)	0.98 (0.35-2.75)	0.96	0.90 (0.29-2.74)	0.85
60-64	271 (18.92)	257 (95.86)	0.77 (0.32-1.84)	0.55	0.52 (0.21-1.30)	0.16
65-69	229 (15.99)	224 (97.68)	1.39 (0.38-5.06)	0.61	0.83 (0.20-3.49)	0.80
70-74	267 (18.65)	254 (96.27)	0.86 (0.38-1.96)	0.71	0.80 (0.23-2.74)	0.72
**Socioeconomic profile**						
NS renter	679 (47.42)	661 (97.63)	1 (ref)	..	1 (ref)	..
NS homeowner	206 (14.39)	202 (98.84)	2.06 (0.58-7.36)	0.26	1.72 (0.39-7.50)	0.47
Non-NS renter	534 (37.29)	492 (94.05)	0.38 (0.18-0.82)	0.01	0.39 (0.16-0.96)	0.04
Missing data[1]	13 (0.91)	13 (100.00)	..	..	..	..
**Arrondissement**						
1st	150 (10.47)	139 (96.06)	1 (ref)	..	1 (ref)	..
2nd	182 (12.71)	175 (94.69)	0.73 (0.23-2.30)	0.59	0.68 (0.19-2.41)	0.55
3rd	382 (26.68)	371 (98.45)	2.62 (0.99-6.88)	0.05	3.37 (1.11-10.27)	0.03
13th	228 (15.92)	219 (96.86)	1.27 (0.49-3.28)	0.63	1.99 (0.60-6.62)	0.26
14th	118 (8.24)	116 (98.71)	3.14 (0.59-16.69)	0.18	5.66 (0.64-50.43)	0.12
15th	218 (15.22)	199 (91.09)	0.42 (0.18-0.97)	0.04	0.82 (0.28-2.40)	0.71
16th	154 (10.75)	149 (96.51)	1.14 (0.36-3.58)	0.83	1.02 (0.27-3.80)	0.98

[1], Native speaker student considered as missing data because of to low frequence

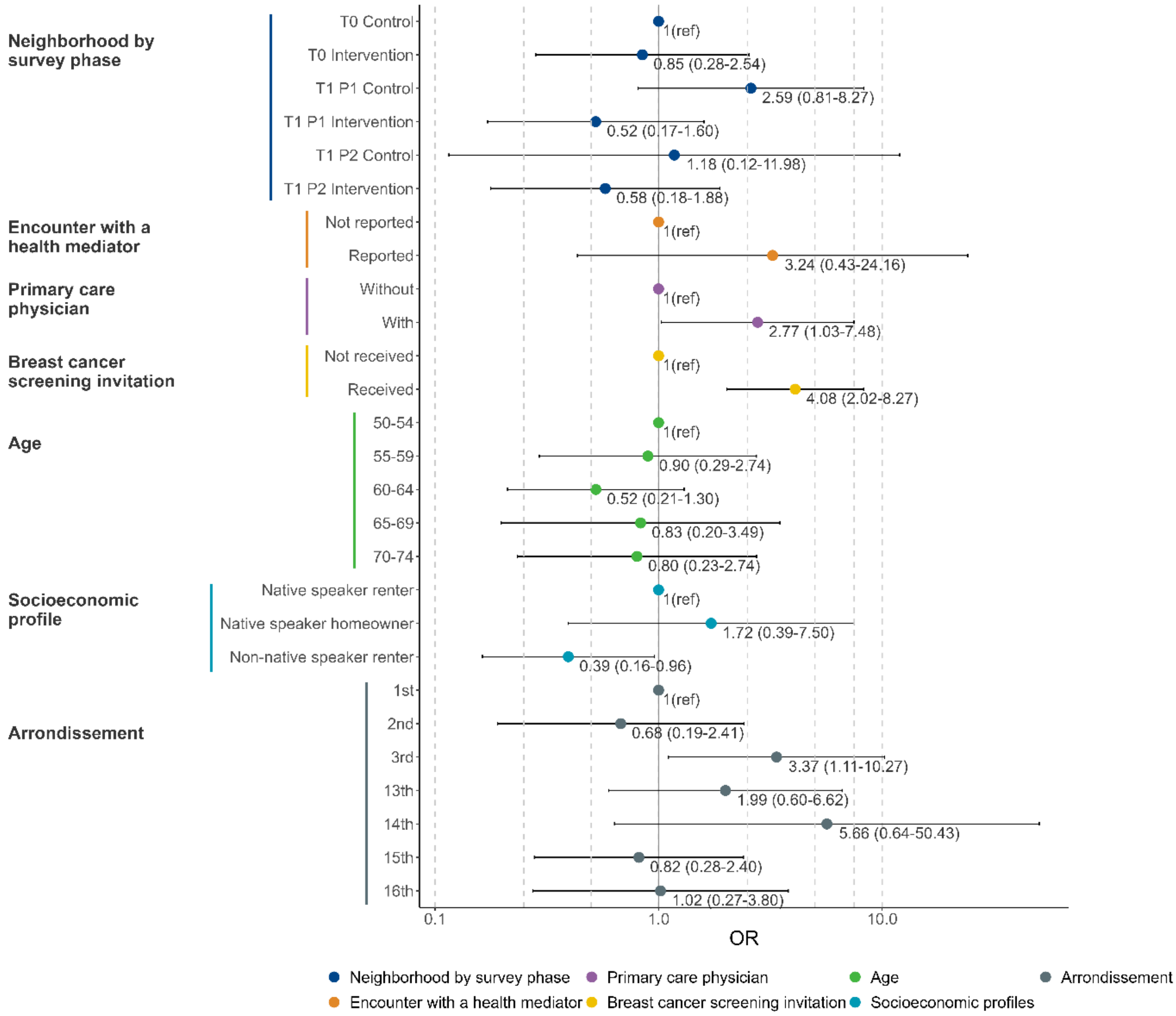

Supplementary figure 13. Factors associated with awareness about breast cancer screening in multivariate analysis (n=1,282).

8.3. Cervical cancer screening awareness

Supplementary table 8. Factors associated with awareness about cervical cancer screening.

	n (%)	n aware (weighted %)	Univariate analysis		Multivariate analysis	
			Odds ratio (95% CI)	p value	Odds ratio (95% CI)	p value
**Cervical cancer screening awareness** (women aged 25-64 years, n [without missing outcome] = 2,102, n [multivariate analysis] = 1,727)						
**Neighbourhoods by survey phase**						
T0 Control	618 (29.40)	524 (83.95)	1 (ref)	..	1 (ref)	..
T0 Intervention	577 (27.45)	472 (81.55)	0.85 (0.59-1.20)	0.35	1.09 (0.69-1.74)	0.71
T1 P1 Control	427 (20.31)	388 (91.49)	2.06 (1.26-3.34)	0.0037	2.10 (1.21-3.66)	0.0087
T1 P1 Intervention	263 (12.51)	237 (90.36)	1.79 (1.08-2.97)	0.02	1.64 (0.86-3.11)	0.13
T1 P2 Control	39 (1.86)	32 (81.86)	0.86 (0.35-2.11)	0.75	0.41 (0.15-1.14)	0.09
T1 P2 Intervention	157 (7.47)	139 (89.31)	1.60 (0.88-2.89)	0.12	1.31 (0.57-3.04)	0.53
Missing data	21 (1.00)	17 (90.84)	..	..	..	..
**Encounter with a health mediator**						
Not reported	1,983 (94.34)	1,700 (86.36)	1 (ref)	..	1 (ref)	..
Reported	88 (4.19)	82 (94.66)	2.80 (1.06-7.37)	0.04	2.24 (0.64-7.85)	0.21
Missing data	31 (1.47)	27 (93.50)	..	..	..	..
**Primary care physician**						
Without	175 (8.33)	130 (78.16)	1 (ref)	..	1 (ref)	..
With	1,924 (91.53)	1,677 (87.67)	1.99 (1.23-3.20)	0.0047	1.19 (0.66-2.16)	0.56
Missing data	3 (0.14)	2 (72.69)	..	..	..	..
**Screening invitation**						
Not received	685 (32.59)	521 (76.54)	1 (ref)	..	1 (ref)	..
Received	1,086 (51.67)	1,031 (94.28)	5.05 (3.34-7.64)	<0.0001	4.30 (2.78-6.65)	<0.0001
Missing data	331 (15.75)	257 (81.75)	..	..	..	..
**Age**						
25-29	274 (13.04)	226 (83.52)	1 (ref)	..	1 (ref)	..
30-34	234 (11.13)	195 (85.13)	1.13 (0.63-2.01)	0.68	1.09 (0.52-2.29)	0.82
35-39	259 (12.32)	226 (86.27)	1.24 (0.65-2.37)	0.52	1.08 (0.49-2.37)	0.85
40-44	188 (8.94)	159 (83.09)	0.97 (0.51-1.85)	0.93	0.86 (0.38-1.97)	0.73
45-49	213 (10.13)	185 (88.83)	1.57 (0.78-3.15)	0.20	1.79 (0.72-4.47)	0.21
50-54	379 (18.03)	336 (89.10)	1.61 (0.90-2.90)	0.11	1.72 (0.80-3.69)	0.16
55-59	283 (13.46)	249 (91.21)	2.05 (1.14-3.66)	0.02	2.32 (1.00-5.39)	0.05
60-64	272 (12.94)	233 (85.85)	1.20 (0.66-2.17)	0.55	1.18 (0.54-2.59)	0.67
**Socioeconomic profile**						
NS renter	947 (45.05)	870 (92.59)	1 (ref)	..	1 (ref)	..
NS homeowner	197 (9.37)	188 (95.62)	1.75 (0.72-4.22)	0.22	2.34 (0.88-6.19)	0.09
NS student	105 (5.00)	86 (78.99)	0.30 (0.15-0.60)	0.0007	0.61 (0.25-1.47)	0.27
Non-NS renter	852 (40.53)	664 (77.63)	0.28 (0.19-0.41)	<0.0001	0.26 (0.16-0.44)	<0.0001
Missing data	1 (0.05)	1 (100.00)	..	..	..	..
**Arrondissement**						
1st	225 (10.70)	192 (86.62)	1 (ref)	..	1 (ref)	..
2nd	236 (11.23)	217 (92.37)	1.87 (0.85-4.12)	0.12	2.07 (0.85-5.05)	0.11
3rd	572 (27.21)	472 (84.74)	0.86 (0.49-1.51)	0.60	1.12 (0.55-2.28)	0.75
13th	333 (15.84)	288 (86.44)	0.98 (0.54-1.80)	0.96	1.39 (0.65-2.97)	0.40
14th	188 (8.94)	164 (82.67)	0.74 (0.37-1.47)	0.39	0.66 (0.31-1.44)	0.30
15th	315 (14.99)	258 (83.69)	0.79 (0.44-1.43)	0.44	1.40 (0.64-3.07)	0.39
16th	233 (11.08)	218 (94.23)	2.52 (1.20-5.31)	0.01	2.90 (1.17-7.19)	0.02

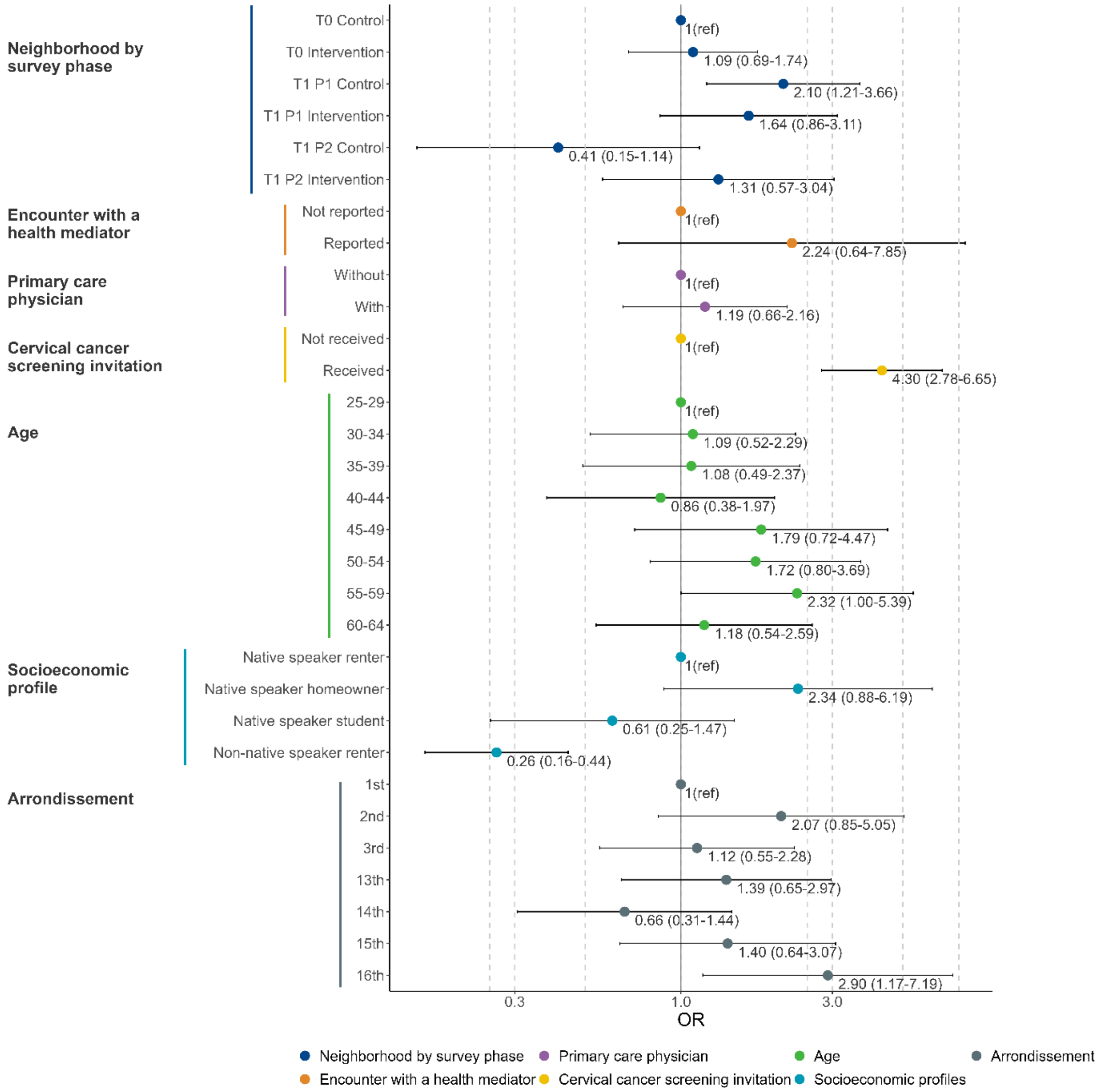

Supplementary figure 14. Factors associated with awareness about cervical cancer screening in multivariate analysis (n=1,727).

APPENDIX 9. CANCER SCREENING INVITATION

9.1. Breast cancer screening invitation

Supplementary table 9. Factors associated with reception of breast cancer screening invitation.

	n (%)	n receipt invitation (weighted %)	Univariate analysis Odds ratio (95% CI)	p value	Multivariate analysis Odds ratio (95% CI)	p value
**Breast cancer screening invitation reception** (women aged 50–74 years; n [receipt invitation] = 1,140 (WP=80.97%); n [without missing outcome] =1,334; n [multivariate analysis] = 1,284)						
**Neighbourhoods by survey phase**						
T0 Control	363 (27.21)	318 (87.32)	1 (ref)	..	1 (ref)	..
T0 Intervention	297 (22.26)	248 (82.47)	0.68 (0.42-1.12)	0.13	0.91 (0.53-1.56)	0.72
T1 P1 Control	333 (24.96)	284 (86.36)	0.92 (0.54-1.56)	0.76	1.11 (0.65-1.90)	0.70
T1 P1 Intervention	174 (13.04)	145 (82.34)	0.68 (0.39-1.19)	0.17	0.68 (0.36-1.28)	0.23
T1 P2 Control	28 (2.10)	21 (74.38)	0.42 (0.16-1.12)	0.08	0.30 (0.11-0.85)	0.02
T1 P2 Intervention	128 (9.60)	113 (87.46)	1.01 (0.51-2.01)	0.97	1.38 (0.61-3.11)	0.44
Missing data	11 (0.82)	11 (100.00)	..	..	..	..
**Encounter with a health mediator**						
Not reported	1,243 (93.18)	1,063 (85.73)	1 (ref)	..	1 (ref)	..
Reported	60 (4.50)	52 (88.91)	1.33 (0.60-2.99)	0.48	1.40 (0.60-3.31)	0.44
Missing data	31 (2.32)	25 (69.05)	..	..	..	..
**Primary care physician**						
Without	63 (4.72)	39 (63.50)	1 (ref)	..	1 (ref)	..
With	1,269 (95.13)	1,100 (86.68)	3.74 (1.85-7.57)	0.0003	4.46 (2.24-8.89)	<0.0001
Missing data	2 (0.15)	1 (50.12)	..	..	..	..
**Age**						
50-54	369 (27.66)	291 (80.77)	1 (ref)	..	1 (ref)	..
55-59	265 (19.87)	233 (89.29)	1.99 (1.13-3.47)	0.02	1.82 (0.99-3.36)	0.05
60-64	253 (18.97)	219 (85.23)	1.37 (0.79-2.40)	0.27	1.13 (0.63-2.04)	0.68
65-69	210 (15.74)	192 (89.99)	2.14 (1.12-4.07)	0.02	1.55 (0.80-3.00)	0.19
70-74	237 (17.77)	205 (88.08)	1.76 (0.97-3.18)	0.06	1.45 (0.81-2.61)	0.21
**Socioeconomic profile**						
NS renter	644 (48.28)	573 (89.55)	1 (ref)	..	1 (ref)	..
NS homeowner	202 (15.14)	183 (89.11)	0.95 (0.50-1.82)	0.89	0.90 (0.44-1.83)	0.77
Non-NS renter	478 (35.83)	376 (77.31)	0.40 (0.26-0.61)	0.0000	0.38 (0.24-0.60)	<0.0001
Missing data[1]	10 (0.75)	8 (90.63)	..	..	..	..
**Arrondissement**						
1st	140 (10.49)	122 (88.87)	1 (ref)	..	1 (ref)	..
2nd	172 (12.89)	149 (87.39)	0.87 (0.36-2.11)	0.75	1.10 (0.42-2.89)	0.85
3rd	363 (27.21)	296 (84.14)	0.66 (0.30-1.46)	0.31	1.07 (0.44-2.60)	0.87
13th	202 (15.14)	182 (88.41)	0.96 (0.40-2.26)	0.92	1.20 (0.47-3.06)	0.70
14th	112 (8.40)	94 (75.16)	0.38 (0.15-0.94)	0.04	0.44 (0.16-1.23)	0.12
15th	192 (14.39)	162 (81.61)	0.56 (0.24-1.27)	0.16	0.71 (0.28-1.80)	0.48
16th	153 (11.47)	135 (89.30)	1.05 (0.43-2.52)	0.92	1.09 (0.42-2.85)	0.86

[1], Native speaker student considered as missing data because of to low frequence

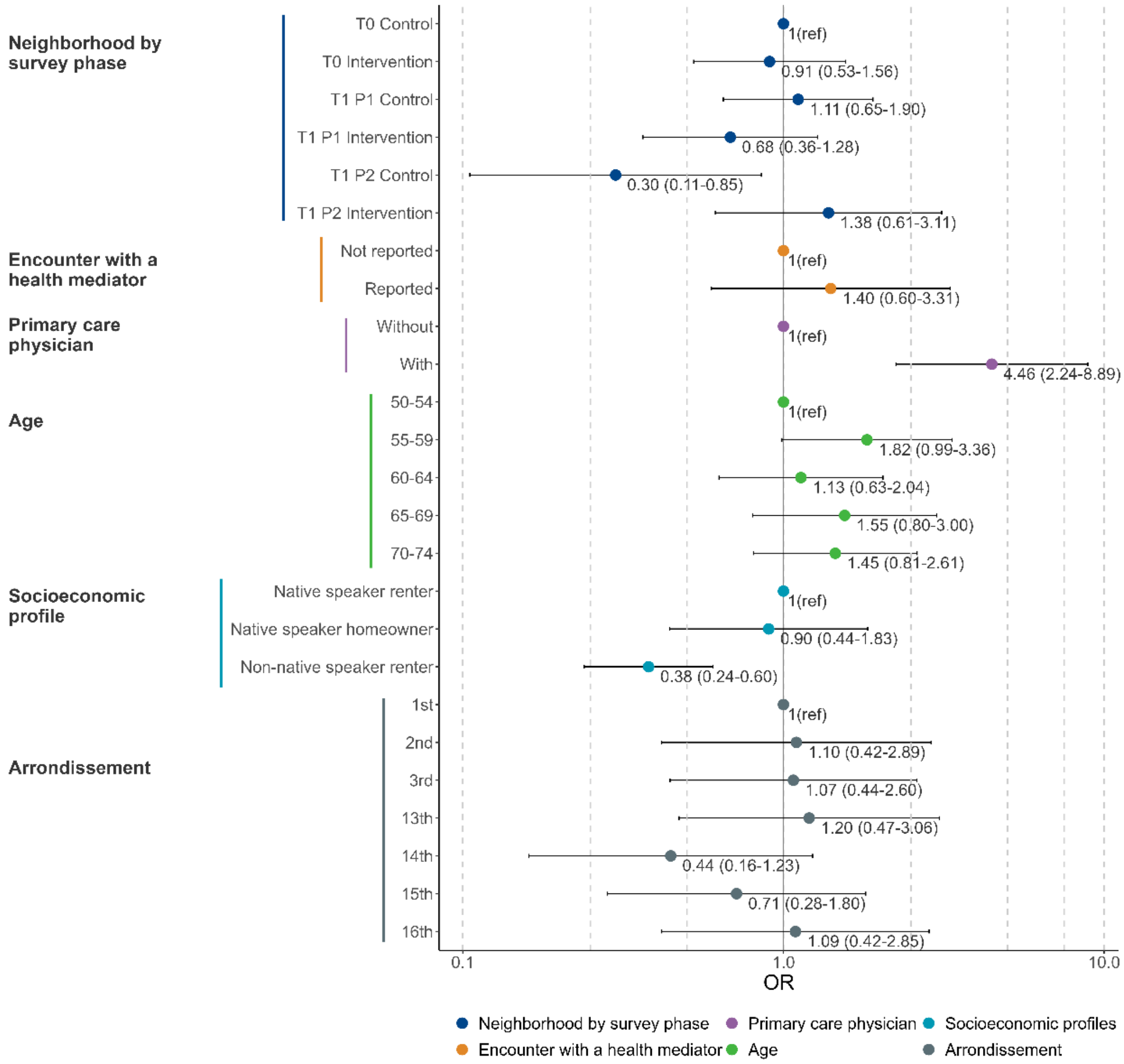

Supplementary figure 15. Factors associated with reception of breast cancer screening invitation, in the multivariate analysis (n=1,284).

9.2. Colorectal cancer screening invitation

Supplementary table 10. Factors associated with reception of colorectal cancer screening invitation.

	n (%)	N receipt invitation (weighted %)	Univariate analysis Odds ratio (95% CI)	p value	Multivariate analysis Odds ratio (95% CI)	p value
**Colorectal cancer screening invitation** (man and women aged 50–74 years; n [receipt invitation] = 1,488 (WP=65.15%); n [without missing outcome] = 2,034; n [multivariate analysis] = 1,963)						
**Neighbourhoods by survey phase**						
T0 Control	587 (28.86)	470 (75.85)	1 (ref)	..	1 (ref)	..
T0 Intervention	500 (24.58)	363 (73.41)	0.88 (0.62-1.24)	0.46	0.85 (0.59-1.23)	0.39
T1 P1 Control	453 (22.27)	329 (72.12)	0.82 (0.56-1.22)	0.33	0.86 (0.57-1.29)	0.46
T1 P1 Intervention	271 (13.32)	175 (66.56)	0.63 (0.43-0.94)	0.02	0.57 (0.36-0.92)	0.02
T1 P2 Control	45 (2.21)	25 (56.95)	0.42 (0.20-0.89)	0.02	0.35 (0.16-0.80)	0.01
T1 P2 Intervention	162 (7.96)	112 (66.90)	0.64 (0.41-1.01)	0.06	0.61 (0.37-1.00)	0.05
Missing data	16 (0.79)	14 (79.44)	..	..	..	..
**Encounter with a health mediator**						
Not reported	1,913 (94.05)	1,405 (72.71)	1 (ref)	..	1 (ref)	..
Reported	87 (4.28)	64 (64.76)	0.69 (0.32-1.47)	0.34	1.00 (0.42-2.38)	1
Missing data	34 (1.67)	19 (47.25)	..	..	..	..
**Primary care physician**						
Without	119 (5.85)	58 (48.36)	1 (ref)	..	1 (ref)	..
With	1,913 (94.05)	1,429 (73.73)	3.00 (1.79-5.00)	<0.0001	3.29 (1.85-5.85)	<0.0001
Missing data	2 (0.10)	1 (50.12)	..	..	..	..
**Gender**						
Man	758 (37.27)	579 (74.17)	1 (ref)	..	1 (ref)	..
Woman	1,276 (62.73)	909 (69.72)	0.80 (0.60-1.08)	0.14	0.69 (0.50-0.94)	0.02
**Age**						
50-54	522 (25.66)	310 (61.28)	1 (ref)	..	1 (ref)	..
55-59	391 (19.22)	297 (79.40)	2.44 (1.62-3.67)	<0.0001	2.05 (1.31-3.20)	0.0016
60-64	393 (19.32)	295 (73.25)	1.73 (1.15-2.61)	0.0087	1.49 (0.98-2.26)	0.06
65-69	336 (16.52)	270 (77.87)	2.22 (1.41-3.50)	0.0005	1.73 (1.06-2.81)	0.03
70-74	392 (19.27)	316 (81.03)	2.70 (1.78-4.08)	<0.0001	2.15 (1.40-3.29)	0.0004
**Socioeconomic profile**						
NS renter	974 (47.89)	772 (77.78)	1 (ref)	..	1 (ref)	..
NS homeowner	305 (15.00)	252 (85.21)	1.65 (1.05-2.57)	0.03	1.52 (0.94-2.46)	0.09
Non-NS renter	733 (36.04)	451 (57.75)	0.39 (0.28-0.54)	<0.0001	0.39 (0.28-0.55)	<0.0001
Missing data[1]	22 (1.08)	13 (61.05)	..	..	..	..
**Arrondissement**						
1st	248 (12.19)	182 (75.93)	1 (ref)	..	1 (ref)	..
2nd	255 (12.54)	192 (75.73)	0.99 (0.57-1.72)	0.97	1.07 (0.56-2.04)	0.84
3rd	544 (26.75)	350 (66.07)	0.62 (0.39-0.99)	0.04	0.78 (0.46-1.32)	0.35
13th	302 (14.85)	233 (75.72)	0.99 (0.60-1.64)	0.96	1.29 (0.73-2.28)	0.38
14th	160 (7.87)	121 (71.75)	0.80 (0.43-1.51)	0.50	0.99 (0.45-2.15)	0.97
15th	292 (14.36)	226 (74.37)	0.92 (0.56-1.52)	0.74	1.12 (0.64-1.95)	0.69
16th	233 (11.46)	184 (81.28)	1.38 (0.81-2.33)	0.23	1.23 (0.69-2.20)	0.48

[1], Native speaker student considered as missing data because of to low frequence

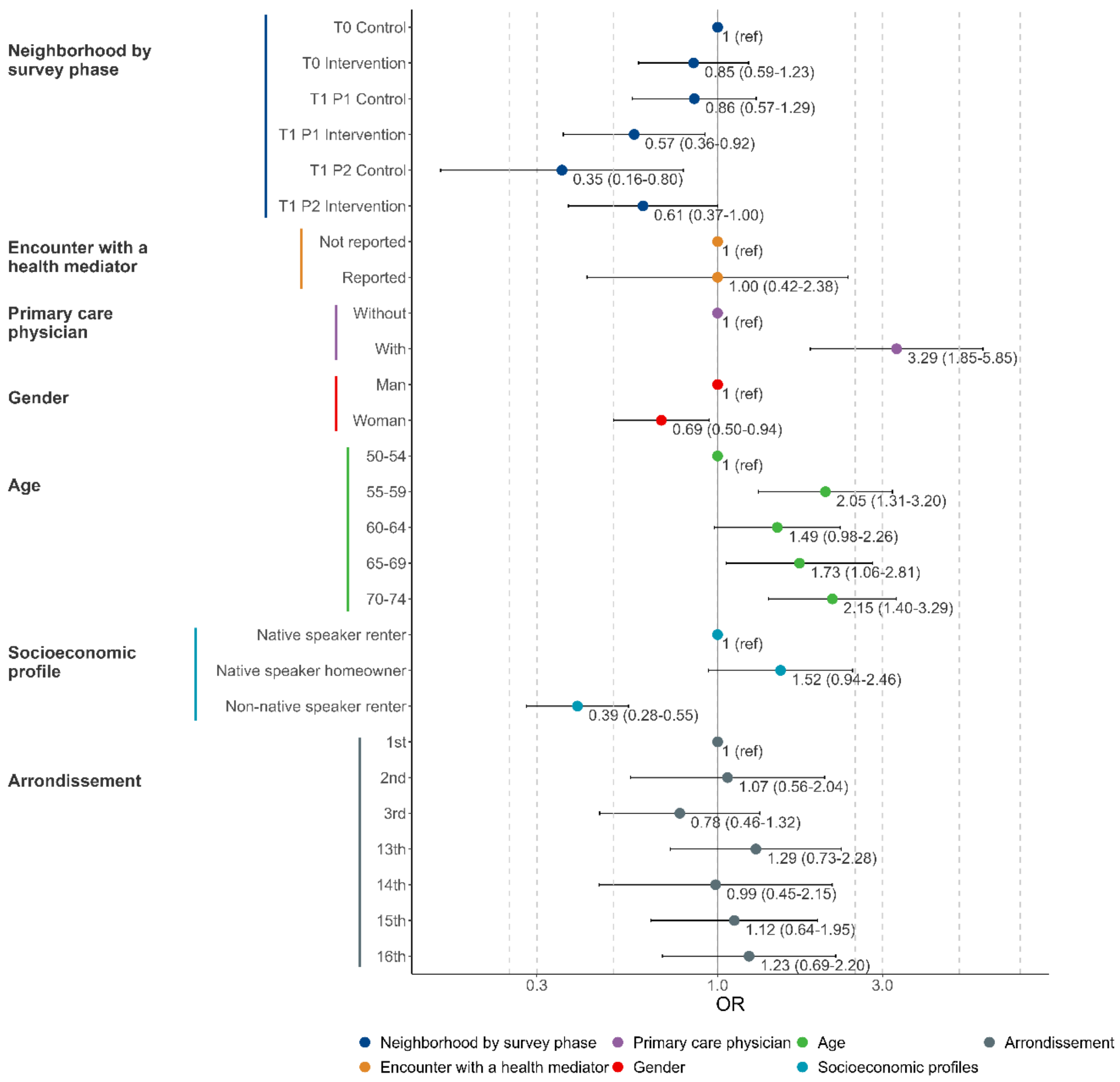

Supplementary figure 16. Factors associated with colorectal cancer screening invitation reception in multivariate analysis (n=1,963).

9.3. Cervical cancer screening invitation

Supplementary table 11. Factors associated with reception of cervical cancer screening invitation.

	n (%)	n receipt invitation (weighted %)	Univariate analysis Odds ratio (95% CI)	p value	Multivariate analysis Odds ratio (95% CI)	p value
**Cervical cancer screening invitation** (women aged 25–64 years; n [receipt invitation] = 1,088 (WP=53.30%); n [without missing outcome] = 1,775; n; n [multivariate analysis] = 1,731)						
**Neighbourhoods by survey phase**						
T0 Control	487 (27.44)	300 (59.27)	1 (ref)	..	1 (ref)	..
T0 Intervention	464 (26.14)	239 (51.13)	0.72 (0.53-0.97)	0.03	0.71 (0.51-0.98)	0.04
T1 P1 Control	393 (22.14)	258 (66.00)	1.33 (0.95-1.87)	0.09	1.37 (0.96-1.95)	0.08
T1 P1 Intervention	242 (13.63)	158 (64.95)	1.27 (0.89-1.82)	0.18	1.07 (0.72-1.60)	0.74
T1 P2 Control	36 (2.03)	17 (54.60)	0.83 (0.40-1.73)	0.61	0.68 (0.32-1.44)	0.31
T1 P2 Intervention	135 (7.61)	103 (76.32)	2.21 (1.37-3.57)	0.0011	1.79 (1.04-3.07)	0.04
Missing data	18 (1.01)	13 (59.26)	..	..	..	..
**Encounter with a health mediator**						
Not reported	1,668 (93.97)	1,008 (60.93)	1 (ref)	..	1 (ref)	..
Reported	84 (4.73)	65 (78.80)	2.38 (1.28-4.43)	0.0061	2.00 (1.03-3.88)	0.04
Missing data	23 (1.30)	15 (49.51)	..	..	..	..
**Primary care physician**						
Without	146 (8.23)	57 (40.21)	1 (ref)	..	1 (ref)	..
With	1,627 (91.66)	1,030 (63.63)	2.60 (1.68-4.02)	<0.0001	2.57 (1.59-4.14)	0.0001
Missing data	2 (0.11)	1 (50.12)	..	..	..	..
**Age**						
25-29	237 (13.35)	131 (53.01)	1 (ref)	..	1 (ref)	..
30-34	197 (11.10)	126 (64.45)	1.61 (0.97-2.66)	0.07	1.80 (1.03-3.14)	0.04
35-39	229 (12.90)	143 (64.11)	1.58 (0.95-2.63)	0.08	1.84 (1.05-3.24)	0.03
40-44	167 (9.41)	102 (61.16)	1.40 (0.83-2.34)	0.21	1.61 (0.90-2.91)	0.11
45-49	186 (10.48)	103 (57.29)	1.19 (0.70-2.01)	0.52	1.26 (0.69-2.27)	0.45
50-54	315 (17.75)	198 (64.96)	1.64 (1.05-2.56)	0.03	1.88 (1.15-3.07)	0.01
55-59	230 (12.96)	150 (66.79)	1.78 (1.10-2.89)	0.02	1.81 (1.07-3.07)	0.03
60-64	214 (12.06)	135 (60.02)	1.33 (0.82-2.15)	0.24	1.25 (0.73-2.12)	0.41
**Socioeconomic profile**						
NS renter	809 (45.58)	569 (69.33)	1 (ref)	..	1 (ref)	..
NS homeowner	179 (10.08)	112 (60.83)	0.69 (0.45-1.05)	0.08	0.64 (0.41-1.00)	0.05
NS student	87 (4.90)	45 (55.25)	0.55 (0.31-0.97)	0.04	0.72 (0.39-1.35)	0.31
Non-NS renter	699 (39.38)	361 (52.41)	0.49 (0.37-0.65)	<0.0001	0.45 (0.33-0.61)	<0.0001
Missing data	1 (0.06)	1 (100.00)	..	..	..	..
**Arrondissement**						
1st	199 (11.21)	125 (61.21)	1 (ref)	..	1 (ref)	..
2nd	219 (12.34)	135 (64.22)	1.14 (0.67-1.94)	0.63	1.12 (0.61-2.03)	0.72
3rd	515 (29.01)	275 (57.85)	0.87 (0.55-1.37)	0.55	0.92 (0.54-1.58)	0.76
13th	259 (14.59)	178 (67.67)	1.33 (0.81-2.17)	0.26	1.17 (0.66-2.07)	0.59
14th	173 (9.75)	121 (65.73)	1.22 (0.71-2.09)	0.48	1.15 (0.63-2.11)	0.65
15th	213 (12.00)	140 (67.06)	1.29 (0.78-2.14)	0.32	1.16 (0.65-2.05)	0.61
16th	197 (11.10)	114 (60.75)	0.98 (0.59-1.63)	0.94	0.82 (0.46-1.48)	0.52

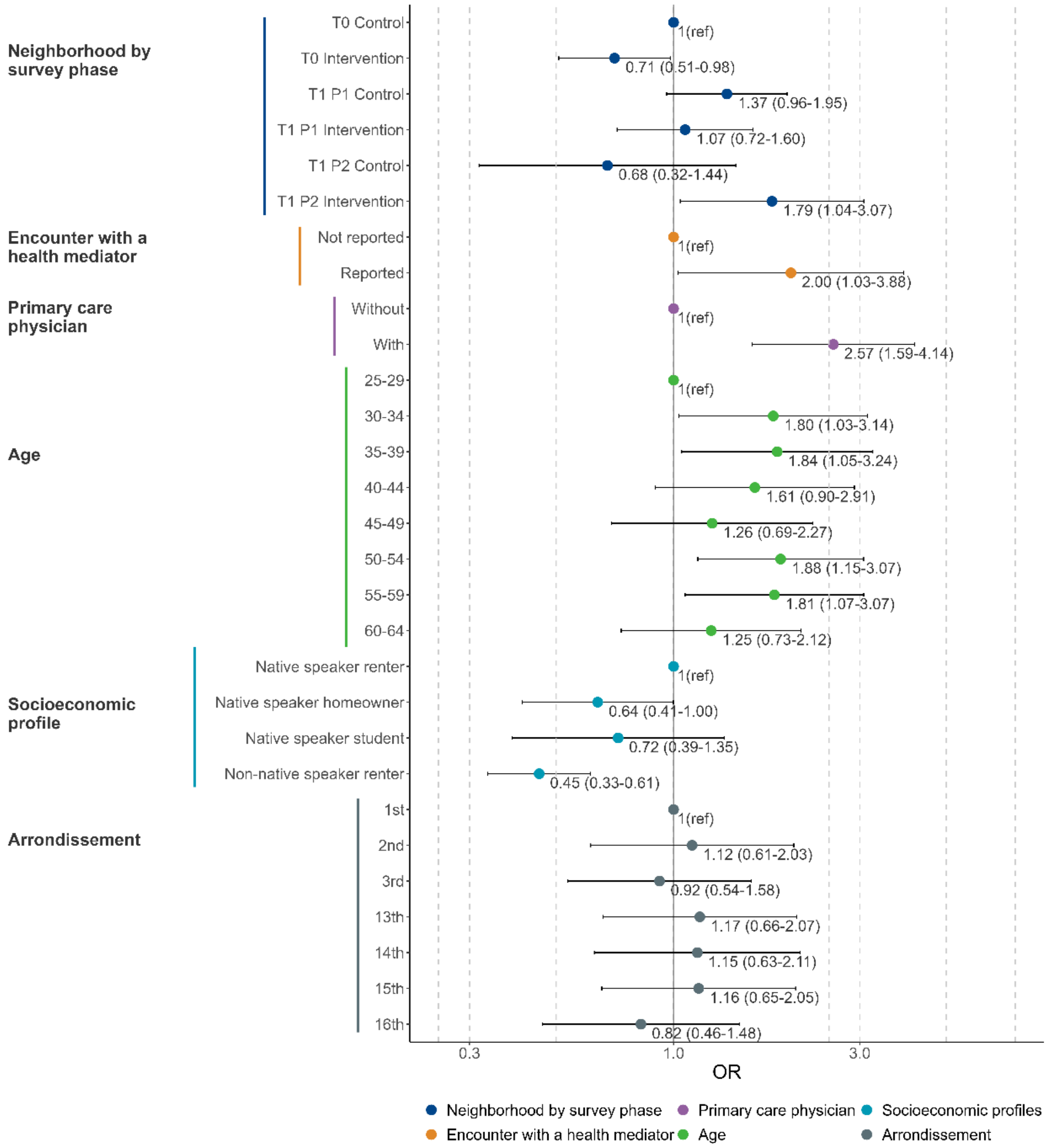

Supplementary figure 12. Factors associated with cervical cancer screening invitation reception in multivariate analysis (n=1,731).

APPENDIX 10. PRIMARY CARE PHYSICIAN

Participants surveyed at T1 (T1 P1 control: OR = 2.11 [1.43-3.10], T1 P1 intervention: OR = 3.12 [1.82-5.33], T1 P2 control: OR = 3.70 (1.17-11.75), women (OR = 1.91 [1.45-2.51]) and participants aged over 40 years (from 40-44 years : OR = 3.15 [1.52-6.53] to 70-74 years : OR = 4.73 [2.00-11.18]) declared a higher access to a primary care physician than participants of the T0 survey, men and those aged lower than 40 years, respectively.

Supplementary table 12. Factors associated with access to a primary care physician.

	n (%)	N with physician (weighted %)	Univariate analysis Odds ratio (95% CI)	p value	Multivariate analysis Odds ratio (95% CI)	p value
**Primary care physician** (n [with primary care physician] = 4 000 (WP = 85.14%); n [without missing outcome] = 4,509; n [multivariable analysis] =4,401)						
**Neighbourhoods by survey phase**						
T0 Control	1,379 (30.58)	1,183 (80.63)	1 (ref)	..	1 (ref)	..
T0 Intervention	1,260 (27.94)	1,085 (82.61)	1.14 (0.88-1.48)	0.32	1.12 (0.84-1.50)	0.42
T1 P1 Control	884 (19.61)	806 (88.98)	1.94 (1.37-2.74)	0.0002	2.11 (1.43-3.10)	0.0002
T1 P1 Intervention	580 (12.86)	550 (92.77)	3.08 (1.92-4.95)	<0.0001	3.12 (1.82-5.33)	<0.0001
T1 P2 Control	81 (1.80)	77 (91.11)	2.46 (0.86-7.00)	0.09	3.70 (1.17-11.75)	0.03
T1 P2 Intervention	281 (6.23)	260 (88.58)	1.86 (1.07-3.23)	0.03	1.73 (0.94-3.19)	0.08
Missing data	44 (0.98)	39 (89.15)	..	..	..	..
**Encounter with a health mediator**						
Not reported	4,280 (94.92)	3,785 (85.25)	1 (ref)	..		
Reported	162 (3.59)	155 (95.03)	3.31 (1.28-8.57)	0.0137	1.82 (0.61-5.44)	0.28
Missing data	67 (1.49)	60 (83.01)	..	..		
**Gender**						
Man	1,681 (37.28)	1,401 (81.79)	1 (ref)	..	1 (ref)	..
Woman	2,828 (62.72)	2,599 (89.11)	1.82 (1.42-2.34)	<0.0001	1.91 (1.45-2.51)	<0.0001
**Age**						
18-24	427 (9.47)	339 (78.85)	1 (ref)	..	1 (ref)	..
25-29	436 (9.67)	329 (71.81)	0.68 (0.45-1.05)	0.08	0.64 (0.39-1.04)	0.07
30-34	351 (7.78)	287 (79.73)	1.06 (0.65-1.72)	0.83	1.01 (0.57-1.82)	0.96
35-39	391 (8.67)	334 (81.51)	1.18 (0.73-1.93)	0.50	1.09 (0.60-1.99)	0.77
40-44	301 (6.68)	276 (91.20)	2.78 (1.52-5.09)	0.0009	3.15 (1.52-6.53)	0.0020
45-49	325 (7.21)	295 (89.05)	2.18 (1.24-3.84)	0.0070	2.22 (1.17-4.21)	0.01
50-54	593 (13.15)	536 (89.37)	2.26 (1.41-3.61)	0.0007	1.89 (1.04-3.42)	0.04
55-59	439 (9.74)	408 (93.82)	4.07 (2.30-7.23)	<0.0001	3.80 (1.94-7.44)	0.0001
60-64	433 (9.60)	412 (94.47)	4.58 (2.03-10.35)	0.0003	3.92 (1.64-9.37)	0.0021
65-69	372 (8.25)	359 (96.28)	6.94 (3.35-14.40)	<0.0001	5.73 (2.57-12.79)	<0.0001
70-74	441 (9.78)	425 (95.19)	5.31 (2.49-11.34)	<0.0001	4.73 (2.00-11.18)	0.0004
**Socioeconomic profile**						
NS renter	1,934 (42.89)	1,764 (88.81)	1 (ref)	..	1 (ref)	..
NS homeowner	492 (10.91)	452 (92.00)	1.45 (0.93-2.27)	0.10	1.42 (0.85-2.38)	0.18
NS student	476 (10.56)	379 (78.66)	0.46 (0.32-0.68)	0.0001	0.71 (0.44-1.16)	0.17
Non-NS renter	1,605 (35.60)	1,403 (82.07)	0.58 (0.43-0.77)	0.0002	0.58 (0.42-0.79)	0.0007
Missing data	2 (0.04)	2 (100.00)	..	..	..	..
**Arrondissement**						
1st	531 (11.78)	437 (80.26)	1 (ref)	..	1 (ref)	..
2nd	540 (11.98)	474 (88.02)	1.81 (1.17-2.80)	0.0080	1.84 (1.12-3.01)	0.02
3rd	1,192 (26.44)	1,013 (82.13)	1.13 (0.79-1.61)	0.50	1.22 (0.80-1.86)	0.36
13th	695 (15.41)	658 (93.67)	3.64 (2.20-6.02)	<0.0001	3.80 (2.19-6.60)	<0.0001
14th	395 (8.76)	353 (87.82)	1.77 (1.12-2.81)	0.01	1.83 (1.10-3.03)	0.02
15th	663 (14.70)	614 (90.83)	2.44 (1.56-3.80)	0.0001	2.83 (1.72-4.66)	<0.0001
16th	493 (10.93)	451 (90.67)	2.39 (1.52-3.76)	0.0002	2.00 (1.19-3.34)	0.0085

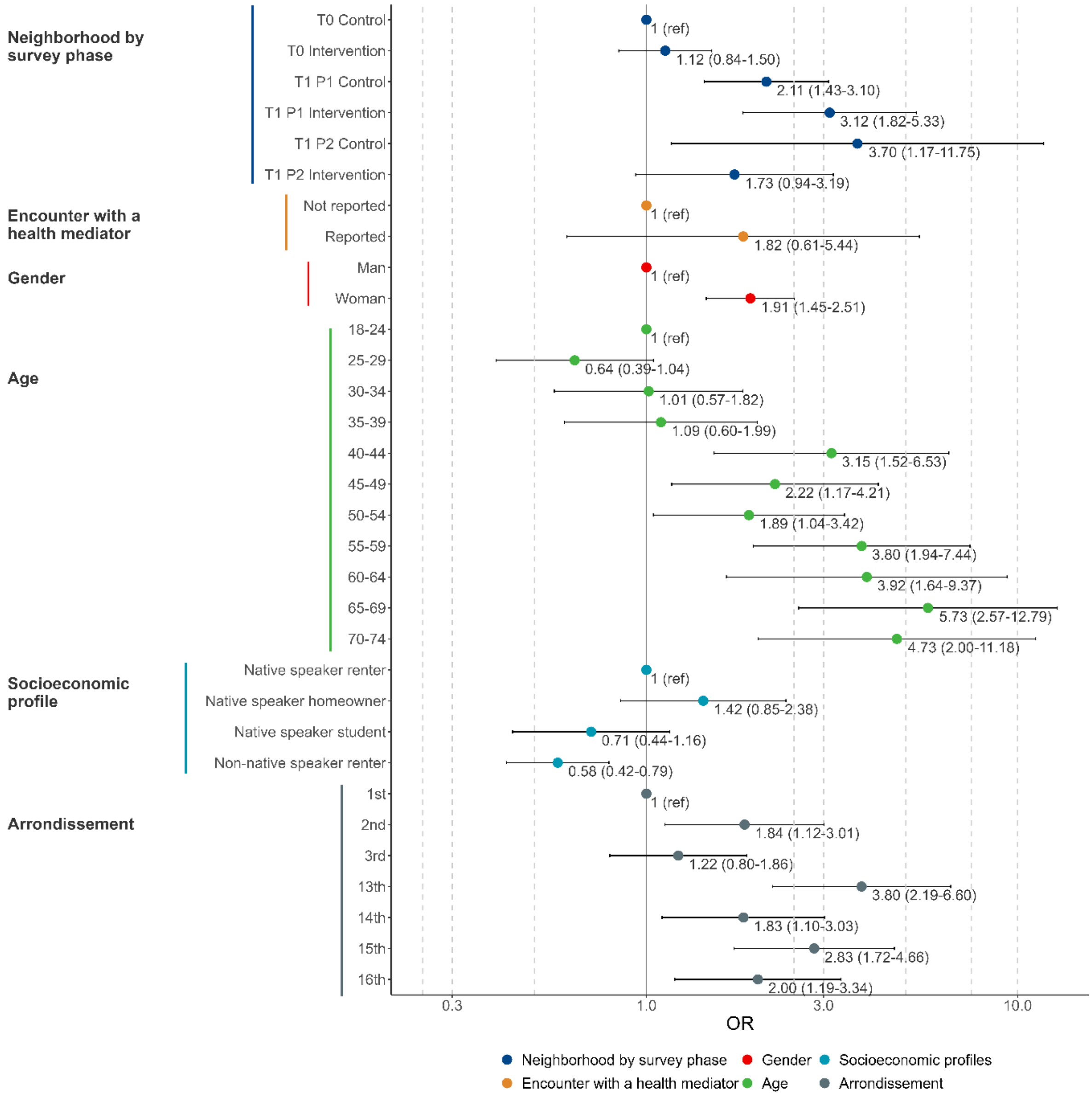

Supplementary figure 18. Factors associated with individuals with a primary care physician, in the weighted multivariate analysis (n=4,401).
